# TFEB and TFE3 control glucose homeostasis by regulating insulin gene expression

**DOI:** 10.15252/embj.2023113928

**Published:** 2023-09-15

**Authors:** Adrien Pasquier, Nunzia Pastore, Luca D'Orsi, Rita Colonna, Alessandra Esposito, Veronica Maffia, Rossella De Cegli, Margherita Mutarelli, Susanna Ambrosio, Gennaro Tufano, Antonio Grimaldi, Marcella Cesana, Davide Cacchiarelli, Nathalie Delalleau, Gennaro Napolitano, Andrea Ballabio

**Affiliations:** ^1^ Telethon Institute of Genetics and Medicine (TIGEM) Naples Italy; ^2^ Medical Genetics Unit, Department of Medical and Translational Science Federico II University Naples Italy; ^3^ Institute of Applied Sciences and Intelligent Systems National Research Council (ISASI‐CNR) Pozzuoli Italy; ^4^ School for Advanced Studies, Genomics and Experimental Medicine Program University of Naples "Federico II" Naples Italy; ^5^ University of Lille, U1190‐EGID Lille France; ^6^ Inserm, U1190 Lille France; ^7^ Department of Molecular and Human Genetics Baylor College of Medicine Houston TX USA; ^8^ Jan and Dan Duncan Neurological Research Institute Texas Children's Hospital Houston TX USA

**Keywords:** beta cells, glucose homeostasis, insulin, mTORC1, TFEB, Chromatin, Transcription & Genomics, Metabolism

## Abstract

To fulfill their function, pancreatic beta cells require precise nutrient‐sensing mechanisms that control insulin production. Transcription factor EB (TFEB) and its homolog TFE3 have emerged as crucial regulators of the adaptive response of cell metabolism to environmental cues. Here, we show that TFEB and TFE3 regulate beta‐cell function and insulin gene expression in response to variations in nutrient availability. We found that nutrient deprivation in beta cells promoted TFEB/TFE3 activation, which resulted in suppression of insulin gene expression. TFEB overexpression was sufficient to inhibit insulin transcription, whereas beta cells depleted of both TFEB and TFE3 failed to suppress insulin gene expression in response to amino acid deprivation. Interestingly, ChIP‐seq analysis showed binding of TFEB to super‐enhancer regions that regulate insulin transcription. Conditional, beta‐cell‐specific, *Tfeb*‐overexpressing, and *Tfeb*/*Tfe3* double‐KO mice showed severe alteration of insulin transcription, secretion, and glucose tolerance, indicating that TFEB and TFE3 are important physiological mediators of pancreatic function. Our findings reveal a nutrient‐controlled transcriptional mechanism that regulates insulin production, thus playing a key role in glucose homeostasis at both cellular and organismal levels.

## Graphical abstract

TFEB and its homologue TFE3 are novel regulators of beta‐cells and pancreas function in the mouse controlling nutrient‐dependent insulin production.

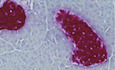

## Introduction

Pancreatic β‐cells play a crucial role in organismal adaptation to nutrient availability by secreting the central hormone insulin. Consistently, β‐cell failure results in dysregulation of glucose homeostasis and is the hallmark of diabetes (Cnop *et al*, 2005; Vasiljevic *et al*, 2020; Gloyn *et al*, 2022). Thus, understanding how β‐cells orchestrate the metabolic response to nutrient availability is of pivotal importance. The nutrient‐sensitive transcription factor EB (TFEB), as well as TFE3, another member of the MiT/TFE family, are major controllers of cell metabolism (Napolitano & Ballabio, 2016; Puertollano *et al*, 2018). TFEB and TFE3 regulate cell metabolism at multiple levels, such as lysosomal biogenesis (Sardiello *et al*, 2009), autophagy (Settembre *et al*, 2011), lipid catabolism (Settembre *et al*, 2013; Pastore *et al*, 2017), circadian rhythm (Pastore *et al*, 2019), mitochondrial biogenesis (Mansueto *et al*, 2017), as well as whole energy metabolism (Pastore *et al*, 2017). TFEB and TFE3 subcellular localization and activities are regulated by the mechanistic target of rapamycin complex 1 (mTORC1; Martina *et al*, 2012, 2014; Roczniak‐Ferguson *et al*, 2012; Settembre *et al*, 2012), which is recruited and activated at the lysosomal surface in response to increased levels of amino acids (Sancak *et al*, 2008, 2010), glucose (Orozco *et al*, 2020; Yoon *et al*, 2020), and other nutrients (Castellano *et al*, 2017; Liu & Sabatini, 2020). Gain‐ and loss‐of‐function experiments in mice have shown that mTORC1 activity is crucial for maintaining pancreatic β‐cell homeostasis by modulating insulin mRNA translation and β‐cell mass (Rachdi *et al*, 2008; Mori *et al*, 2009; Blandino‐Rosano *et al*, 2017). mTORC1 inhibition during amino acid and glucose starvation leads to TFEB/TFE3 dephosphorylation, cytoplasm‐to‐nucleus translocation, and activation of their transcriptional program. Previous studies showed that TFEB/TFE3 transcriptional programs vary in different cell types, suggesting specialized functions of these transcription factors in different tissues (Napolitano & Ballabio, 2016). However, whether TFEB and TFE3 play specific roles in the maintenance of pancreatic endocrine function, which is crucial for organismal metabolism and adaptation to nutrient availability, has not been recognized thus far.

Here, we show that TFEB and TFE3 modulate insulin gene expression in response to changes in nutrient availability. We found that TFEB overexpression was sufficient to suppress insulin transcription, whereas β‐cells lacking both TFEB and TFE3 failed to suppress insulin gene expression in response to nutrient starvation, both *in vitro* and *in vivo*, resulting in impaired glucose homeostasis. Thus, in addition to their well‐established role in promoting cell catabolic pathways, our data reveal that TFEB and TFE3 also function as suppressors of cell anabolism via inhibition of insulin production.

## Results

### TFEB and TFE3 activity is controlled by nutrient availability and mTORC1 activity in pancreatic β‐cells

**Figure 1:**
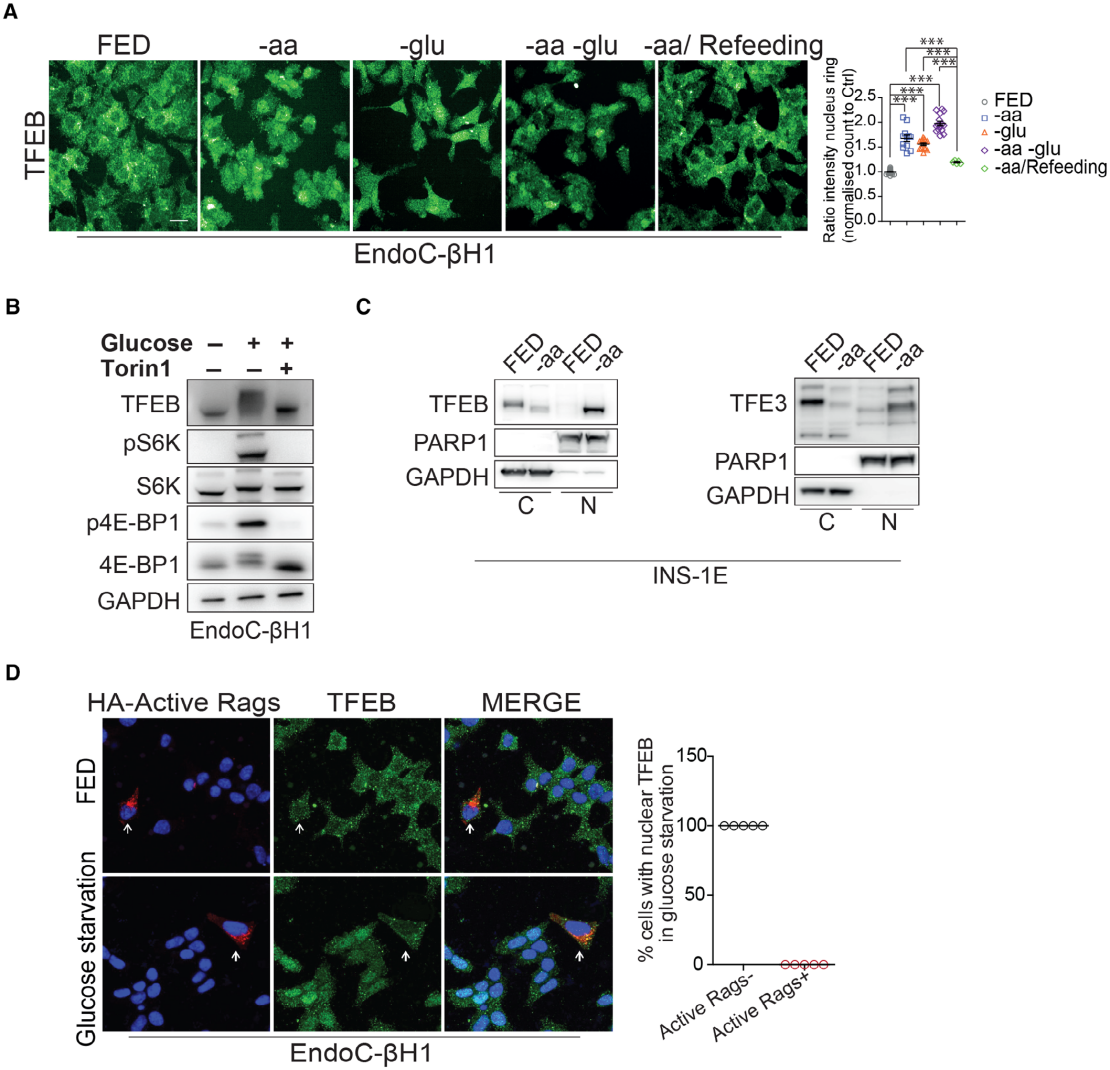
TFEB responds to nutrient variation in pancreatic β‐cells ARepresentative images of high content analyses using TFEB antibodies on fixed cells previously treated with full medium (FED), without amino acids (−aa) for 1 h, without glucose (−glu) for 1 h, without amino acids and glucose (−aa ‐glu) for 1 h or starved for 1 h and restimulated with amino acids for 30′ (−aa/Refeeding). The relative quantification (see Materials and Methods) is shown in the graph. Data are represented as mean ± standard error. Each dot represents a well from the 96‐well plate (*n* = 30 fed, *n* = 12 −aa, *n* = 14 −glu, *n* = 15 −aa −glu, and *n* = 5 −aa/Refeeding). The experiment was repeated independently three different times. Scale bar: 20 μm. Student's two‐tailed *t*‐test: ***P‐value < 0.001.BRepresentative immunoblot using the indicated antibodies from lysates of EndoC‐βH1 cells starved for 1 h of glucose or starved for 1 h and restimulated for 30′ with glucose in the presence or absence of Torin1.CRepresentative immunoblot for TFEB and TFE3 using lysates from nuclear and cytosolic fractions of INS‐1E cells treated with full medium (FED) or upon amino acid starvation (−aa). PARP1 was used as nuclear fraction loading control, and GAPDH was used as cytosol fraction loading control. Cytosolic fraction (C), Nuclear fraction (N).DRepresentative immunofluorescence analysis using TFEB antibodies on fixed EndoC‐βH1‐cells treated with full medium (FED) or upon glucose starvation, prior transient transfection with constitutively active Rag GTPase mutants (i.e., HA‐GST‐RagA‐Q66L; HA‐GST‐RagC‐S75L). Transfected cells are indicated with a white arrow. Data are represented as mean ± standard error. Each dot represents a separate field of a representative experiment (*n* = 5). At least 52 cells were counted in each condition. Source data are available online for this figure.

To investigate the role of TFEB and TFE3 in pancreatic β‐cells, we analyzed the regulation and downstream transcriptional programs of these transcription factors in the human EndoC‐βH1 and rat INS‐1E β‐cell lines (Ravassard *et al*, 2011; Tsonkova *et al*, 2018). Similar to other cell types (Settembre *et al*, 2011, 2012; Martina *et al*, 2012; Li *et al*, 2018; Napolitano *et al*, 2018), TFEB localized to the cytosol of fully fed or refed EndoC‐βH1 cells, whereas it translocated to the nucleus upon either amino acid or glucose starvation (Fig 1A). Consistently, phosphorylation of endogenous TFEB in EndoC‐βH1 cells, which was measured by analyzing its molecular weight shift (Settembre *et al*, 2012; Napolitano *et al*, 2020), was dependent on both nutrient availability and mTORC1 activity (Fig 1B). Similar results were also obtained in subcellular fractionation experiments using the INS‐1E β‐cell line, which showed dephosphorylation and nuclear translocation of both TFEB and TFE3 upon nutrient deprivation (Fig 1C). Notably, TFEB nuclear localization in glucose‐starved EndoC‐βH1 cells was completely rescued upon expression of constitutively active forms of the Rag GTPases (Fig 1D), which are known modulators of mTORC1‐mediated TFEB phosphorylation in other cell types (Martina & Puertollano, 2013; Napolitano *et al*, 2020, 2022; Li *et al*, 2022; Cui *et al*, 2023). These data indicate that TFEB and TFE3 are modulated in pancreatic β‐cells via nutrient‐ and Rag GTPase‐dependent activation of mTORC1.

### TFEB and TFE3 control insulin gene expression in β‐cells

**Figure 2:**
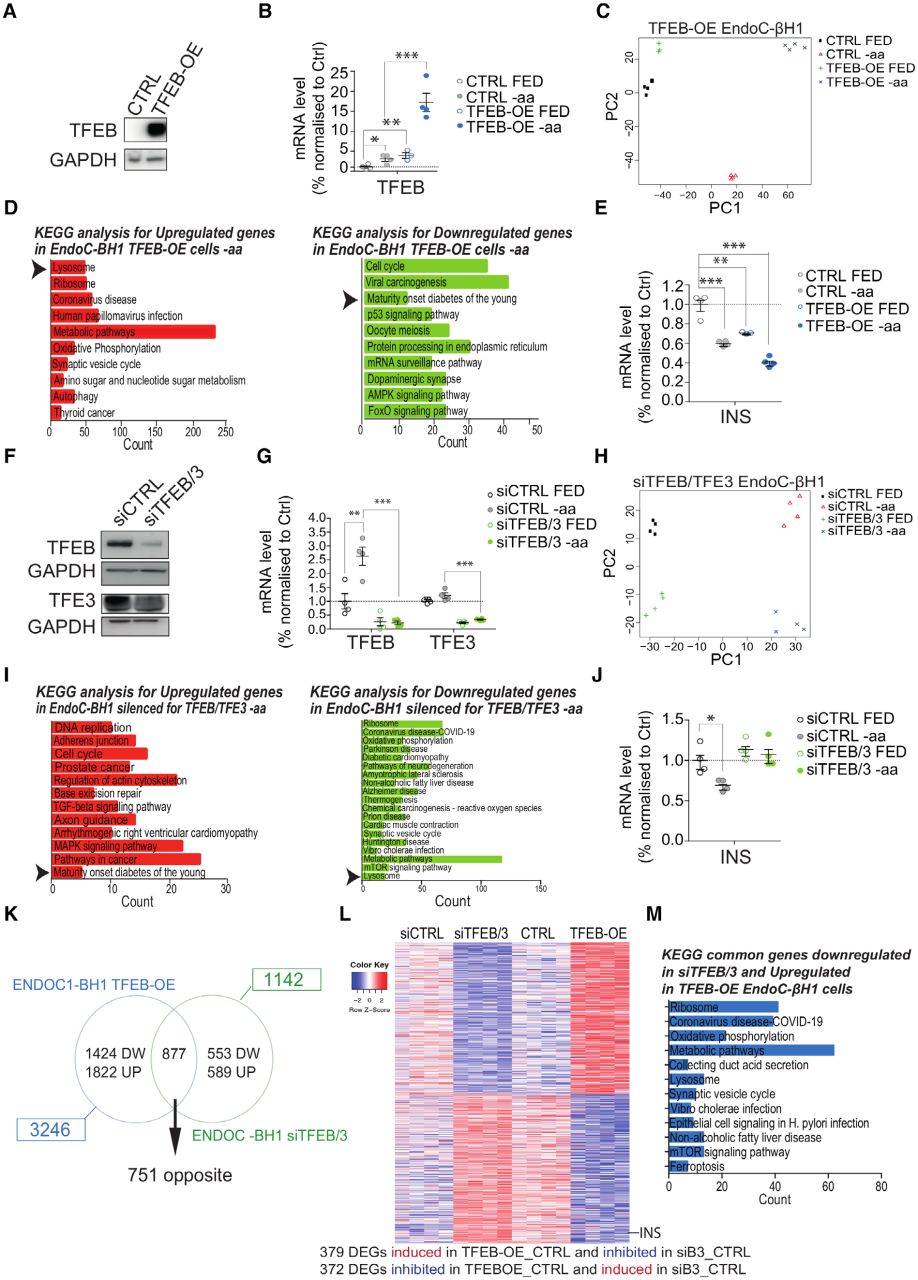
TFEB controls insulin mRNA levels in EndoC‐BH1 cells ARepresentative immunoblot using lysates from cells infected with control lentivirus (CTRL) of TFEB‐V5‐expressing lentivirus (TFEB‐OE) and analyzed using a V5 antibody. GAPDH was used as a loading control.BTFEB mRNA levels in TFEB‐OE and CTRL EndoC‐βH1 cells incubated with full medium (FED) or starved of amino acids (−aa) for 16 h. Data are represented as mean ± standard error (*n* = 3–4/group). Student's two‐tailed *t*‐test: *P‐value < 0.05; **P‐value < 0.01; ***P‐value < 0.001.CPrincipal component analysis plot of transcriptomic data from TFEB‐OE EndoC‐βH1 cells.DGene ontology analysis for significantly upregulated (red) and downregulated (green) genes in TFEB‐OE cells compared to control upon amino acid starvation (−aa) for 16 h. Statistically significant hits (FDR < 0.05) are ranked from the most to the least significant.EINS mRNA levels from control (CTRL) and TFEB‐overexpressing (TFEB‐OE) EndoC‐βH1 cells incubated with full medium (FED) or starved of amino acids (−aa) for 16 h. Data are represented as mean ± standard error (*n* = 3–4/group). Student's two‐tailed *t*‐test: **P‐value < 0.01; ***P‐value < 0.001.FRepresentative immunoblot for TFEB and TFE3 in EndoC‐βH1 cells treated with scramble siRNA (siCTRL) or TFEB‐ and TFE3‐targeting siRNA (siTFEB/3). GAPDH was used as a loading control.GTFEB and TFE3 mRNA levels in siTFEB/3 and control EndoC‐βH1 cells incubated with full medium (FED) or starved of amino acids (−aa) for 16 h. Data are represented as mean ± standard error (*n* = 4/group). Student's two‐tailed *t*‐test: **P‐value < 0.01; ***P‐value < 0.001.HPrincipal component analysis plot of transcriptomic data from siTFEB/TFE3 EndoC‐βH1 cells.IGene ontology analysis for significantly upregulated (red) and downregulated (green) genes in cells depleted for TFEB and TFE3 compared to control upon amino acid starvation (−aa) for 16 h.JINS mRNA levels from EndoC‐βH1 cells treated with scramble siRNA (siCTRL) or TFEB and TFE3‐targeting siRNA (siTFEB/3) incubated with full medium (FED) or starved of amino acids (−aa) for 16 h. Data are represented as mean ± standard error (*n* = 4/group). Student's two‐tailed *t*‐test: *P‐value < 0.05.KVenn diagram showing the comparison of the datasets of TFEB‐OE versus TFEB/3‐depleted EndoC‐βH1 cells.LHeatmap showing the 751 DEGs significantly regulated in opposite correlation in TFEB‐OE versus TFEB/3‐depleted EndoC‐βH1 cells. The lane corresponding to the insulin gene (INS) is indicated.MGene ontology analysis for significantly downregulated genes in siTFEB/3 and upregulated in TFEB‐OE cells in opposite correlation. Source data are available online for this figure.

Next, we analyzed the global transcriptional network modulated by TFEB in β‐cells using both gain‐ and loss‐of‐function approaches. We performed RNA sequencing (RNA‐seq) experiments upon overexpression of TFEB (TFEB^OE^), using a lentiviral vector containing a doxycycline‐dependent TFEB‐V5‐expressing cassette, in fed or starved EndoC‐βH1 cells (Fig 2A and B). Principal component analysis (PCA) revealed that our samples were segregated into four main groups, suggesting that both amino acid starvation and TFEB overexpression profoundly remodeled the entire transcriptome (Fig 2C). Consistent with the role of TFEB as a master regulator of lysosomal function (Sardiello *et al*, 2009), gene set enrichment analysis (GSEA) revealed that the “Lysosome” was the most significantly upregulated gene category (Fig 2D and Dataset EV1). Notably, we also found that the “Maturity Onset Diabetes of the Young (MODY)” gene category, which contains genes important for pancreatic β‐cells, including insulin, was among the downregulated enriched gene categories in TFEB‐overexpressing cells (Fig 2D). In line with these results, TFEB overexpression was sufficient to promote a striking reduction in insulin mRNA levels in fully fed cells, which was comparable to the one observed in fasted control cells (Fig 2E).

In parallel to the gain‐of‐function experiments, we performed transcriptome analysis of EndoC‐βH1 cells concomitantly silenced for both TFEB and TFE3 (siTFEB/3), to avoid reciprocal compensation (Brady *et al*, 2018; El‐Houjeiri *et al*, 2019; Pastore *et al*, 2019), and cultured in the presence or absence of amino acids (Fig 2F and G). Also in this case, PCA showed strong segregation of the experimental groups, confirming that TFEB and TFE3 play a key role in modulating the response of gene expression to nutrients in β‐cells (Fig 2H and I and Dataset EV2). Notably, whereas prolonged amino acid starvation reduced insulin mRNA levels in EndoC‐βH1 cells (Fig 2J), as previously reported (Iwashima *et al*, 1994; Boland *et al*, 2018), such decrease was prevented by depletion of TFEB and TFE3, indicating that these transcription factors are important mediators of insulin suppression upon nutrient deprivation (Fig 2J).

**Figure EV1:**
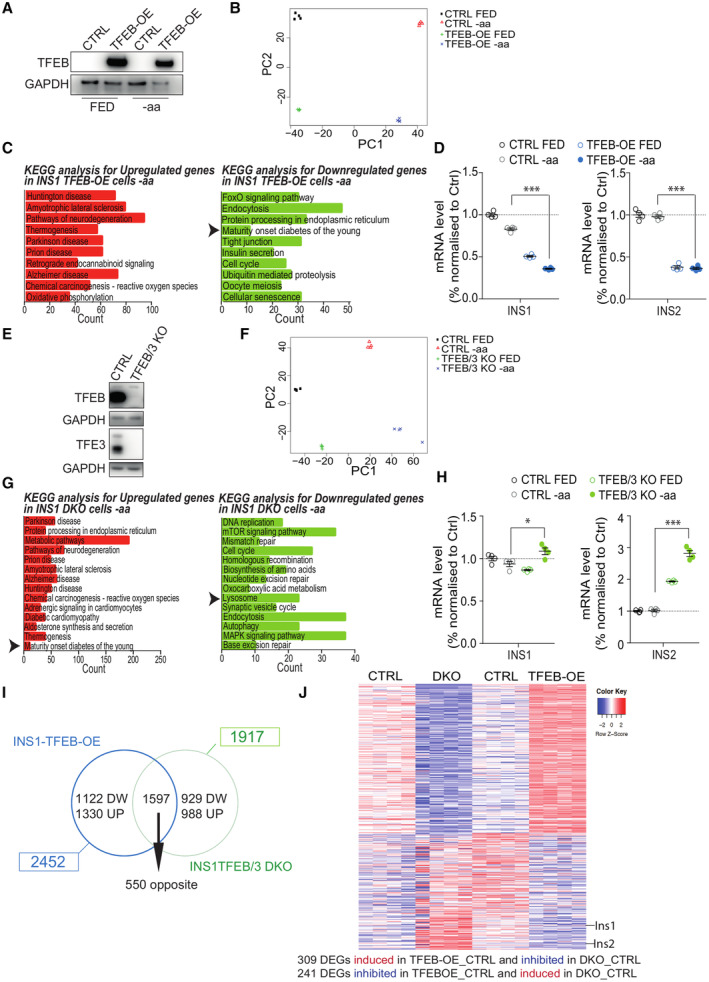
TFEB controls INS mRNA levels in INS‐1E cells ARepresentative immunoblot of lysates from INS‐1E cells infected with hTFEB‐Flag‐expressing lentivirus (TFEB‐OE) or control (CTRL) cells.BPrincipal component analysis plot of transcriptomic data from TFEB‐OE INS1 cells.CGene ontology analysis for significantly upregulated (red) and downregulated (green) genes in TFEB‐overexpressing (TFEB‐OE) cells compared to control (CTRL) upon amino acid starvation (−aa) for 16 h.DINS1 and INS2 mRNA levels from control (CTRL) or TFEB‐overexpressing (TFEB‐OE) INS1 cells incubated with full medium (FED) or upon amino acid starvation (−aa) for 16 h. Each dot represents one mouse (*n* = 3‐4/group). Data are represented as mean ± standard error. Student's two‐tailed *t*‐test: ***P‐value < 0.001.ERepresentative immunoblot for TFEB and TFE3 in DKO cells. GAPDH was used as a loading control.FPrincipal component analysis plot of transcriptomic data from TFEB/TFE3 DKO INS1 cells.GGene enrichment analysis for significantly upregulated (green) and downregulated (red) genes from RNA seq data of DKO cells in comparison to control cells upon amino acid starvation (−aa) for 16 h.HINS1 and INS2 mRNA levels from CTRL or TFEB/TFE3‐DKO INS1 cells incubated with full medium (FED) or upon amino acid starvation (−aa) for 16 h. Each dot represents one mouse (*n* = 3‐4/group). Data are represented as mean ± standard error Student's two‐tailed *t*‐test: *P‐value < 0.05; ***P‐value < 0.001.IVenn diagram showing the comparison of the datasets of TFEB‐overexpressing (TFEB‐OE) versus TFEB/3 DKO INS1 cells.JHeatmap showing the 550 DEGs significantly regulated in opposite correlation in TFEB‐OE versus TFEB/3 DKO INS1 cells. The lanes corresponding to insulin genes (Ins1 and Ins2) are indicated.

Similar results were also obtained by performing transcriptome analysis upon TFEB overexpression in INS‐1E cells (Fig EV1A–C and Dataset EV3), another relevant pancreatic β‐cell line. Importantly, “MODY” was among the downregulated categories in TFEB^OE^ cells compared to control cells (Fig EV1C). Accordingly, TFEB^OE^ caused a strong downregulation of both INS1 and INS2 genes (Fig EV1D). We also performed transcriptome analysis of rat INS‐1E cells depleted for both TFEB and TFE3 (TFEB/TFE3‐DKO cells), generated via the CRISPR/Cas9 system (Fig EV1E–G and Dataset EV4) and found significant upregulation of both *INS1* and *INS2* genes (Fig EV1H), consistent with the results generated in EndoC‐βH1 cells.

Comparison analysis of DEGs that are common to TFEB^OE^ and TFEB/TFE3 depletion upon nutrient deprivation in EndoC‐βH1 (Fig 2K and L and Dataset EV5 and EV6) and INS‐1E (Fig EV1I and J and Dataset EV7 and EV8) cells revealed the presence of 751 and 550 oppositely correlated genes, respectively. GSEA analysis of these genes revealed that “Lysosome” was among the most upregulated gene category in TFEB^OE^ and the most significantly downregulated in TFEB/TFE3‐depleted cells, as expected (Fig 2M and Dataset EV9). Notably, insulin was among the top downregulated in TFEB^OE^ and top upregulated genes in TFEB/TFE3‐depleted cells (Fig 2L).

Overall, these results suggest that TFEB and TFE3 play an important role in the transcriptional regulation of insulin.

### TFEB binds to super‐enhancer regions responsible for modulation of insulin transcription

**Figure 3:**
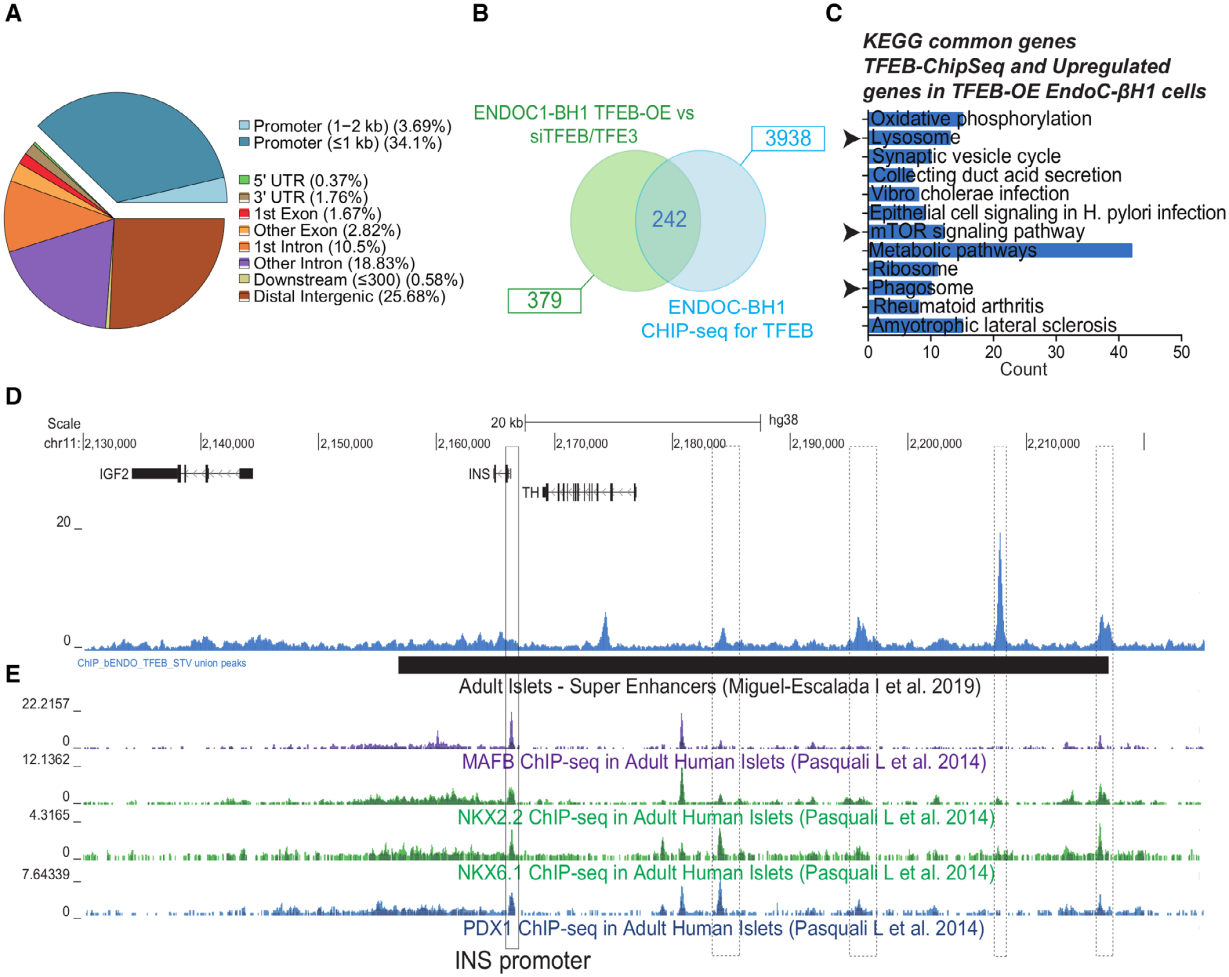
ChIP‐seq analysis identifies TFEB‐binding sites in insulin super‐enhancer AGraphic representation of the different genomic sites of TFEB binding in EndoC‐βH1 cells upon 16 h amino acid starvation.BVenn diagram of genes upregulated upon TFEB overexpression and downregulated in siTFEB/3 and TFEB‐binding sites located inside proximal promoters (−1,000 + 300).CGene ontology analysis for TFEB promoter‐bound direct targets.D, EAlignment of the TFEB ChIP‐seq track, aligned with ChIP‐seq tracks of other known INS regulators (MAFB, NKX2.2, NKX6.1, and PDX1), at the INS locus. Super‐enhancer, black box.

**Figure EV2:**
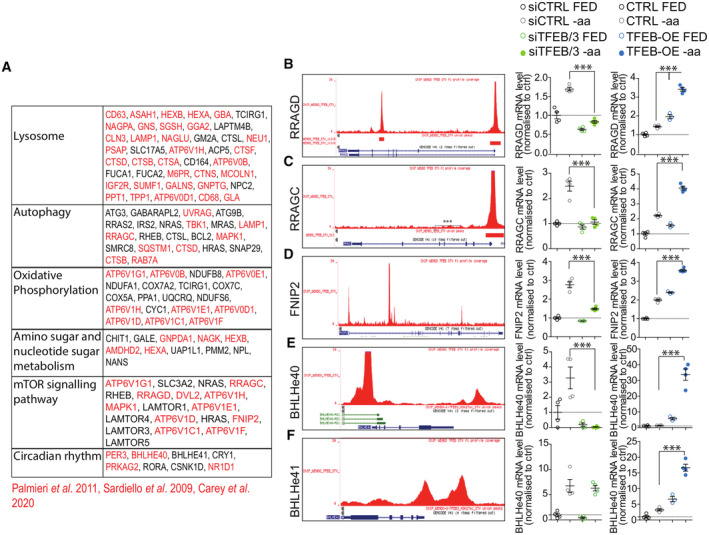
ChIP‐Seq analysis identifies direct TFEB targets in pancreatic beta cells ATop genes whose promoters are bound by TFEB upon fasting and upregulated upon TFEB upregulation in EndoC‐BH1 cells sorted by gene categories. Genes in red are previously identified TFEB direct targets.B–FOn the left, alignment of the TFEB ChIP‐seq track at the indicated gene loci. On the right, mRNA levels of the indicated genes from EndoC‐βH1 cells treated with scramble siRNA (siCTRL) or TFEB‐ and TFE3‐targeting siRNA (siTFEB/3) or TFEB‐OE compared to control EndoC‐βH1 cells incubated with full medium (FED) or upon amino acid starva tion (−aa) for 16 h. Data are represented as mean ± standard error. Each dot represents an independent experiment, ***P < 0.001; Student's two‐tailed unpaired *t‐*test.

TFEB has been known to act as a positive regulator of transcription by binding to proximal promoters (Sardiello & Ballabio, 2009; Palmieri *et al*, 2011). Thus, the evidence that TFEB in β‐cells can work as a negative transcriptional regulator of insulin was surprising and prompted us to determine whether TFEB exerts this function directly or indirectly. To this end, we performed ChIP‐seq analysis for endogenous TFEB in EndoC‐βH1 upon amino acid starvation. We found that TFEB binds to the proximal promoter regions (i.e., < 300 bp from the transcriptional start site) of 4,765 genes in β‐cells. Consistent with previous results obtained in other cell types and tissues (Sardiello *et al*, 2009; Palmieri *et al*, 2011; Brooks & Dang, 2019; Pastore *et al*, 2019; Gambardella *et al*, 2020), the comparison of genes upregulated upon TFEB gain of function (logFC>1) and downregulated upon TFEB/TFE3 depletion with genes containing at least one TFEB binding site in their proximal promoter (Fig 3A and B) revealed an enrichment of known TFEB‐regulated gene categories, including “Lysosome,” “Metabolic pathways,” and “circadian rhythm” (Figs 3C and EV2 and Dataset EV10). However, comparison of genes that were downregulated in our RNA‐seq data upon TFEB overexpression with our ChIP‐seq data failed to reveal TFEB binding sites in insulin promoter (Fig 3D) despite the evidence that the insulin gene was under TFEB transcriptional control. These data suggested that TFEB effect on INS transcription was not caused by direct promoter binding. Of note, our ChIP‐seq data revealed that only approximately 40% of TFEB binding sites were in promoter regions (< 2 kb from the TSS) in pancreatic β‐cells (Fig 3A). Interestingly, we found that TFEB binds to conserved super‐enhancer (SE) regions previously identified both in the EndoC‐βH1 cell line and in β‐cells obtained from human islets and hESC (Mularoni *et al*, 2017; Lawlor *et al*, 2019). By comparing our ChIP‐seq data with available ChIP‐seq and ChiA‐Pet data collected on the *Islet Regulome browser* (http://pasqualilab.upf.edu/app/isletregulome) and the *Shiny App for Visualizing EndoC‐*β*H1 and Human Islet Genomics Data* (https://shinyapps.jax.org/endoc‐islet‐multi‐omics/) databases (Mularoni *et al*, 2017, Lawlor *et al*, 2019), we found that TFEB binds in at least four different sites of a super‐enhancer region in the close vicinity of the *INS* locus in EndoC‐βH1 cells (Fig 3D). Interestingly, two out of the four sites are specifically bound by TFEB, as no other well‐known *INS* transcriptional modulator, such as MAFB, NKX2‐2, NKX6‐1, and PDX1, has been reported to bind these sites in other ChIP‐Seq experiments thus far (Fig 3E). These data suggest a possible mechanism by which TFEB suppresses insulin gene transcription.

### TFEB and TFE3 control insulin production and glucose homeostasis *in vivo*

**Figure 4:**
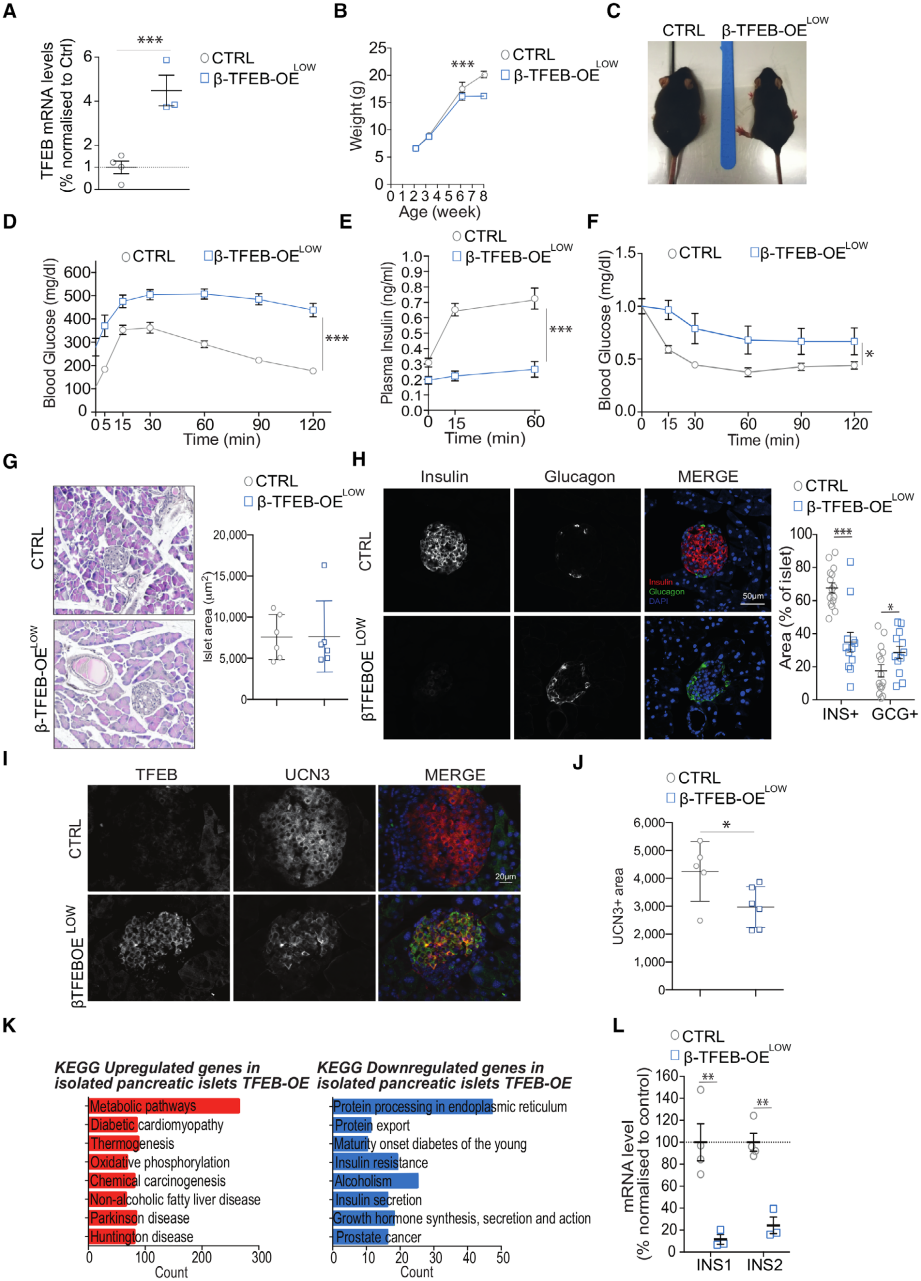
Beta‐cell‐specific TFEB gain‐ or TFEB/TFE3 loss‐of‐function *in vivo* leads to impairment of insulin production and glucose homeostasis AmRNA levels of TFEB in isolated islets from control and βTFEBOE^LOW^ mice. Each dot represents one mouse (*n* = 3–4/group). Data are represented as mean ± standard error. Student's two‐tailed *t*‐test: ***P‐value < 0.001.BWeight gain of control (*n* = 20) and βTFEBOE^LOW^ mice (*n* = 14). Data are represented as mean ± standard error. Two‐way ANOVA: ***P‐value < 0.001.CRepresentative images of control and βTFEBOE^LOW^ mice showing different body sizes.DGlucose tolerance test (GTT) of control (*n* = 15) and βTFEBOE^LOW^ (*n* = 11) mice. Data are represented as mean ± standard error. Two‐way ANOVA: ***P‐value < 0.001.EGlucose‐stimulated insulin secretion (GSIS) for control (*n* = 9) and βTFEBOE^LOW^ (*n* = 7) mice. Data are represented as mean ± standard error. Two‐way ANOVA: ***P‐value < 0.001.FInsulin tolerance test (ITT) normalized to T0 in control and βTFEBOE^LOW^ mice (*n* = 9/group). Data are represented as mean ± standard error. Two‐way ANOVA: *P‐value < 0.05.GRepresentative images of pancreas slides from control and βTFEBOE^LOW^ mice stained with hematoxylin/eosin and quantification of islet area (*n* = 6/group; *n* = 5–10 islets per mouse). Data are represented as mean ± standard error.HRepresentative immunofluorescence picture using insulin and glucagon antibodies on fixed pancreas of control and βTFEBOE^LOW^ mice (scale bar 50 μm). The graph shows the quantification of the relative insulin (INS^+^)‐ and glucagon (GCG^+^)‐positive area from islets of control and βTFEBOE^LOW^ mice. Each dot represents an islet (*n* = 15 for CTRL and *n* = 12 for βTFEBOE^LOW^). Data are represented as mean ± standard error. Student's two‐tailed *t*‐test: ***P‐value < 0.001.I, JRepresentative immunofluorescence images for TFEB and UCN3 (I) on fixed pancreas of control and βTFEBOE^LOW^ mice (scale bar 20 μm) and quantification of the UCN3^+^ area per islet in control and βTFEBOE^LOW^ pancreas (*n* = 3/group; *n* = 3–14 islets per mouse) (J). Data are represented as mean ± standard error. Student's two‐tailed *t*‐test: *P‐value < 0.05.KGene ontology analysis of RNA‐seq data of isolated islets from control and βTFEBOE^LOW^ mice.LmRNA levels of indicated genes assessed on isolated islets from control and βTFEBOE^LOW^ mice. Each dot represents one mouse (*n* = 3–4/group). Data are represented as mean ± standard error. Student's two‐tailed *t*‐test: **P‐value < 0.01. Source data are available online for this figure.

To assess the physiological relevance of TFEB on INS transcription and β‐cell function, we performed *in vivo* gain‐of‐function experiments by generating mouse models in which TFEB was specifically overexpressed in β‐cells. This was achieved by breeding INS1‐CRE mice (Thorens *et al*, 2015) with two different conditional TFEB overexpressing mouse lines (TFEBOE^LOW^ and TFEBOE^HIGH^), previously generated in our laboratory (Settembre *et al*, 2013), leading to the generation of the β‐TFEBOE^LOW^ and β‐TFEBOE^HIGH^ mouse lines in which TFEB was overexpressed specifically in β‐cells at low and high levels, respectively. β‐TFEBOE^LOW^ mice showed a ~ 4‐fold increase in TFEB mRNA levels in pancreatic β‐cells compared to controls (Fig 4A), which was associated with smaller size and lower weight compared to control littermates (Fig 4B and C). Strikingly, these mice also showed profound glucose intolerance (GTT) (Fig 4D), impaired glucose‐stimulated insulin secretion (GSIS) (Fig 4E), and insulin resistance (Fig 4F). Hematoxylin/Eosin (HE) staining on fixed pancreas revealed the presence of numerous regular‐size islets in both β‐TFEBOE^LOW^ and control mice (Fig 4G). However, immunofluorescence analysis using insulin and glucagon antibodies showed a marked reduction in insulin signal in β‐cells of β‐TFEBOE^LOW^ compared to control mice, with a slightly increased glucagon staining in α‐cells in the islet periphery (Fig 4H). Importantly, despite the reduced levels of insulin expression in β‐cells observed in β‐TFEBOE^LOW^ islets, immunostaining analysis using an antibody recognizing UCN3, an established marker of mature β‐cells (van der Meulen *et al*, 2012), revealed no effects of TFEB overexpression on β‐cell differentiation (Fig 4I), thus suggesting that β‐cells produce less insulin despite having a normal islet architecture. Consistent with increased number of α‐cells, TFEB overexpression resulted in a slight but significant reduction in β‐cell area quantified as UCN3^+^ staining, indicating that β‐cell mass is reduced in β‐TFEBOE^LOW^ mice (Fig 4J) likely as a secondary effect of the diabetic phenotype. Together, these results indicate that TFEB plays an important physiological role in β‐cell function. Accordingly, and in line with the results obtained in cell lines, RNA‐seq analysis of islets isolated from β‐TFEBOE^LOW^ mice showed severe downregulation of the “MODY” gene category (Fig 4K and Dataset EV11). In addition, mRNA levels of *INS1* and *INS2* were strongly downregulated in β‐TFEBOE^LOW^ mice compared to controls (Fig 4L).

**Figure EV3:**
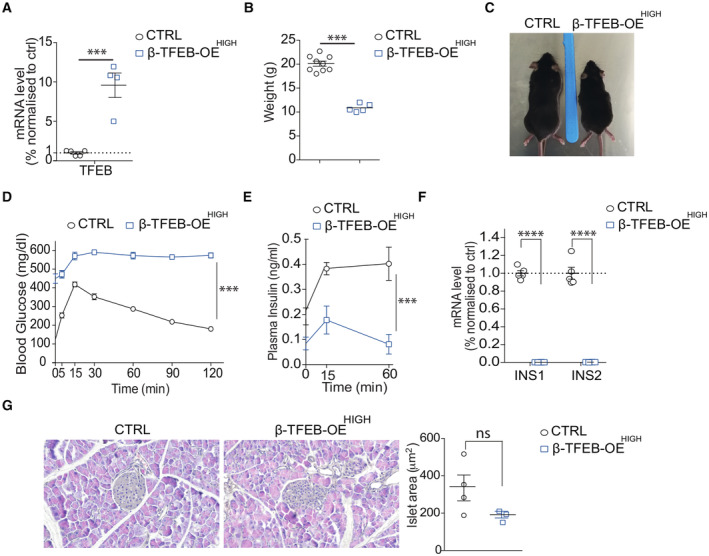
TFEB upregulation in pancreatic β‐cells *in vivo* results in suppression of insulin transcription and glucose intolerance AmRNA levels of TFEB in isolated islets from control and βTFEBOE^HIGH^ mice. Each dot represents a mouse (*n* = 4‐5/group). Data are represented as mean ± standard error. Student's two‐tailed *t*‐test: ***P‐value < 0.001.BWeight of control and βTFEBOE^HIGH^ mice. Each dot represents a mouse (*n* = 9 for CTRL and *n* = 5 for βTFEBOE^HIGH^ mice). Data are represented as mean ± standard error. Student's two‐tailed *t*‐test: ***P‐value < 0.001.CRepresentative images of control and βTFEBOE^HIGH^ mice showing different body sizes.DGlucose tolerance test (GTT) of control (*n* = 12) and βTFEBOE^HIGH^ (*n* = 7) mice. Data are represented as mean ± standard error. Two‐way ANOVA: ***P‐value < 0.001.EGlucose‐stimulated insulin secretion (GSIS) for control (*n* = 11) and βTFEBOE^HIGH^ (*n* = 7) mice. Data are represented as mean ± standard error. Two‐way ANOVA: ***P‐value < 0.001.FmRNA levels of Ins1 and Ins2 in isolated islets from control and βTFEBOE^HIGH^ mice. Each dot represents a mouse (*n* = 5/group). Data are represented as mean ± standard error. Student's two‐tailed *t*‐test: ****P‐value < 0.0001.GRepresentative images of pancreas slides from control and βTFEBOE^HIGH^ mice, stained with hematoxylin/eosin and relative quantification of islet area (*n* = 3‐4/group). Data are represented as mean ± standard error.

Analysis of β‐TFEBOE^HIGH^ mice showed higher levels of TFEB compared to β‐TFEBOE^LOW^ mice (Fig EV3A), as expected. This resulted in a similar but more severe phenotype characterized by heavily impaired growth (Fig EV3B and C), greater glucose intolerance, and impaired insulin secretion (Fig EV3D and E), as well as complete absence of *INS1*/*INS2* expression (Fig EV3F). As in β‐TFEBOE^LOW^ mice, islet area of β‐TFEBOE^HIGH^ mice was indistinguishable from controls (Fig EV3G and H).

**Figure 5:**
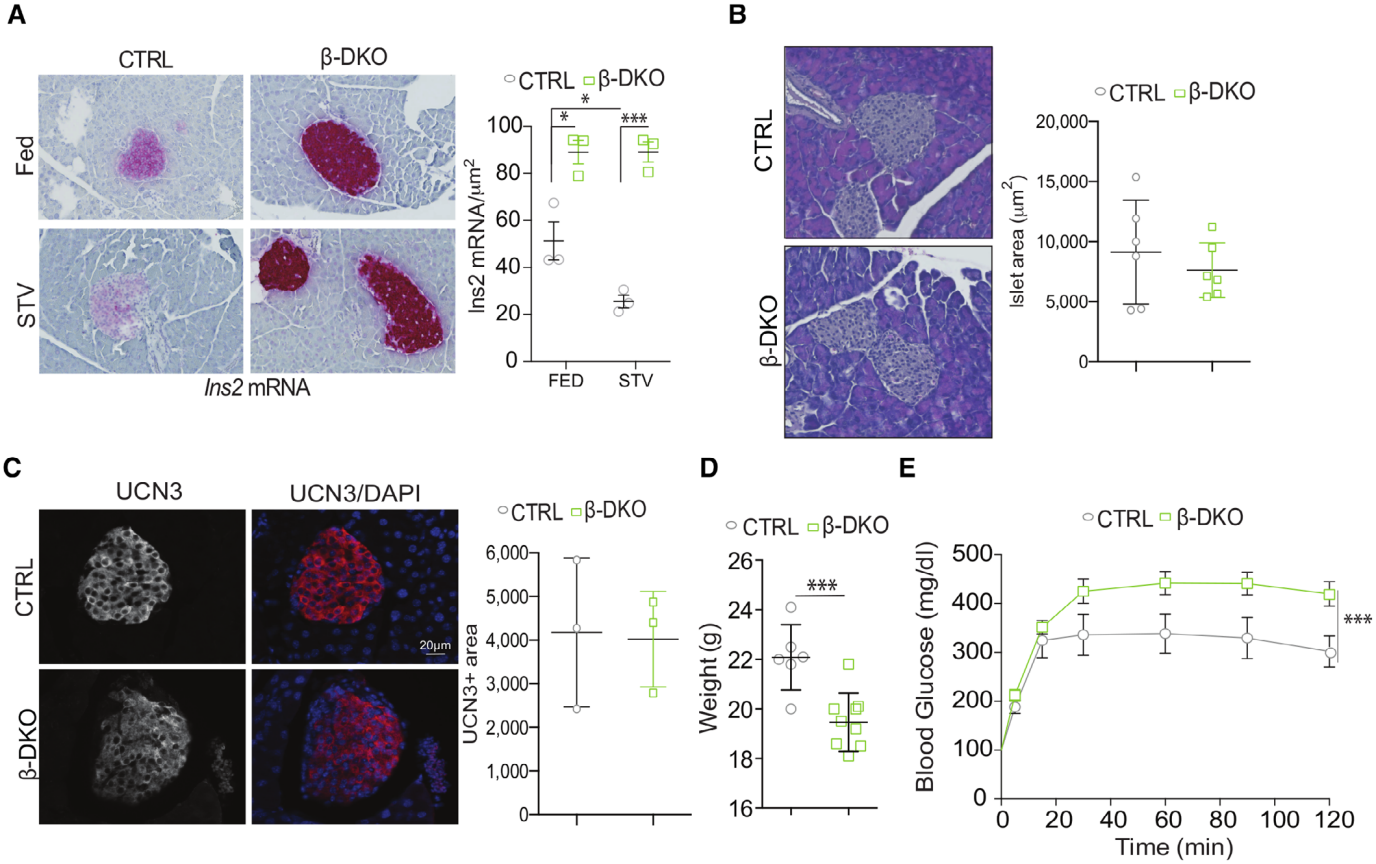
Beta‐cell‐specific deletion of TFEB and TFE3 results in enhanced insulin transcription and impaired glucose homeostasis ARepresentative images of RNA scope analysis for *Ins2* mRNA in pancreas from control and β‐DKO mice in fed and starved conditions with relative quantification (*n* = 3/group). Each dot represents one mouse. Data are represented as mean ± standard error. Student's two‐tailed *t*‐test: *P‐value < 0.05; ***P‐value < 0.001.BRepresentative images of pancreas slides from control and β‐DKO mice stained with hematoxylin/eosin and quantification of islet area (*n* = 6/group; *n* = 11–25 islets per mouse). Each dot represents one mouse.CRepresentative immunofluorescence images for UCN3 and quantification of the UCN3^+^ area per islet in control and β β‐DKO pancreas (*n* = 3/group; *n* = 8–25 islets per mouse). Each dot represents one mouse. Data are represented as mean ± standard error. Student's two‐tailed *t*‐test: ***P‐value < 0.001.DBody weight of control and β‐DKO mice at 8 weeks of age.EGlucose tolerance test (GTT) of control and β‐DKO mice. Each dot represents one mouse (*n* = 8/group). Two‐way ANOVA: ***P‐value < 0.001. Source data are available online for this figure.

Finally, we generated TFEB β‐cell‐specific/TFE3 double‐KO mice (β‐DKO mice) by crossing INS1‐CRE mice with TFEB^fl/fl^ (Settembre *et al*, 2012) and TFE3 full KO mice (Steingrimsson *et al*, 2002). Consistent with *in vitro* data, β‐DKO mice failed to suppress *Ins2* mRNA upon overnight starvation and showed elevated *Ins2* levels upon refeeding, as demonstrated by RNA scope analysis in pancreatic sections (Fig 5A). No differences were detected in islet area (Fig 5B), β‐cell mass, and β‐cell maturation, as demonstrated by UCN3 staining (Fig 5C). Despite the striking increase in *Ins2* mRNA, β‐DKO mice were characterized by lower body weight (Fig 5D) and glucose intolerance (Fig 5E) compared to control littermates, likely as a result of defective insulin release and consistent with recently published data (Park *et al*, 2022). Together these data suggest that TFEB and TFE3 act, at multiple levels, as crucial mediators of pancreatic β‐cell function and metabolic homeostasis.

## Discussion

Considerable work in the past years has tremendously advanced our knowledge of the mechanisms regulating insulin granule maturation and secretion as an acute response to increased nutrient levels. Previous studies have shown that mTORC1 activity is crucial for maintaining pancreatic β‐cell homeostasis by modulating insulin mRNA translation and β‐cell mass (Rachdi *et al*, 2008; Mori *et al*, 2009; Blandino‐Rosano *et al*, 2017). However, the mechanisms governing insulin transcriptional regulation in response to variations in nutrient availability have remained less understood. In the present manuscript, we define a nutrient‐controlled transcriptional mechanism that regulates insulin production to fine‐tune glucose homeostasis at both cellular and organismal levels. Over the years, TFEB and TFE3 have emerged as important metabolic regulators in the liver and muscles, impacting body weight and glucose homeostasis (Settembre *et al*, 2013; Mansueto *et al*, 2017; Pastore *et al*, 2017). Here, we identify TFEB and TFE3 as key modulators of pancreatic β‐cell function and further highlight the importance of these transcription factors in overall organismal metabolic regulation. As previously described in other cell types, TFEB and TFE3 are activated under scarce nutrient conditions and mTORC1 inhibition. In β‐cells, fasting is associated with insulin downregulation (Rabinovitch *et al*, 1976; Zawalich *et al*, 1979; Giddings *et al*, 1981; Iwashima *et al*, 1994; Boland *et al*, 2018) to prevent undesired insulin secretion and further glycemic decrease in a hypoglycemic state. Our data show that TFEB and TFE3 mediate this process by suppressing insulin transcription and thus controlling glucose homeostasis. Accordingly, *INS* gene expression is severely suppressed both *in vitro* and *in vivo* upon acute and constitutive TFEB overexpression, respectively. Moreover, the physiological suppression of insulin transcription that occurs upon nutrient starvation in β‐cells is severely impaired in TFEB/TFE3‐depleted cells and mice. Although our *in vitro* studies were mainly focused on TFEB/TFE3‐mediated control of insulin gene expression during amino acid starvation, we also observed that glucose deprivation promotes TFEB nuclear translocation in pancreatic beta cells, thus indicating that TFEB and TFE3 are main physiological regulators of insulin transcription in response to variations in the availability of different nutrients. Our data also suggest that mTORC1 activity in β‐cells controls both acute and sustained responses to circulating nutrients, by regulating not only insulin mRNA translation, as previously shown (Blandino‐Rosano *et al*, 2017), but also by controlling INS transcription via TFEB and TFE3 transcription factors. Surprisingly, however, TFEB/TFE3‐deficient mice, despite showing a massive increase in INS mRNA levels, do not show hypoglycemia but instead are glucose intolerant, suggesting that lack of TFEB and TFE3 may affect β‐cell function and insulin production at different levels.

Our findings also highlight a potential function of these transcription factors as negative transcriptional modulators. The evidence shown here that TFEB binds to enhancer cluster regions flanking the *INS* locus, previously proposed to act as a super‐enhancer (Miguel‐Escalada *et al*, 2019), suggests that TFEB and TFE3 may directly suppress *INS* transcription via super‐enhancer binding. Interestingly, a previously reported motif‐based binding prediction analysis identified TFEB as part of a core regulatory circuit (CRC) controlling β‐cell function via super‐enhancer binding (Alvarez‐Dominguez *et al*, 2020). However, at this stage, we cannot exclude that TFEB and TFE3 instead repress the transcription of insulin and other β‐specific genes in an indirect manner. For instance, consistent with a previous study (Carey *et al*, 2020), we found that TFEB promotes the transcription of *BHLHE40* and *BHLHE41* (Fig EV2E), two important transcriptional repressors which have been recently shown to suppress insulin gene expression via transcriptional inhibition of *INS* master regulators PDX1 and MafA (Tsuyama *et al*, 2023). Interestingly, TFEB, BHLHE40, and BHLHE41 have been shown to play a role in circadian rhythm regulation (Honma *et al*, 2002; Kato *et al*, 2014; Brooks & Dang, 2019; Pastore *et al*, 2019) which is central for β‐cell function (Marcheva *et al*, 2010; Perelis *et al*, 2015; Alvarez‐Dominguez *et al*, 2020). Whether TFEB, BHLHE40, and BHLHE41 are part of a transcriptional cascade that results in insulin gene regulation remains to be established in future studies.

Dysregulation of glucose homeostasis and β‐cell dysfunction is a hallmark of human diseases, such as diabetes. Our study supports the key role of TFEB and TFE3 in pancreatic β‐cell regulation and uncovers a new mechanism by which these transcription factors modulate organismal metabolism. These data may have important clinical implications in diabetes and other metabolic diseases.

## Materials and Methods

### Materials

*Reagents used in this study were obtained from the following sources*: antibodies to phospho‐p70 S6 kinase (Thr389) (1A5) (Cat# 9206), p70 S6 kinase (Cat# 9202), Rheb1 (Cat# 13789), 4E‐BP1 (Cat# 9644), phospho‐4E‐BP1 (Ser65) (Cat# 9456), human TFEB (Cat# 4240), and insulin (Cat# 4590) were from Cell Signaling Technology; antibodies to GAPDH (6C5) (Cat# sc‐32233) and LAMP1 (Cat# Sc20011) were from Santa Cruz; antibodies to TFE3 (Cat# HPA023881), glucagon (Cat#G2654), and V5 (Cat # V8137) were from Sigma Aldrich; antibody to TFEB (Cat# A303‐673A) was from Bethyl; antibody to HA.11 Epitope Tag (Cat# 901513) was from Biolegend; and the antibody for UCN3 was from Phoenic Pharmaceutical (Cat#H‐019029).

*Plasmids*: pRK5‐HA GST RagA‐Q66L (#19300) and pRK5‐HA GST RagC‐S75L (#19305) were a kind gift from David Sabatini (Addgene plasmids).

*Chemicals*: Torin 1 (Cat# 4247) was from Tocris; Protease Inhibitor Cocktail (Cat# P8340) was from Sigma Aldrich; and PhosSTOP phosphatase inhibitor cocktail tablets (Cat# 04906837001) were from Roche.

### Cell culture

Human EndoC‐βH1 cells were obtained from Univercell® company and maintained following the company instructions in a DMEM‐derived medium. INS‐1E cells were purchased from AddexBio (C0018009) and grown in RPMI 1640 supplemented with 10% FCS, 10 mM Hepes, 2 mM glutamine, 0.05 mM β‐mercapto‐ethanol, 1 mM PyrNa, and 1× penicillin–streptomycin solution. For experiments involving amino acid starvation, cells were rinsed twice with PBS and incubated in amino acid‐free RPMI (Cat# R9010‐01, USBiological) supplemented with 10% dialyzed FBS. Where indicated, cells were re‐stimulated with 1× water‐solubilized mix of essential (Cat#11130036, Thermo Fisher Scientific) and non‐essential (Cat# 11140035, Thermo Fisher Scientific) amino acids re‐suspended in amino acid‐free RPMI. For glucose starvation, cells were rinsed twice with PBS and incubated with cell culture medium prepared in a DMEM w/o glucose (Cat# 11966025, Thermo Fisher Scientific). Glucose re‐feeding was obtained by replacing glucose starvation medium with full‐nutrient cell culture medium. For silencing experiments, cells were transfected using the RNAi Max® reagent from Invitrogen following the kit instructions and incubated for 72 h. The following siRNAs were used in this study: ON‐Target plus® SMART pool Human TFE3 #L‐009363‐00‐0020, ON‐Target plus® SMART pool Human TFEB #L‐009798‐00‐0020, and ON‐Target plus® SMART pool Non‐targeting control #D‐001810‐10‐20.

### CRISPR Cas9 gene editing of the INS‐1E cells

For KO clone generation in INS‐1E cells, wild‐type cells were transfected with the Sigma CRISPr plasmids carrying simultaneously the gRNA and Cas9 (TFEB gRNA: GCTGCCATGGCGTCGCGCATCGG, TFE3 gRNA: CCGGCGAGCTTGCTGCAAG).

Single clones were screened for desired editing by sequencing (TFEB sequencing primers: FW 5′‐ACTGAAGGACAGAGTCTTCACC, RV 5′‐GACAGAAGAGGCAGAGGCCTTA; TFE3 sequencing primers: FW 5′‐TGTGCCCCAGGTGTTTATGG, RV 5′‐GCTACGGCCTCTTACCTCCT) and immunoblot. At least two clones per condition were tested in parallel for control and edited cell lines.

### Viral infection

Retroviral plasmids encoding TFEB‐3X‐FLAG (Settembre *et al*, 2012) were co‐transfected with pCMVgag/pol and CMV VSV‐G packaging plasmids into actively growing HEK‐293T cells using FuGENE 6 transfection reagent. Virus‐containing supernatants were collected 48 h after transfection, diluted 1:2, and used to infect one six‐well plate of INS1‐E cells in presence of 8 μg/ml polybrene. Cells were analyzed 3 days after infection.

EndoC‐BH1 were infected with lentivirus for TFEB‐V5 and control luciferase expression using the Lenti‐X™ Tet‐One™ Inducible Expression System (Puro) from Takara.

### High‐content analysis of TFEB subcellular localization

EndoC‐βH1 cells were seeded in 96‐well plates and incubated for 24 h. Cells were then treated as indicated, rinsed with PBS once, fixed for 10 min with 4% paraformaldehyde, and stained with TFEB antibodies (Cat# 4240, Cell signaling) and DAPI. For the acquisition of the images, at least 10 fields were acquired per well of the 96‐well plate by using confocal automated microscopy (Opera High Content System; Perkin‐Elmer). A dedicated script (Medina *et al*, 2015) was used for analysis of TFEB localization on the different images (Harmony and Acapella software; Perkin‐Elmer). The script calculates the ratio value resulting from the average intensity of nuclear TFEB fluorescence divided by the average of the cytosolic intensity of TFEB fluorescence. *P*‐values were calculated on the basis of mean values from independent wells.

### RNA extraction and RT–PCR

RNA extraction was performed using the Qiagen RNeasy® kit, following the kit instructions.

cDNA was generated from extracted RNA using the Qiagen QuantiTect Reverse Transcription Kit following the kit instructions. RT–PCR was done using the LightCycler® 480 SYBR Green I Master following the kit instructions using the LightCycler 480 II detection system (Roche). For expression studies, the qRT–PCR results were normalized against an internal control (HPRT). Primers used are listed in Table EV1.

### 3′mRNA sequencing library preparation, data processing, and analysis

Total RNA was quantified using the Qubit 2.0 fluorimetric Assay (Thermo Fisher Scientific). For RNA‐seq analysis, library preparation was performed with a total of 100 ng of RNA from each sample using QuantSeq 3′mRNA‐Seq Library prep kit (Lexogen) according to manufacturer's instructions. Amplified fragmented cDNA of 300 bp in size was sequenced in single‐end mode by NovaSeq 6000 (Illumina) with a read length of 100 bp. Illumina NovaSeq 6000 base call (BCL) files were converted into fastq file through bcl2fastq. Sequence reads were trimmed BBDuk (sourceforge.net/projects/bbmap/) to remove adapter sequences and low‐quality end bases (Q < 20). Alignment was performed with STAR 2.6.0a (Dobin *et al*, 2013) on the Hg38 reference provided by UCSC Genome Browser (Lee *et al*, 2020). Alignment to mm10, and rn6 reference genome assembly (Dobin *et al*, 2013), and counting by gene (Anders *et al*, 2015) has been performed by using Ensembl assembly (release 93). Gene expression levels were determined with HTseq‐count 0.9.1 (Anders *et al*, 2015). Differential expression analyses were performed using edgeR (Robinson *et al*, 2010), a statistical package based on generalized linear models, suitable for multifactorial experiments. The threshold for statistical significance chosen was false discovery rate (FDR) < 0.05. Gene ontology (GOEA) and functional annotation clustering analyses were performed on the induced and inhibited differentially expressed genes (DEGs) obtained in each RNA‐sequencing dataset by using DAVID Bioinformatic Resources (Huang da *et al*, 2009a,b) restricting the output to Biological Process terms (BP_FAT). The “Kyoto Encyclopedia of Genes and Genomes” (KEGG Pathway) analyses were also performed (Kanehisa & Goto, 2000; Kanehisa *et al*, 2012). The threshold for statistical significance of GOEA was FDR < 0.1 and enrichment score ≥ 1.5, while for the KEGG pathway analyses was FDR < 0.1.

Heatmap and Venn diagrams were generated using custom annotation scripts.

List of abbreviations: FDR, false discovery rate; DEGs, differentially expressed genes.

### Immunoblot assay

Protein lysates were extracted in RIPA buffer (Thermo Fisher) supplemented with the phosphatase inhibitor PhosSTOP *EASYpack* (Roche #04906837001) and the protease inhibitor cocktail (Sigma #P8340). Lysates were centrifuged for 10 min at 14,000 *g* at 4°C and supernatant conserved. Protein concentration was measured by Bradford assay and 50 μg of protein lysates were loaded for each sample, supplemented with Laemmle buffer. The lysates were run on precast NuPAGE™ 4–12% Bis‐Tris Gel (Invitrogen #NP0335BOX [10 wells] or #NP0336BOX [15 wells]) inside the Xcell SureLock Mini cells and MOPES running buffer. Proteins were wet transferred for 1 h 30 min at 100 V on Immobilon® membrane (#IPVH00010) using the Bio‐Rad® Mini Trans‐ blot® Cell. Membranes were subsequently incubated in 5% skim‐milk TBS‐T blocking solution for 1 h at room temperature. Incubation in the TBS‐T 5% BSA primary antibody‐containing solution was performed overnight at 4°C. After three TBS‐T washes, incubation in the TBS‐T 5% BSA secondary antibody‐containing solution was performed for 1 h at room temperature, followed by three TBS‐T washes. Membranes were developed using the ECL solution from Cyanagen ηC Ultra 2.0 ECL #XLS075L. Pictures were acquired with the GE® Amersham Imager 600, the UVITEC Q9 Mini Alliance, or UVITEC Mini HD9.

### Immunofluorescence assay

PhenoPlate 96 wells (Perkin Elmer) were coated and then seeded with EndoC‐BH1 cells at a density of 25,000 cells/well. Following treatment, cells were fixed in 4% PFA for 10 min then washed with PBS and permeabilized with PBS 0.02% Triton‐X solution for 7 min. A blocking step was then performed using the blocking buffer (PBS, 3% BSA, 0.05% Saponin, 1% horse serum, and 50 mM NH_4_Cl). Following the blocking steps, primary and secondary antibody incubations were performed in blocking buffer solution. Nuclei were stained with DAPI.

### Histological analysis

H&E staining was performed following the IHC World protocol. Bright‐field sections were scanned with ZEISS Axio Scan.Z1.

Immunofluorescence analyses were performed in 6 μm paraffin sections with VENTANA BenchMark Ultra automated staining instrument (Ventana Medical Systems, Roche), using VENTANA reagents except as noted, according to manufacturer's instructions. Slides were deparaffinized using EZ Prep solution (cat # 950‐102) for 16 min at 72°C. Epitope retrieval was accomplished with CC1 solution (cat # 950‐224) at a high temperature (95°C) for a period that is suitable for a specific tissue type. Antibodies were titered with a blocking solution into user‐fillable dispensers for use on the automated stainer. Slides were developed using DISCOVERY FAM Kit (cat # 760‐243) and DISCOVERY Red 610 Kit (cat #760‐245, Roche) for 8 min. Slides were then counterstained with DISCOVERY QD DAPI (cat # 760‐4196) for 8 min. Fluorescent sections were acquired using ZEISS microscope.

### Automated RNA detection (RNA scope)

Insulin mRNA expression was determined using ISH with the fully automated RNA‐scope assay on the VENTANA BenchMark Ultra (Ventana Medical Systems, Roche) platform. Sections were deparaffinized on the instrument, followed by target retrieval (16 min at 97°C) and protease treatment (16 min at 37°C). Probes (RNAscope® 2.5 VS Probe‐ Mm‐Ins2‐O1 #497819, ACD) were then hybridized at 43°C followed by RNAscope amplification (RNAscope® VS Universal AP Reagent Kit #323250, ACD) and red chromogenic detection using VS detection reagents (DISCOVERY mRNA Red Detection Kit #07099037001, Roche). Slides were then counterstained with hematoxylin II (cat # 790‐2208) for 8 min, followed by Bluing reagent (cat # 760‐2037) for 4 min. Bright‐field sections were scanned with ZEISS Axio Scan.Z1. The whole digital slides were viewed by zen blue software. Antibodies used: TFEB (Bethyl A303‐673A). Quantitative analysis of Insulin mRNA transcripts was performed using QuPath software.

### Chromatin immunoprecipitation, library preparation, and sequencing

EndoC‐βH1 cells were washed twice using PBS Ca^2+^/Mg^2+^ free, and HBSS (14025092, Gibco) supplemented with HEPES (H0887, Euroclone) was added for 6 h to perform the starvation treatment. Then, 15 × 10^6^ cells were fixed with 1% formaldehyde for 15 min at room temperature and subsequently quenched using glycine 0.1 M. Cell lysis, nuclear extraction, and sample preparation were performed as previously described (Cesana *et al*, 2018). Chromatin sonication was performed using the Branson Ultrasonics™ Sonifier™ SFX150. Immunoprecipitation was performed using 20 μl per sample of TFEB antibody on two independent replicates. Libraries were prepared from 10 ng of DNA using the NEBNext UltraTM II DNA Library Prep Kit for Illumina (New England Biolabs) of each replicate and the corresponding input whole cellular extract. The quality of libraries was assessed using Bioanalyzer DNA Analysis (Agilent Technologies) and quantified using Qubit 4 Fluorometer (Thermo Fisher Scientific). Libraries were sequenced on a NovaSeq 6000 sequencing system using a paired‐end (PE) 100 cycles flow cell (Illumina Inc.). Paired sequencing reads were aligned on human hg38 reference genome using BWA (Li & Durbin, 2009) and filtered with samtools (Danecek *et al*, 2021) to remove unmapped read pairs, not primary alignments, reads failing platform quality, with mapping quality score below 30, and duplicate reads were then removed using picard MarkDuplicates [http://broadinstitute.github.io/picard].

Each sample (IP or input) was equally split by randomly assigning half of the read pairs to each of two pseudo‐replicates, the two biological replicates were pooled, and pools were split into two pseudo‐replicates as well. Irreproducible discovery rate analysis [IDR] (Li *et al*, 2011) was applied as described in the ENCODE guidelines (Landt *et al*, 2012). Peaks were called for each IP/input pair using MACS2 (Zhang *et al*, 2008) with a threshold *P* < 0.1, then IDR was performed using the *P*‐value as sorting column and an IDR score threshold of 0.01 on each of the following trio: (1) biological replicate 1 versus its own pseudo‐replicates; (2) biological replicate 2 versus its own pseudo‐replicates; (3) pool versus its own pseudo‐replicates; and (4) pool versus biological replicate 1 and biological replicate 2. As final peak selection, we merged the coordinates of peaks called (1), (2), and (4) and only kept those supported by at least two of the three analyses.

### Mouse maintenance

All procedures on mice were approved by the Italian Ministry of Health. Mice were housed at the TIGEM animal house under SPF certification. To generate mice, we bred the INS1Cre mice from the Thorens laboratory (B6(Cg)‐Ins1tm1.1(cre)Thor/J), with TFEB‐overexpressing mice (Settembre *et al*, 2011). For the generation of double‐KO mice, INS1Cre mice were crossed with TFEB conditional KO mice (Settembre *et al*, 2012) and TFE3 KO mice (Steingrimsson *et al*, 2002).

### Mouse metabolic phenotyping

All metabolic tests were performed in age‐matched, sex‐matched cohorts. All mice were bred on a pure C57Bl6/J genetic background. Glucose tolerance test (GTT) was performed by intraperitoneally injecting 2 g of glucose/kg body mass of an aqueous 20% glucose solution to overnight fasted mice. Prior to injection, fasting glycemia was measured using the Wellion® LUNA glucometer with the corresponding Wellion® LUNA test stripes GLU by collecting a blood drop from the tail. Glycemia was measured the same way at 15, 30, 60, 90, and 120 min after glucose injection. When applying, blood sampling from the tail for glucose‐stimulated insulin secretion (GSIS) was performed using Sarstedt Microvette CB300. Insulin tolerance test was performed in a similar way as the GTT, injecting 0.75 IU/kg body mass in 6 h fasted mice.

### Statistical analysis

Data are expressed as mean ± standard error. Statistical significance was calculated using the Student's two‐tailed *t*‐test or two‐way ANOVA as indicated in figure legends. A *P*‐value < 0.05 was considered statistically significant.

## Author contributions

**Adrien Pasquier:** Conceptualization; formal analysis; investigation; visualization; methodology; writing – original draft. **Nunzia Pastore:** Conceptualization; formal analysis; funding acquisition; validation; investigation; visualization; methodology; writing – original draft; writing – review and editing. **Luca D'Orsi:** Investigation; methodology. **Rita Colonna:** Validation; investigation. **Alessandra Esposito:** Validation; investigation; visualization. **Veronica Maffia:** Validation; investigation. **Rossella De Cegli:** Data curation; formal analysis; visualization. **Margherita Mutarelli:** Data curation; formal analysis. **Susanna Ambrosio:** Investigation. **Gennaro Tufano:** Investigation. **Antonio Grimaldi:** Data curation; formal analysis. **Marcella Cesana:** Investigation; methodology. **Davide Cacchiarelli:** Data curation; formal analysis. **Nathalie Delalleau:** Resources. **Gennaro Napolitano:** Conceptualization; resources; supervision; funding acquisition; writing – original draft; writing – review and editing. **Andrea Ballabio:** Conceptualization; resources; supervision; funding acquisition; writing – original draft; writing – review and editing.

## Disclosure and competing interests statement

A.B. is cofounder of CASMA Therapeutics, Inc., and Advisory board member of NexGeneration Diagnostics and Avilar Therapeutics.

## Supporting information



Expanded View Figures PDF

Table EV1

Dataset EV1

Dataset EV2

Dataset EV3

Dataset EV4

Dataset EV5

Dataset EV6

Dataset EV7

Dataset EV8

Dataset EV9

Dataset EV10

Dataset EV11

PDF+

Source Data for Figure 1

Source Data for Figure 2

Source Data for Figure 4

Source Data for Figure 5

## Data Availability

All the RNA‐seq datasets discussed in this work were deposited in GEO repository (Barrett *et al*, 2011). The titles of the dataset are as follows: “Transcriptome profile of INS‐1E Ctrl and hTFEB OE cells”, (GSE155221); “Transcriptome profile of ENDOC‐BH1 cells silencing TFEB and TFE3 (siTFEB/TFE3) or controls”, (GSE155220); “Transcriptome profile of INS‐1E Ctrl and DKO cells”, (GSE153709); “Transcriptome profile of ENDOC‐BH1 cells overexpressing TFEB‐V5 or controls”, (GSE154059); and “Transcriptome profile of isolated murine islets overexpressing TFEB or controls” (GSE154062). The SuperSeries GSE154063 whose title is “Transcriptome profile of ENDOC‐BH1 cells and isolated murine islets modulating TFEB expression” includes the three datasets GSE153709, GSE154059, and GSE154062.
