# Supplementary material for: TFEB and TFE3 control glucose homeostasis by regulating insulin gene expression
Source: EMBO J. 2023 Sep 15;42(21):e113928. doi: 10.15252/embj.2023113928 (PMC10620765; doi:10.15252/embj.2023113928)
Supplement: Supplementary file 1 — Expanded View Figures PDF [file EMBJ-42-e113928-s011.pdf]

Expanded View Figures

Figure EV1. TFEB controls INS mRNA levels in INS-1E cells.

- A Representative immunoblot of lysates from INS-1E cells infected with hTFEB-Flag-expressing lentivirus (TFEB-OE) or control (CTRL) cells.
- B Principal component analysis plot of transcriptomic data from TFEB-OE INS1 cells.
- C Gene ontology analysis for significantly upregulated (red) and downregulated (green) genes in TFEB-overexpressing (TFEB-OE) cells compared to control (CTRL) upon amino acid starvation (−aa) for 16 h.
- D INS1 and INS2 mRNA levels from control (CTRL) or TFEB-overexpressing (TFEB-OE) INS1 cells incubated with full medium (FED) or upon amino acid starvation (−aa) for 16 h. Each dot represents one mouse (n = 3-4/group). Data are represented as mean ± standard error. Student’s two-tailed t-test: ***P-value < 0.001.
- E Representative immunoblot for TFEB and TFE3 in DKO cells. GAPDH was used as a loading control.
- F Principal component analysis plot of transcriptomic data from TFEB/TFE3 DKO INS1 cells.
- G Gene enrichment analysis for significantly upregulated (green) and downregulated (red) genes from RNA seq data of DKO cells in comparison to control cells upon amino acid starvation (−aa) for 16 h.
- H INS1 and INS2 mRNA levels from CTRL or TFEB/TFE3-DKO INS1 cells incubated with full medium (FED) or upon amino acid starvation (−aa) for 16 h. Each dot represents one mouse (n = 3-4/group). Data are represented as mean ± standard error Student’s two-tailed t-test: *P-value < 0.05; ***P-value < 0.001.
- I Venn diagram showing the comparison of the datasets of TFEB-overexpressing (TFEB-OE) versus TFEB/3 DKO INS1 cells.
- J Heatmap showing the 550 DEGs significantly regulated in opposite correlation in TFEB-OE versus TFEB/3 DKO INS1 cells. The lanes corresponding to insulin genes (Ins1 and Ins2) are indicated.

© 2023 The Authors

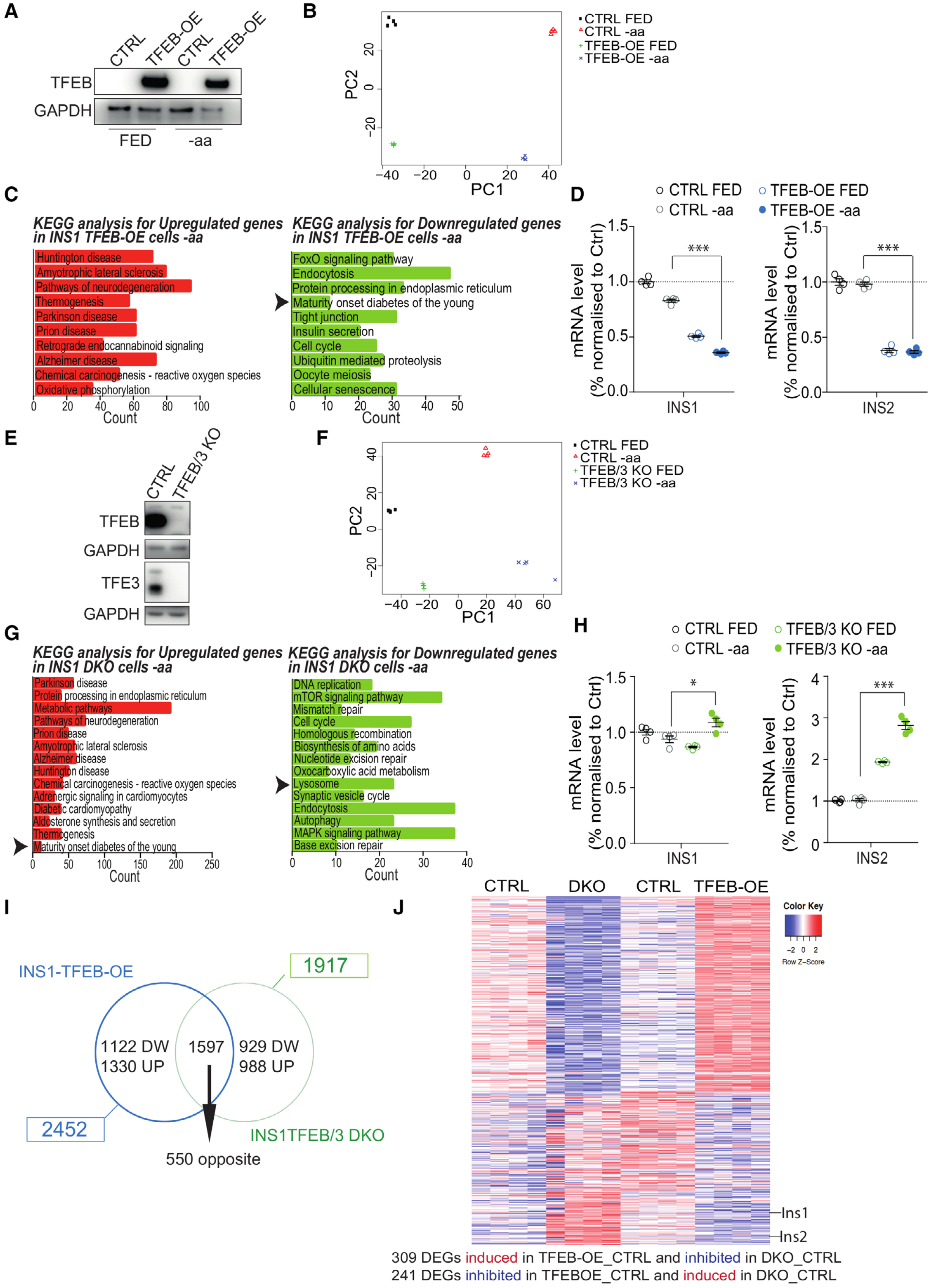
A
CTRL
TFEB-OE
CTRL
TFEB-OE
TFEB
GAPDH
FED
-aa
B
CTRL FED
CTRL -aa
TFEB-OE FED
TFEB-OE -aa
PC1
PC2
C
KEGG analysis for Upregulated genes in INS1 TFEB-OE cells -aa
Huntington disease
Amyotrophic lateral sclerosis
Pathways of neurodegeneration
Thermogenesis
Parkinson disease
Prion disease
Retrograde endocannabinoid signaling
Alzheimer disease
Chemical carcinogenesis - reactive oxygen species
Oxidative phosphorylation
Count
KEGG analysis for Downregulated genes in INS1 TFEB-OE cells -aa
FoxO signaling pathway
Endocytosis
Protein processing in endoplasmic reticulum
Maturity onset diabetes of the young
Tight junction
Insulin secretion
Cell cycle
Ubiquitin mediated proteolysis
Oocyte meiosis
Cellular senescence
Count
D
CTRL FED
CTRL -aa
TFEB-OE FED
TFEB-OE -aa
mRNA level (% normalised to Ctrl)
INS1
INS2
E
CTRL
TFEB/3 KO
TFEB
GAPDH
TFE3
GAPDH
F
CTRL FED
CTRL -aa
TFEB/3 KO FED
TFEB/3 KO -aa
PC1
PC2
G
KEGG analysis for Upregulated genes in INS1 DKO cells -aa
Parkinson disease
Protein processing in endoplasmic reticulum
Metabolic pathways
Pathways of neurodegeneration
Prion disease
Amyotrophic lateral sclerosis
Alzheimer disease
Huntington disease
Chemical carcinogenesis - reactive oxygen species
Adrenergic signaling in cardiomyocytes
Diabetic cardiomyopathy
Aldosterone synthesis and secretion
Thermogenesis
Maturity onset diabetes of the young
Count
KEGG analysis for Downregulated genes in INS1 DKO cells -aa
DNA replication
mTOR signaling pathway
Mismatch repair
Cell cycle
Homologous recombination
Biosynthesis of amino acids
Nucleotide excision repair
Oxocarboxylic acid metabolism
Lysosome
Synaptic vesicle cycle
Endocytosis
Autophagy
MAPK signaling pathway
Base excision repair
Count
H
CTRL FED
CTRL -aa
TFEB/3 KO FED
TFEB/3 KO -aa
mRNA level (% normalised to Ctrl)
INS1
INS2
I
INS1-TFEB-OE
1917
1122 DW
1330 UP
1597
929 DW
988 UP
2452
INS1TFEB/3 DKO
550 opposite
J
CTRL
DKO
CTRL
TFEB-OE
Color Key
Row Z-Score
Ins1
Ins2
309 DEGs induced in TFEB-OE_CTRL and inhibited in DKO_CTRL
241 DEGs inhibited in TFEBOE_CTRL and induced in DKO_CTRL

Figure EV1.

A

Lysosome	CD63, ASAH1, HEXB, HEXA, GBA, TCIRG1, NAGPA, GNS, SGSH, GGA2, LAPTM4B, CLN3, LAMP1, NAGLU, GM2A, CTSL, NEU1, PSAP, SLC17A5, ATP6V1H, ACP5, CTSF, CTSD, CTSB, CTSA, CD164, ATP6V0B, FUCA1, FUCA2, M6PR, CTNS, MCOLN1, IGF2R, SUMF1, GALNS, GNPTG, NPC2, PPT1, TPP1, ATP6V0D1, CD68, GLA
Autophagy	ATG3, GABARAPL2, UVRAG, ATG9B, RRAS2, IRS2, NRAS, TBK1, MRAS, LAMP1, RRAGC, RHEB, CTSL, BCL2, MAPK1, SMRC8, SQSTM1, CTSD, HRAS, SNAP29, CTSB, RAB7A
Oxidative Phosphorylation	ATP6V1G1, ATP6V0B, NDUFB8, ATP6V0E1, NDUFA1, COX7A2, TCIRG1, COX7C, COX5A, PPA1, UQCRQ, NDUFS6, ATP6V1H, CYC1, ATP6V1E1, ATP6V0D1, ATP6V1D, ATP6V1C1, ATP6V1F
Amino sugar and nucleotide sugar metabolism	CHIT1, GALE, GNPDA1, NAGK, HEXB, AMDHD2, HEXA, UAP1L1, PMM2, NPL, NANS
mTOR signalling pathway	ATP6V1G1, SLC3A2, NRAS, RRAGC, RHEB, RRAGD, DVL2, ATP6V1H, MAPK1, LAMTOR1, ATP6V1E1, LAMTOR4, ATP6V1D, HRAS, FNIP2, LAMTOR3, ATP6V1C1, ATP6V1F, LAMTOR5
Circadian rhythm	PER3, BHLHE40, BHLHE41, CRY1, PRKAG2, RORA, CSNK1D, NR1D1

Palmieri et al. 2011, Sardiello et al. 2009, Carey et al. 2020

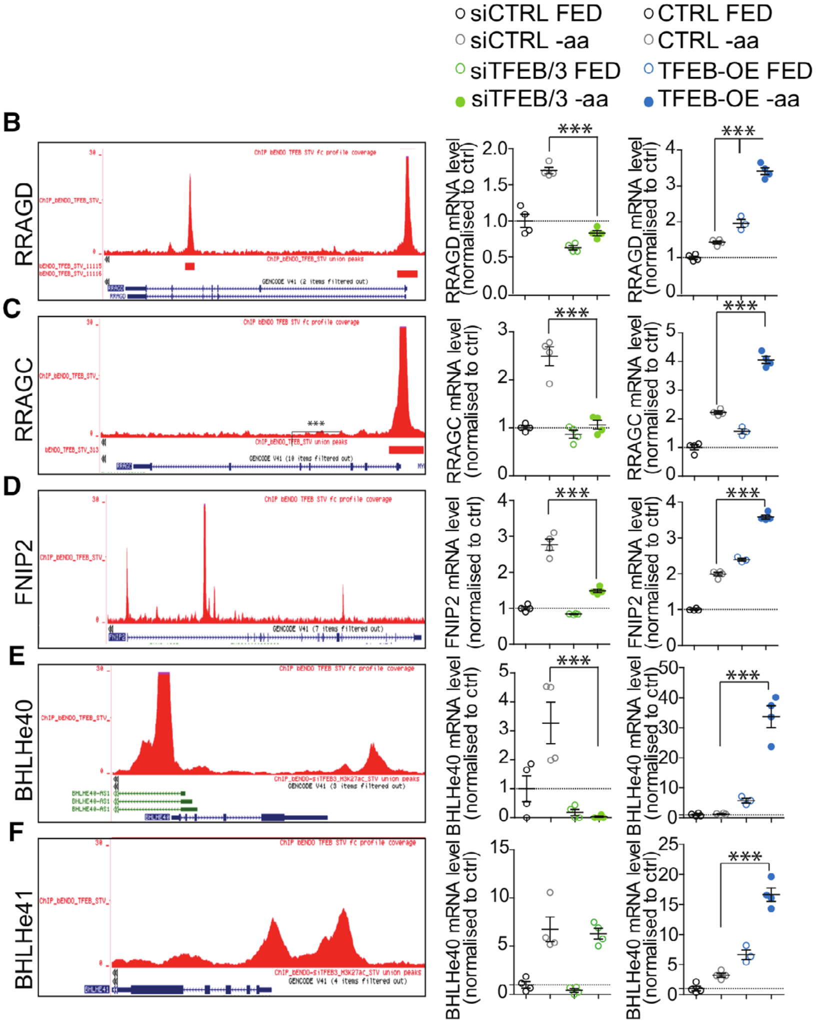

Figure EV2. ChIP-Seq analysis identifies direct TFEB targets in pancreatic beta cells.

A Top genes whose promoters are bound by TFEB upon fasting and upregulated upon TFEB upregulation in EndoC-BH1 cells sorted by gene categories. Genes in red are previously identified TFEB direct targets.

B–F On the left, alignment of the TFEB ChIP-seq track at the indicated gene loci. On the right, mRNA levels of the indicated genes from EndoC-βH1 cells treated with scramble siRNA (siCTRL) or TFEB- and TFE3-targeting siRNA (siTFEB/3) or TFEB-OE compared to control EndoC-βH1 cells incubated with full medium (FED) or upon amino acid starva tion (−aa) for 16 h. Data are represented as mean ± standard error. Each dot represents an independent experiment, ***$P < 0.001$; Student's two-tailed unpaired *t*-test.

 © 2023 The Authors

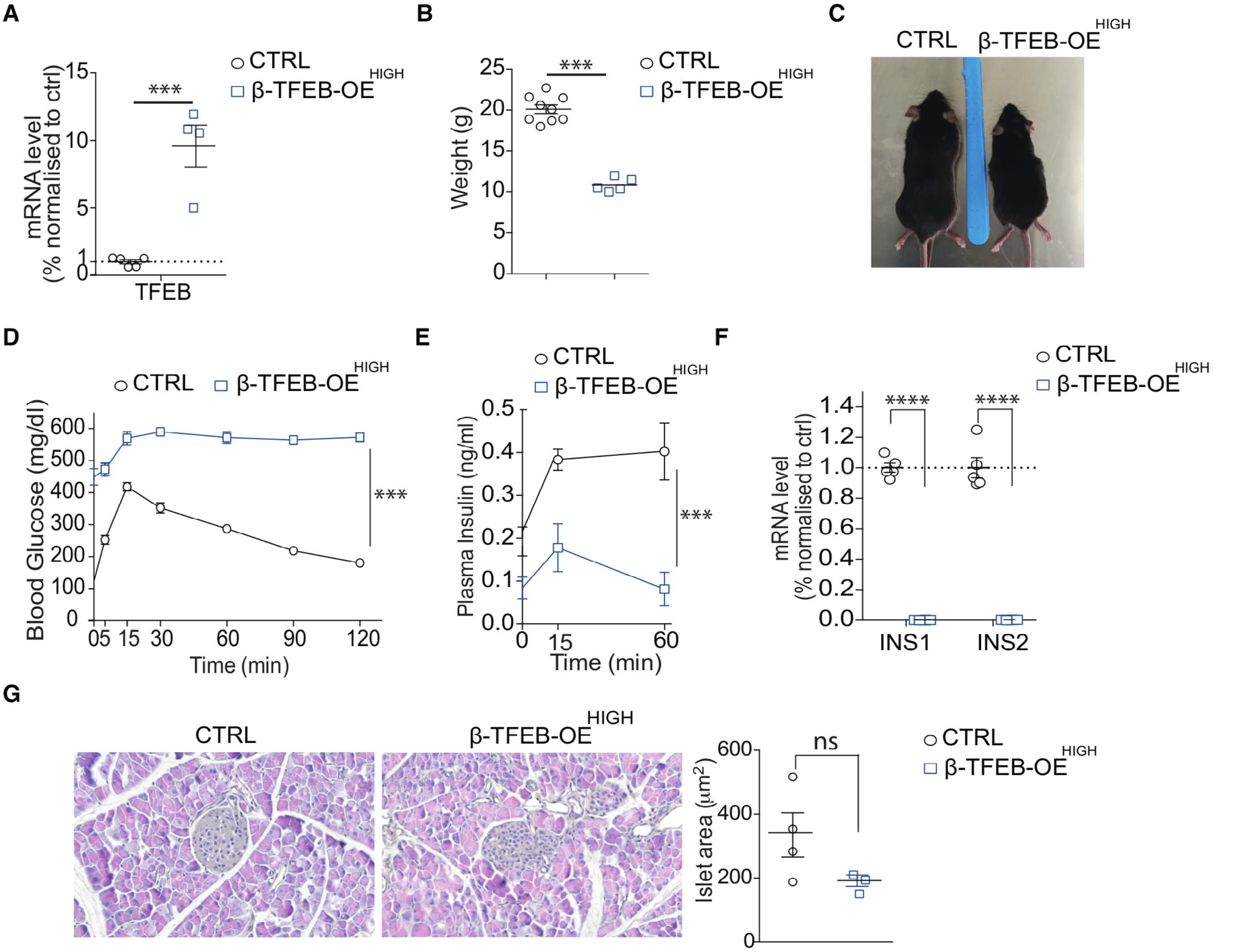

Figure EV3. TFEB upregulation in pancreatic β-cells in vivo results in suppression of insulin transcription and glucose intolerance.

A mRNA levels of TFEB in isolated islets from control and $\beta TFEBOE^{HIGH}$ mice. Each dot represents a mouse (n = 4-5/group). Data are represented as mean ± standard error. Student's two-tailed t-test: ***P-value < 0.001.

B Weight of control and $\beta TFEBOE^{HIGH}$ mice. Each dot represents a mouse (n = 9 for CTRL and n = 5 for $\beta TFEBOE^{HIGH}$ mice). Data are represented as mean ± standard error. Student's two-tailed t-test: ***P-value < 0.001.

C Representative images of control and $\beta TFEBOE^{HIGH}$ mice showing different body sizes.

D Glucose tolerance test (GTT) of control (n = 12) and $\beta TFEBOE^{HIGH}$ (n = 7) mice. Data are represented as mean ± standard error. Two-way ANOVA: ***P-value < 0.001.

E Glucose-stimulated insulin secretion (GSIS) for control (n = 11) and $\beta TFEBOE^{HIGH}$ (n = 7) mice. Data are represented as mean ± standard error. Two-way ANOVA: ***P-value < 0.001.

F mRNA levels of Ins1 and Ins2 in isolated islets from control and $\beta TFEBOE^{HIGH}$ mice. Each dot represents a mouse (n = 5/group). Data are represented as mean ± standard error. Student's two-tailed t-test: ****P-value < 0.0001.

G Representative images of pancreas slides from control and $\beta TFEBOE^{HIGH}$ mice, stained with hematoxylin/eosin and relative quantification of islet area (n = 3-4/group). Data are represented as mean ± standard error.

© 2023 The Authors
