# Supplementary material for: TFEB and TFE3 control glucose homeostasis by regulating insulin gene expression
Source: EMBO J. 2023 Sep 15;42(21):e113928. doi: 10.15252/embj.2023113928 (PMC10620765; doi:10.15252/embj.2023113928)
Supplement: Supplementary file 2 — Table EV1 [file EMBJ-42-e113928-s014.docx]

**Table EV1.** *Primers for real time PCR*

| **Gene** | **Species** |  | **Sequence (5’🡪 3’)** |
| --- | --- | --- | --- |
| *TFEB* | mouse | forward | GCAGAAGAAAGACAATCACAACC |
|  |  | reverse | GCCTTGGGGATCAGCATT |
| *HPRT* | mouse | forward | CAGTCCCAGCGTCGTGATTA |
|  |  | reverse | TCGAGCAAGTCTTTCAGTCCT |
| *S16* | mouse | forward | AGGAGCGATTTGCTGGTGTGG |
|  |  | reverse | GCTACCAGGGCCTTTGAGATG |
| *Ins1* | mouse | forward | AAGCTGGTGGGCATCCAGTAACC |
|  |  | reverse | GTTTGGGCTCCCAGAGGGCAAG |
| *Ins2* | mouse | forward | CCCTGCTGGCCCTGCTCTT |
|  |  | reverse | AGGTCTGAAGGTCACCTGCT |
| *TFE3* | mouse | forward | ACGATAGGATCAAAGAGCTGGGCA |
|  |  | reverse | ATCCACAGATGCCTTCAGGATGGT |
