# Supplementary material for: TFEB and TFE3 control glucose homeostasis by regulating insulin gene expression
Source: EMBO J. 2023 Sep 15;42(21):e113928. doi: 10.15252/embj.2023113928 (PMC10620765; doi:10.15252/embj.2023113928)
Supplement: Supplementary file 14 — PDF+ [file EMBJ-42-e113928-s002.pdf]

Article

SOURCE DATA TRANSPARENT PROCESS OPEN ACCESS

THE EMBO JOURNAL

TFEB and TFE3 control glucose homeostasis by regulating insulin gene expression

Adrien Pasquier[1,†], Nunzia Pastore[1,2,†], Luca D'Orsi[1], Rita Colonna[1], Alessandra Esposito[1], Veronica Maffia[1], Rossella De Cegli[1], Margherita Mutarelli[3], Susanna Ambrosio[1], Gennaro Tufano[1], Antonio Grimaldi[1], Marcella Cesana[1], Davide Cacchiarelli[1,2,4], Nathalie Delalleau[5,6], Gennaro Napolitano[1,2,4,*] & Andrea Ballabio[1,2,4,7,8,**]

Abstract

To fulfill their function, pancreatic beta cells require precise nutrient-sensing mechanisms that control insulin production. Transcription factor EB (TFEB) and its homolog TFE3 have emerged as crucial regulators of the adaptive response of cell metabolism to environmental cues. Here, we show that TFEB and TFE3 regulate beta-cell function and insulin gene expression in response to variations in nutrient availability. We found that nutrient deprivation in beta cells promoted TFEB/TFE3 activation, which resulted in suppression of insulin gene expression. TFEB overexpression was sufficient to inhibit insulin transcription, whereas beta cells depleted of both TFEB and TFE3 failed to suppress insulin gene expression in response to amino acid deprivation. Interestingly, ChIP-seq analysis showed binding of TFEB to super-enhancer regions that regulate insulin transcription. Conditional, beta-cell-specific, *Tfeb*-overexpressing, and *Tfeb/Tfe3* double-KO mice showed severe alteration of insulin transcription, secretion, and glucose tolerance, indicating that TFEB and TFE3 are important physiological mediators of pancreatic function. Our findings reveal a nutrient-controlled transcriptional mechanism that regulates insulin production, thus playing a key role in glucose homeostasis at both cellular and organismal levels.

Keywords beta cells; glucose homeostasis; insulin; mTORC1; TFEB
Subject Categories Chromatin, Transcription & Genomics; Metabolism
DOI 10.15252/embj.2023113928 | Received 2 March 2023 | Revised 31 July 2023 | Accepted 25 August 2023
The EMBO Journal (2023) e113928

Introduction

Pancreatic β-cells play a crucial role in organismal adaptation to nutrient availability by secreting the central hormone insulin. Consistently, β-cell failure results in dysregulation of glucose homeostasis and is the hallmark of diabetes (Cnop *et al*, 2005; Vasiljevic *et al*, 2020; Gloyn *et al*, 2022). Thus, understanding how β-cells orchestrate the metabolic response to nutrient availability is of pivotal importance. The nutrient-sensitive transcription factor EB (TFEB), as well as TFE3, another member of the MiT/TFE family, are major controllers of cell metabolism (Napolitano & Ballabio, 2016; Puertollano *et al*, 2018). TFEB and TFE3 regulate cell metabolism at multiple levels, such as lysosomal biogenesis (Sardiello *et al*, 2009), autophagy (Settembre *et al*, 2011), lipid catabolism (Settembre *et al*, 2013; Pastore *et al*, 2017), circadian rhythm (Pastore *et al*, 2019), mitochondrial biogenesis (Mansueto *et al*, 2017), as well as whole energy metabolism (Pastore *et al*, 2017). TFEB and TFE3 subcellular localization and activities are regulated by the mechanistic target of rapamycin complex 1 (mTORC1; Martina *et al*, 2012, 2014; Roczniak-Ferguson *et al*, 2012; Settembre *et al*, 2012), which is recruited and activated at the lysosomal surface in response to increased levels of amino acids (Sancak *et al*, 2008, 2010), glucose (Orozco *et al*, 2020; Yoon *et al*, 2020), and other nutrients (Castellano *et al*, 2017; Liu & Sabatini, 2020). Gain- and loss-of-function experiments in mice have shown that mTORC1 activity is crucial for maintaining pancreatic β-cell homeostasis by modulating insulin mRNA translation and β-cell mass (Rachdi *et al*, 2008; Mori *et al*, 2009; Blandino-Rosano *et al*, 2017). mTORC1 inhibition during amino acid and glucose starvation leads to TFEB/TFE3 dephosphorylation, cytoplasm-to-nucleus translocation, and activation of their transcriptional program. Previous studies showed

1 Telethon Institute of Genetics and Medicine (TIGEM), Naples, Italy
2 Medical Genetics Unit, Department of Medical and Translational Science, Federico II University, Naples, Italy
3 Institute of Applied Sciences and Intelligent Systems, National Research Council (ISASI-CNR), Pozzuoli, Italy
4 School for Advanced Studies, Genomics and Experimental Medicine Program, University of Naples "Federico II", Naples, Italy
5 University of Lille, U1190-EGID, Lille, France
6 Inserm, U1190, Lille, France
7 Department of Molecular and Human Genetics, Baylor College of Medicine, Houston, TX, USA
8 Jan and Dan Duncan Neurological Research Institute, Texas Children's Hospital, Houston, TX, USA
*Corresponding author. Tel: +39 081 19230621; E-mail: g.napolitano@tigem.it
**Corresponding author. Tel: +39 081 19230607; E-mail: ballabio@tigem.it
[†]These authors contributed equally to this work

© 2023 The Authors. Published under the terms of the CC BY 4.0 license

that TFEB/TFE3 transcriptional programs vary in different cell types, suggesting specialized functions of these transcription factors in different tissues (Napolitano & Ballabio, 2016). However, whether TFEB and TFE3 play specific roles in the maintenance of pancreatic endocrine function, which is crucial for organismal metabolism and adaptation to nutrient availability, has not been recognized thus far.

Here, we show that TFEB and TFE3 modulate insulin gene expression in response to changes in nutrient availability. We found that TFEB overexpression was sufficient to suppress insulin transcription, whereas β-cells lacking both TFEB and TFE3 failed to suppress insulin gene expression in response to nutrient starvation, both *in vitro* and *in vivo*, resulting in impaired glucose homeostasis. Thus, in addition to their well-established role in promoting cell catabolic pathways, our data reveal that TFEB and TFE3 also function as suppressors of cell anabolism via inhibition of insulin production.

Results

TFEB and TFE3 activity is controlled by nutrient availability and mTORC1 activity in pancreatic β-cells

To investigate the role of TFEB and TFE3 in pancreatic β-cells, we analyzed the regulation and downstream transcriptional programs of these transcription factors in the human EndoC-βH1 and rat INS-1E β-cell lines (Ravassard *et al*, 2011; Tsonkova *et al*, 2018). Similar to other cell types (Settembre *et al*, 2011, 2012; Martina *et al*, 2012; Li *et al*, 2018; Napolitano *et al*, 2018), TFEB localized to the cytosol of fully fed or refed EndoC-βH1 cells, whereas it translocated to the nucleus upon either amino acid or glucose starvation (Fig 1A). Consistently, phosphorylation of endogenous TFEB in EndoC-βH1 cells, which was measured by analyzing its molecular weight shift (Settembre *et al*, 2012; Napolitano *et al*, 2020), was dependent on both nutrient availability and mTORC1 activity (Fig 1B). Similar results were also obtained in subcellular fractionation experiments using the INS-1E β-cell line, which showed dephosphorylation and nuclear translocation of both TFEB and TFE3 upon nutrient deprivation (Fig 1C). Notably, TFEB nuclear localization in glucose-starved EndoC-βH1 cells was completely rescued upon expression of constitutively active forms of the Rag GTPases (Fig 1D), which are known modulators of mTORC1-mediated TFEB phosphorylation in other cell types (Martina & Puertollano, 2013; Napolitano *et al*, 2020, 2022; Li *et al*, 2022; Cui *et al*, 2023). These data indicate that TFEB and TFE3 are modulated in pancreatic β-cells via nutrient- and Rag GTPase-dependent activation of mTORC1.

TFEB and TFE3 control insulin gene expression in β-cells

Next, we analyzed the global transcriptional network modulated by TFEB in β-cells using both gain- and loss-of-function approaches. We performed RNA sequencing (RNA-seq) experiments upon overexpression of TFEB (TFEB^{OE}), using a lentiviral vector containing a doxycycline-dependent TFEB-V5-expressing cassette, in fed or starved EndoC-βH1 cells (Fig 2A and B). Principal component analysis (PCA) revealed that our samples were segregated into four main groups, suggesting that both amino acid starvation and TFEB overexpression profoundly remodeled the entire transcriptome (Fig 2C). Consistent with the role of TFEB as a master regulator of lysosomal function (Sardiello *et al*, 2009), gene set enrichment analysis (GSEA) revealed that the "Lysosome" was the most significantly upregulated gene category (Fig 2D and Dataset EV1). Notably, we also found that the "Maturity Onset Diabetes of the Young (MODY)" gene category, which contains genes important for pancreatic β-cells, including insulin, was among the downregulated enriched gene categories in TFEB-overexpressing cells (Fig 2D). In line with these results, TFEB overexpression was sufficient to promote a striking reduction in insulin mRNA levels in fully fed cells, which was comparable to the one observed in fasted control cells (Fig 2E).

In parallel to the gain-of-function experiments, we performed transcriptome analysis of EndoC-βH1 cells concomitantly silenced for both TFEB and TFE3 (siTFEB/3), to avoid reciprocal compensation (Brady *et al*, 2018; El-Houjeiri *et al*, 2019; Pastore *et al*, 2019), and cultured in the presence or absence of amino acids (Fig 2F and G). Also in this case, PCA showed strong segregation of the experimental groups, confirming that TFEB and TFE3 play a key role in modulating the response of gene expression to nutrients in β-cells (Fig 2H and I and Dataset EV2). Notably, whereas prolonged amino acid starvation reduced insulin mRNA levels in EndoC-βH1 cells (Fig 2J), as previously reported (Iwashima *et al*, 1994; Boland *et al*, 2018), such decrease was prevented by depletion of TFEB and TFE3, indicating that these transcription factors are important mediators of insulin suppression upon nutrient deprivation (Fig 2J).

Figure 1. TFEB responds to nutrient variation in pancreatic β-cells.

A Representative images of high content analyses using TFEB antibodies on fixed cells previously treated with full medium (FED), without amino acids (−aa) for 1 h, without glucose (−glu) for 1 h, without amino acids and glucose (−aa -glu) for 1 h or starved for 1 h and restimulated with amino acids for 30′ (−aa/Refeeding). The relative quantification (see Materials and Methods) is shown in the graph. Data are represented as mean ± standard error. Each dot represents a well from the 96-well plate (n = 30 fed, n = 12 −aa, n = 14 −glu, n = 15 −aa −glu, and n = 5 −aa/Refeeding). The experiment was repeated independently three different times. Scale bar: 20 µm. Student's two-tailed t-test: ***P-value < 0.001.

B Representative immunoblot using the indicated antibodies from lysates of EndoC-βH1 cells starved for 1 h of glucose or starved for 1 h and restimulated for 30′ with glucose in the presence or absence of Torin1.

C Representative immunoblot for TFEB and TFE3 using lysates from nuclear and cytosolic fractions of INS-1E cells treated with full medium (FED) or upon amino acid starvation (−aa). PARP1 was used as nuclear fraction loading control, and GAPDH was used as cytosol fraction loading control. Cytosolic fraction (C), Nuclear fraction (N).

D Representative immunofluorescence analysis using TFEB antibodies on fixed EndoC-βH1-cells treated with full medium (FED) or upon glucose starvation, prior transient transfection with constitutively active Rag GTPase mutants (i.e., HA-GST-RagA-Q66L; HA-GST-RagC-S75L). Transfected cells are indicated with a white arrow. Data are represented as mean ± standard error. Each dot represents a separate field of a representative experiment (n = 5). At least 52 cells were counted in each condition.

Source data are available online for this figure.

 © 2023 The Authors

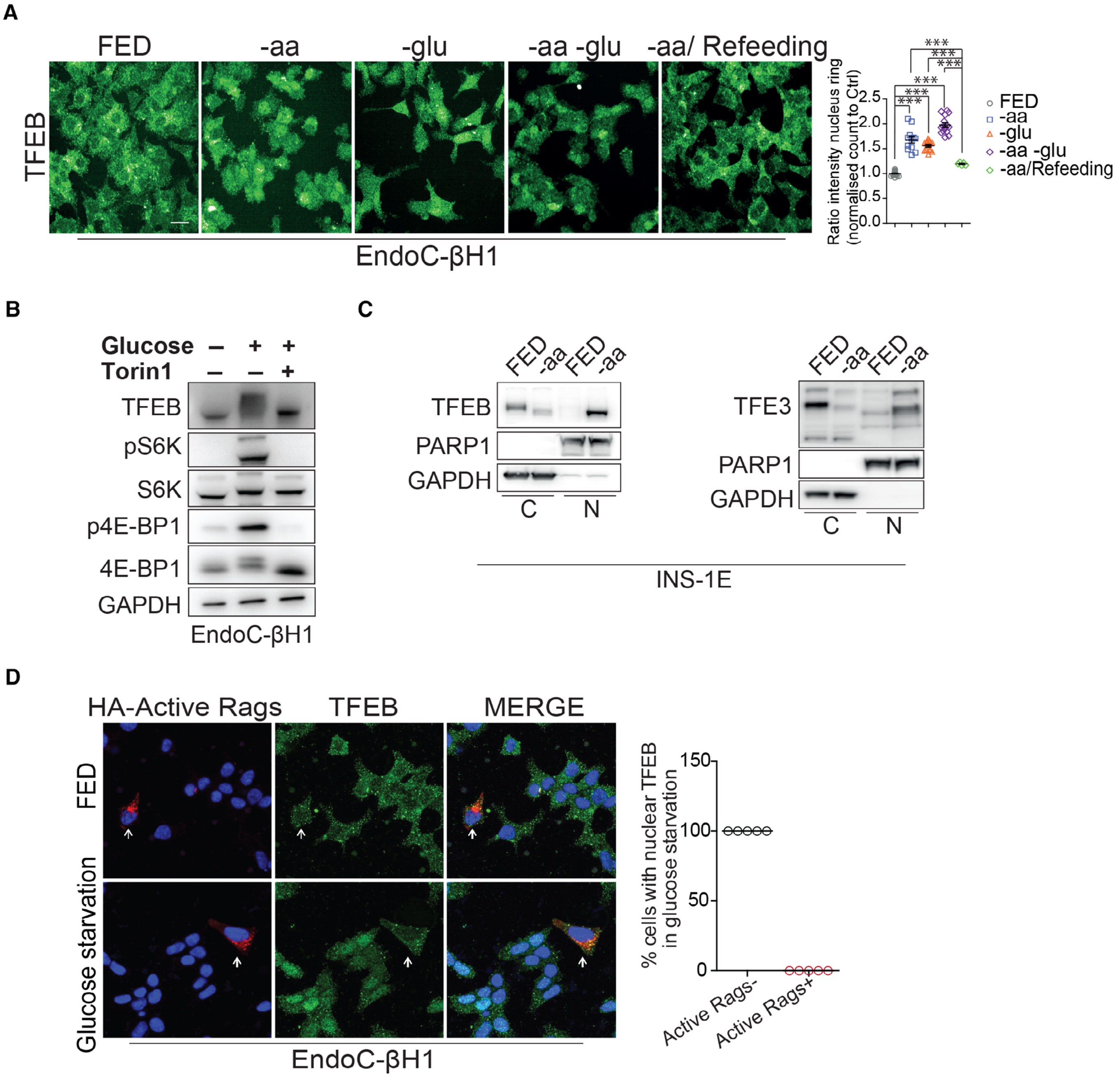

Figure 1.

Similar results were also obtained by performing transcriptome analysis upon TFEB overexpression in INS-1E cells (Fig EV1A–C and Dataset EV3), another relevant pancreatic β-cell line. Importantly, "MODY" was among the downregulated categories in TFEB^{OE} cells compared to control cells (Fig EV1C). Accordingly, TFEB^{OE} caused a strong downregulation of both INS1 and INS2 genes (Fig EV1D). We also performed transcriptome analysis of rat INS-1E cells depleted for both TFEB and TFE3 (TFEB/TFE3-DKO cells), generated via the CRISPR/Cas9 system (Fig EV1E–G and Dataset EV4) and found significant upregulation of both *INS1* and *INS2* genes (Fig EV1H), consistent with the results generated in EndoC-βH1 cells.

Comparison analysis of DEGs that are common to TFEB^{OE} and TFEB/TFE3 depletion upon nutrient deprivation in EndoC-βH1 (Fig 2K and L and Dataset EV5 and EV6) and INS-1E (Fig EV1I and J and Dataset EV7 and EV8) cells revealed the presence of 751 and 550 oppositely correlated genes, respectively. GSEA analysis of these genes revealed that "Lysosome" was among the most upregulated gene category in TFEB^{OE} and the most significantly downregulated in TFEB/TFE3-depleted cells, as expected (Fig 2M and Dataset EV9). Notably, insulin was among the top downregulated in TFEB^{OE} and top upregulated genes in TFEB/TFE3-depleted cells (Fig 2L).

© 2023 The Authors

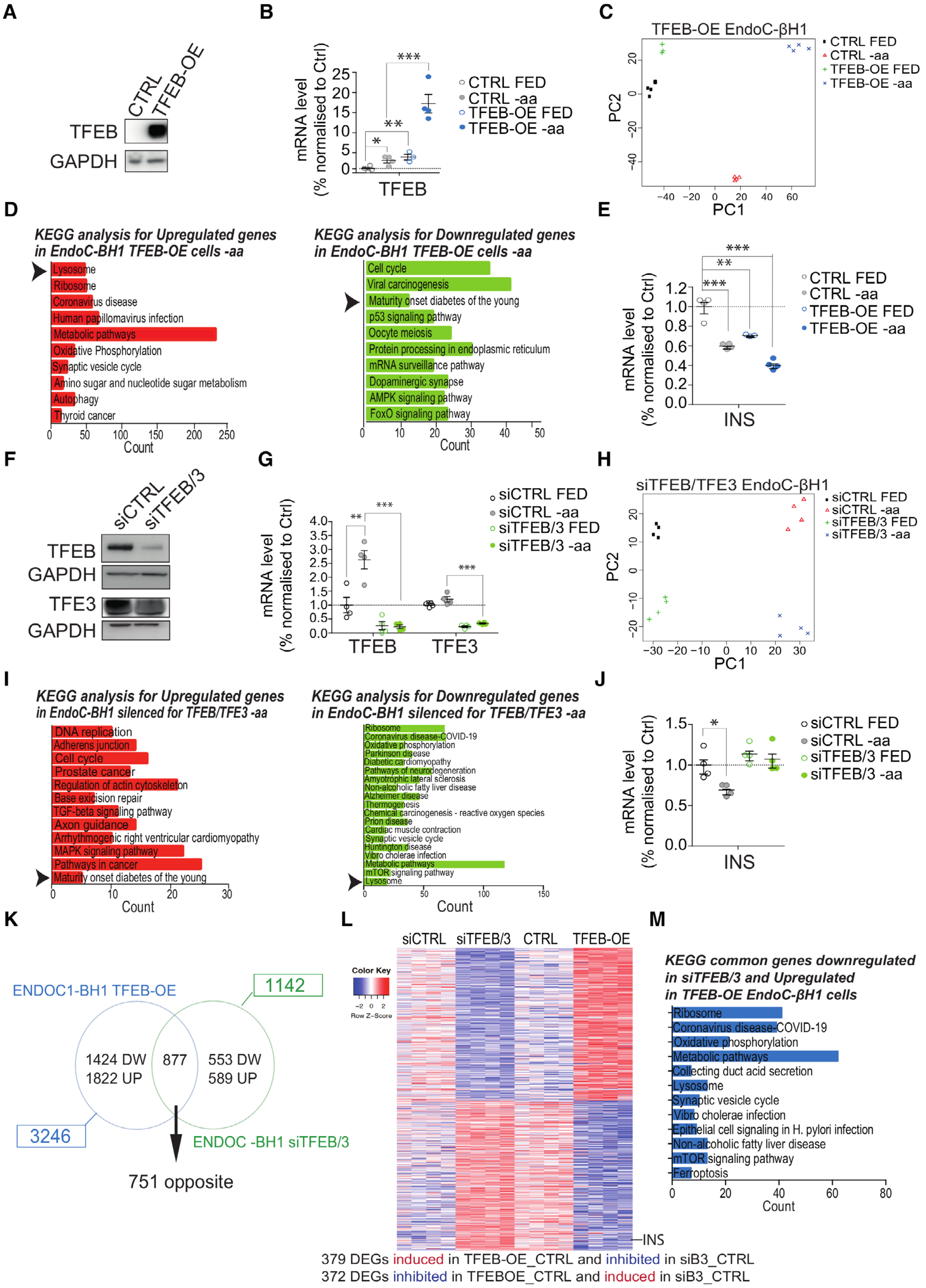

Figure 2.

 © 2023 The Authors

◀ **Figure 2. TFEB controls insulin mRNA levels in EndoC-BH1 cells.**

A Representative immunoblot using lysates from cells infected with control lentivirus (CTRL) of TFEB-V5-expressing lentivirus (TFEB-OE) and analyzed using a V5 antibody. GAPDH was used as a loading control.

B TFEB mRNA levels in TFEB-OE and CTRL EndoC-βH1 cells incubated with full medium (FED) or starved of amino acids (−aa) for 16 h. Data are represented as mean ± standard error (n = 3–4/group). Student's two-tailed t-test: *P-value < 0.05; **P-value < 0.01; ***P-value < 0.001.

C Principal component analysis plot of transcriptomic data from TFEB-OE EndoC-βH1 cells.

D Gene ontology analysis for significantly upregulated (red) and downregulated (green) genes in TFEB-OE cells compared to control upon amino acid starvation (−aa) for 16 h. Statistically significant hits (FDR < 0.05) are ranked from the most to the least significant.

E INS mRNA levels from control (CTRL) and TFEB-overexpressing (TFEB-OE) EndoC-βH1 cells incubated with full medium (FED) or starved of amino acids (−aa) for 16 h. Data are represented as mean ± standard error (n = 3–4/group). Student's two-tailed t-test: **P-value < 0.01; ***P-value < 0.001.

F Representative immunoblot for TFEB and TFE3 in EndoC-βH1 cells treated with scramble siRNA (siCTRL) or TFEB- and TFE3-targeting siRNA (siTFEB/3). GAPDH was used as a loading control.

G TFEB and TFE3 mRNA levels in siTFEB/3 and control EndoC-βH1 cells incubated with full medium (FED) or starved of amino acids (−aa) for 16 h. Data are represented as mean ± standard error (n = 4/group). Student's two-tailed t-test: **P-value < 0.01; ***P-value < 0.001.

H Principal component analysis plot of transcriptomic data from siTFEB/TFE3 EndoC-βH1 cells.

I Gene ontology analysis for significantly upregulated (red) and downregulated (green) genes in cells depleted for TFEB and TFE3 compared to control upon amino acid starvation (−aa) for 16 h.

J INS mRNA levels from EndoC-βH1 cells treated with scramble siRNA (siCTRL) or TFEB and TFE3-targeting siRNA (siTFEB/3) incubated with full medium (FED) or starved of amino acids (−aa) for 16 h. Data are represented as mean ± standard error (n = 4/group). Student's two-tailed t-test: *P-value < 0.05.

K Venn diagram showing the comparison of the datasets of TFEB-OE versus TFEB/3-depleted EndoC-βH1 cells.

L Heatmap showing the 751 DEGs significantly regulated in opposite correlation in TFEB-OE versus TFEB/3-depleted EndoC-βH1 cells. The lane corresponding to the insulin gene (INS) is indicated.

M Gene ontology analysis for significantly downregulated genes in siTFEB/3 and upregulated in TFEB-OE cells in opposite correlation.

Source data are available online for this figure.

Overall, these results suggest that TFEB and TFE3 play an important role in the transcriptional regulation of insulin.

TFEB binds to super-enhancer regions responsible for modulation of insulin transcription

TFEB has been known to act as a positive regulator of transcription by binding to proximal promoters (Sardiello & Ballabio, 2009; Palmieri *et al*, 2011). Thus, the evidence that TFEB in β-cells can work as a negative transcriptional regulator of insulin was surprising and prompted us to determine whether TFEB exerts this function directly or indirectly. To this end, we performed ChIP-seq analysis for endogenous TFEB in EndoC-βH1 upon amino acid starvation. We found that TFEB binds to the proximal promoter regions (i.e., < 300 bp from the transcriptional start site) of 4,765 genes in β-cells. Consistent with previous results obtained in other cell types and tissues (Sardiello *et al*, 2009; Palmieri *et al*, 2011; Brooks & Dang, 2019; Pastore *et al*, 2019; Gambardella *et al*, 2020), the comparison of genes upregulated upon TFEB gain of function (logFC>1) and downregulated upon TFEB/TFE3 depletion with genes containing at least one TFEB binding site in their proximal promoter (Fig 3A and B) revealed an enrichment of known TFEB-regulated gene categories, including "Lysosome," "Metabolic pathways," and "circadian rhythm" (Figs 3C and EV2 and Dataset EV10). However, comparison of genes that were downregulated in our RNA-seq data upon TFEB overexpression with our ChIP-seq data failed to reveal TFEB binding sites in insulin promoter (Fig 3D) despite the evidence that the insulin gene was under TFEB transcriptional control. These data suggested that TFEB effect on INS transcription was not caused by direct promoter binding. Of note, our ChIP-seq data revealed that only approximately 40% of TFEB binding sites were in promoter regions (< 2 kb from the TSS) in pancreatic β-cells (Fig 3A). Interestingly, we found that TFEB binds to conserved super-enhancer (SE) regions previously identified both in the EndoC-βH1 cell line and in β-cells obtained from human islets and hESC (Mularoni *et al*, 2017; Lawlor *et al*, 2019). By comparing our ChIP-seq data with available ChIP-seq and ChiA-Pet data collected on the *Islet Regulome browser* (http://pasqualilab.upf.edu/app/isletregulome) and the *Shiny App for Visualizing EndoC-βH1 and Human Islet Genomics Data* (https://shinyapps.jax.org/endoc-islet-multi-omics/) databases (Mularoni *et al*, 2017, Lawlor *et al*, 2019), we found that TFEB binds in at least four different sites of a super-enhancer region in the close vicinity of the *INS* locus in EndoC-βH1 cells (Fig 3D). Interestingly, two out of the four sites are specifically bound by TFEB, as no other well-known *INS* transcriptional modulator, such as MAFB, NKX2-2, NKX6-1, and PDX1, has been reported to bind these sites in other ChIP-Seq experiments thus far (Fig 3E). These data suggest a possible mechanism by which TFEB suppresses insulin gene transcription.

TFEB and TFE3 control insulin production and glucose homeostasis *in vivo*

To assess the physiological relevance of TFEB on INS transcription and β-cell function, we performed *in vivo* gain-of-function experiments by generating mouse models in which TFEB was specifically overexpressed in β-cells. This was achieved by breeding INS1-CRE mice (Thorens *et al*, 2015) with two different conditional TFEB overexpressing mouse lines ($TFEBOE^{LOW}$ and $TFEBOE^{HIGH}$), previously generated in our laboratory (Settembre *et al*, 2013), leading to the generation of the β-$TFEBOE^{LOW}$ and β-$TFEBOE^{HIGH}$ mouse lines in which TFEB was overexpressed specifically in β-cells at low and high levels, respectively. β-$TFEBOE^{LOW}$ mice showed a ~ 4-fold increase in TFEB mRNA levels in pancreatic β-cells compared to controls (Fig 4A), which was associated with smaller size and lower weight compared to control littermates (Fig 4B and C). Strikingly, these mice also showed profound glucose intolerance (GTT) (Fig 4D), impaired glucose-stimulated insulin secretion (GSIS) (Fig 4E), and insulin resistance (Fig 4F). Hematoxylin/Eosin (HE) staining on fixed pancreas revealed the presence of numerous

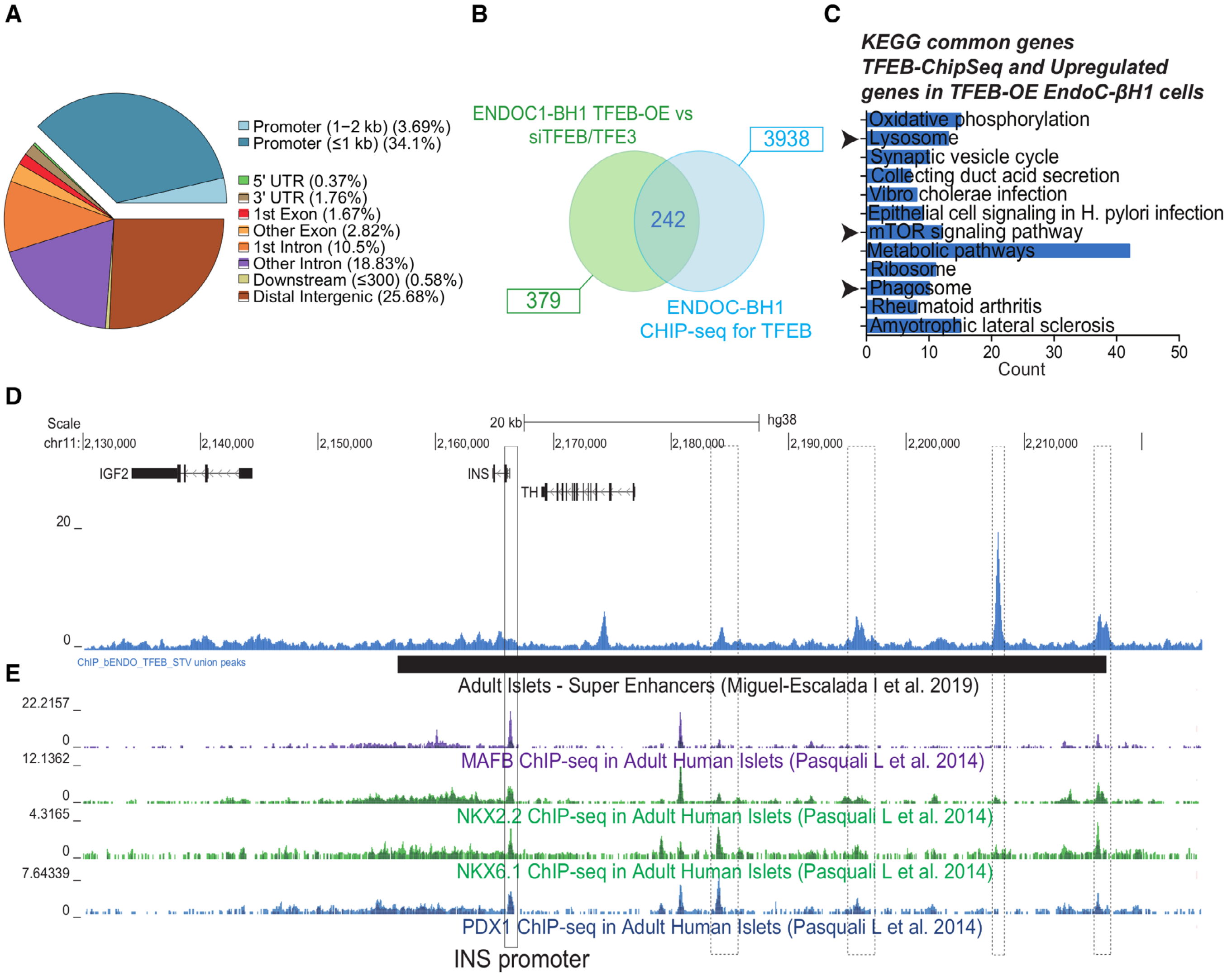

Figure 3. ChIP-seq analysis identifies TFEB-binding sites in insulin super-enhancer.

A Graphic representation of the different genomic sites of TFEB binding in EndoC-βH1 cells upon 16 h amino acid starvation.

B Venn diagram of genes upregulated upon TFEB overexpression and downregulated in siTFEB/3 and TFEB-binding sites located inside proximal promoters (−1,000 + 300).

C Gene ontology analysis for TFEB promoter-bound direct targets.

D, E Alignment of the TFEB ChIP-seq track, aligned with ChIP-seq tracks of other known INS regulators (MAFB, NKX2.2, NKX6.1, and PDX1), at the INS locus. Super-enhancer, black box.

regular-size islets in both β-TFEBOE^{LOW} and control mice (Fig 4G). However, immunofluorescence analysis using insulin and glucagon antibodies showed a marked reduction in insulin signal in β-cells of β-TFEBOE^{LOW} compared to control mice, with a slightly increased glucagon staining in α-cells in the islet periphery (Fig 4H). Importantly, despite the reduced levels of insulin expression in β-cells observed in β-TFEBOE^{LOW} islets, immunostaining analysis using an antibody recognizing UCN3, an established marker of mature β-cells (van der Meulen *et al*, 2012), revealed no effects of TFEB overexpression on β-cell differentiation (Fig 4I), thus suggesting that β-cells produce less insulin despite having a normal islet architecture. Consistent with increased number of α-cells, TFEB overexpression resulted in a slight but significant reduction in β-cell area quantified as UCN3^{+} staining, indicating that β-cell mass is reduced in β-TFEBOE^{LOW} mice (Fig 4J) likely as a secondary effect of the diabetic phenotype. Together, these results indicate that TFEB plays an important physiological role in β-cell function. Accordingly, and in line with the results obtained in cell lines, RNA-seq analysis of islets isolated from β-TFEBOE^{LOW} mice showed severe downregulation of the "MODY" gene category (Fig 4K and Dataset EV11). In addition, mRNA levels of *INS1* and *INS2* were strongly downregulated in β-TFEBOE^{LOW} mice compared to controls (Fig 4L).

Analysis of β-TFEBOE^{HIGH} mice showed higher levels of TFEB compared to β-TFEBOE^{LOW} mice (Fig EV3A), as expected. This resulted in a similar but more severe phenotype characterized by heavily impaired growth (Fig EV3B and C), greater glucose

 © 2023 The Authors

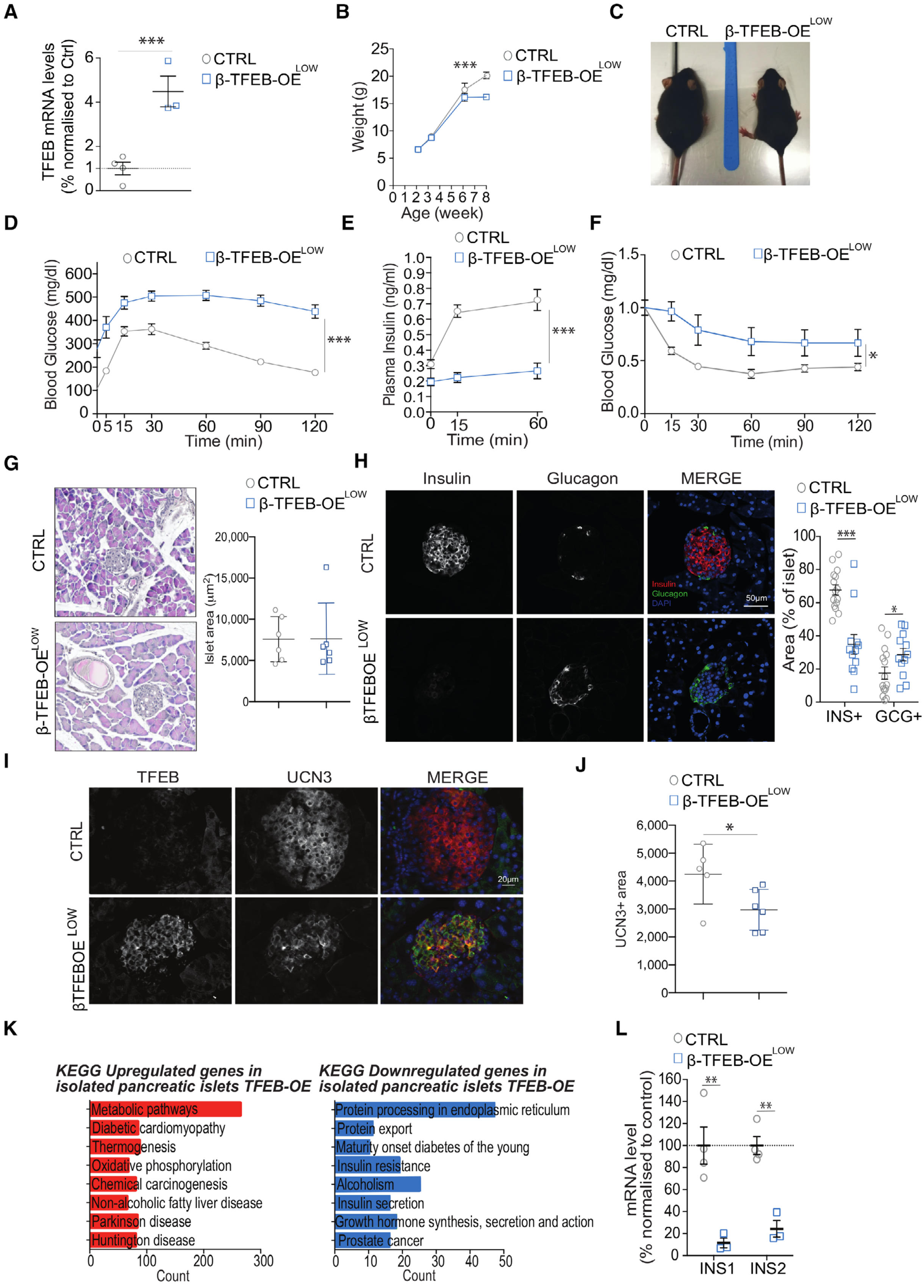

A
B
C
CTRL
β-TFEB-OE LOW
D
E
F
G
H
Insulin
Glucagon
MERGE
I
TFEB
UCN3
J
K
KEGG Upregulated genes in isolated pancreatic islets TFEB-OE
KEGG Downregulated genes in isolated pancreatic islets TFEB-OE
Metabolic pathways
Diabetic cardiomyopathy
Thermogenesis
Oxidative phosphorylation
Chemical carcinogenesis
Non-alcoholic fatty liver disease
Parkinson disease
Huntington disease
Protein processing in endoplasmic reticulum
Protein export
Maturity onset diabetes of the young
Insulin resistance
Alcoholism
Insulin secretion
Growth hormone synthesis, secretion and action
Prostate cancer
Count
L
INS1
INS2

Figure 4.

Figure 4. Beta-cell-specific TFEB gain- or TFEB/TFE3 loss-of-function *in vivo* leads to impairment of insulin production and glucose homeostasis.

A mRNA levels of TFEB in isolated islets from control and βTFEBOE^{LOW} mice. Each dot represents one mouse (n = 3–4/group). Data are represented as mean ± standard error. Student's two-tailed t-test: ***P-value < 0.001.

B Weight gain of control (n = 20) and βTFEBOE^{LOW} mice (n = 14). Data are represented as mean ± standard error. Two-way ANOVA: ***P-value < 0.001.

C Representative images of control and βTFEBOE^{LOW} mice showing different body sizes.

D Glucose tolerance test (GTT) of control (n = 15) and βTFEBOE^{LOW} (n = 11) mice. Data are represented as mean ± standard error. Two-way ANOVA: ***P-value < 0.001.

E Glucose-stimulated insulin secretion (GSIS) for control (n = 9) and βTFEBOE^{LOW} (n = 7) mice. Data are represented as mean ± standard error. Two-way ANOVA: ***P-value < 0.001.

F Insulin tolerance test (ITT) normalized to T0 in control and βTFEBOE^{LOW} mice (n = 9/group). Data are represented as mean ± standard error. Two-way ANOVA: *P-value < 0.05.

G Representative images of pancreas slides from control and βTFEBOE^{LOW} mice stained with hematoxylin/eosin and quantification of islet area (n = 6/group; n = 5–10 islets per mouse). Data are represented as mean ± standard error.

H Representative immunofluorescence picture using insulin and glucagon antibodies on fixed pancreas of control and βTFEBOE^{LOW} mice (scale bar 50 μm). The graph shows the quantification of the relative insulin (INS$^+$)- and glucagon (GCG$^+$)-positive area from islets of control and βTFEBOE^{LOW} mice. Each dot represents an islet (n = 15 for CTRL and n = 12 for βTFEBOE^{LOW}). Data are represented as mean ± standard error. Student's two-tailed t-test: ***P-value < 0.001.

I, J Representative immunofluorescence images for TFEB and UCN3 (I) on fixed pancreas of control and βTFEBOE^{LOW} mice (scale bar 20 μm) and quantification of the UCN3$^+$ area per islet in control and βTFEBOE^{LOW} pancreas (n = 3/group; n = 3–14 islets per mouse) (J). Data are represented as mean ± standard error. Student's two-tailed t-test: *P-value < 0.05.

K Gene ontology analysis of RNA-seq data of isolated islets from control and βTFEBOE^{LOW} mice.

L mRNA levels of indicated genes assessed on isolated islets from control and βTFEBOE^{LOW} mice. Each dot represents one mouse (n = 3–4/group). Data are represented as mean ± standard error. Student's two-tailed t-test: **P-value < 0.01.

Source data are available online for this figure.

intolerance, and impaired insulin secretion (Fig EV3D and E), as well as complete absence of *INS1*/*INS2* expression (Fig EV3F). As in β-TFEBOE^{LOW} mice, islet area of β-TFEBOE^{HIGH} mice was indistinguishable from controls (Fig EV3G and H).

Finally, we generated TFEB β-cell-specific/TFE3 double-KO mice (β-DKO mice) by crossing INS1-CRE mice with TFEB$^{fl/fl}$ (Settembre *et al*, 2012) and TFE3 full KO mice (Steingrimsson *et al*, 2002). Consistent with *in vitro* data, β-DKO mice failed to suppress *Ins2* mRNA upon overnight starvation and showed elevated *Ins2* levels upon refeeding, as demonstrated by RNA scope analysis in pancreatic sections (Fig 5A). No differences were detected in islet area (Fig 5B), β-cell mass, and β-cell maturation, as demonstrated by UCN3 staining (Fig 5C). Despite the striking increase in *Ins2* mRNA, β-DKO mice were characterized by lower body weight (Fig 5D) and glucose intolerance (Fig 5E) compared to control littermates, likely as a result of defective insulin release and consistent with recently published data (Park *et al*, 2022). Together these data suggest that TFEB and TFE3 act, at multiple levels, as crucial mediators of pancreatic β-cell function and metabolic homeostasis.

Discussion

Considerable work in the past years has tremendously advanced our knowledge of the mechanisms regulating insulin granule maturation and secretion as an acute response to increased nutrient levels. Previous studies have shown that mTORC1 activity is crucial for maintaining pancreatic β-cell homeostasis by modulating insulin mRNA translation and β-cell mass (Rachdi *et al*, 2008; Mori *et al*, 2009; Blandino-Rosano *et al*, 2017). However, the mechanisms governing insulin transcriptional regulation in response to variations in nutrient availability have remained less understood. In the present manuscript, we define a nutrient-controlled transcriptional mechanism that regulates insulin production to fine-tune glucose homeostasis at both cellular and organismal levels. Over the years, TFEB and TFE3 have emerged as important metabolic regulators in the liver and muscles, impacting body weight and glucose homeostasis (Settembre *et al*, 2013; Mansueto *et al*, 2017; Pastore *et al*, 2017). Here, we identify TFEB and TFE3 as key modulators of pancreatic β--cell function and further highlight the importance of these transcription factors in overall organismal metabolic regulation. As previously described in other cell types, TFEB and TFE3 are activated under scarce nutrient conditions and mTORC1 inhibition. In β-cells, fasting is associated with insulin downregulation (Rabinovitch *et al*, 1976; Zawalich *et al*, 1979; Giddings *et al*, 1981; Iwashima *et al*, 1994; Boland *et al*, 2018) to prevent undesired insulin secretion and further glycemic decrease in a hypoglycemic state. Our data show that TFEB and TFE3 mediate this process by suppressing insulin transcription and thus controlling glucose homeostasis. Accordingly, *INS* gene expression is severely suppressed both *in vitro* and *in vivo* upon acute and constitutive TFEB overexpression, respectively. Moreover, the physiological suppression of insulin transcription that occurs upon nutrient starvation in β-cells is severely impaired in TFEB/TFE3-depleted cells and mice. Although our *in vitro* studies were mainly focused on TFEB/TFE3-mediated control of insulin gene expression during amino acid starvation, we also observed that glucose deprivation promotes TFEB nuclear translocation in pancreatic beta cells, thus indicating that TFEB and TFE3 are main physiological regulators of insulin transcription in response to variations in the availability of different nutrients. Our data also suggest that mTORC1 activity in β-cells controls both acute and sustained responses to circulating nutrients, by regulating not only insulin mRNA translation, as previously shown (Blandino-Rosano *et al*, 2017), but also by controlling INS transcription via TFEB and TFE3 transcription factors. Surprisingly, however, TFEB/TFE3-deficient mice, despite showing a massive increase in INS mRNA levels, do not show hypoglycemia but instead are glucose intolerant, suggesting that lack of TFEB and TFE3 may affect β-cell function and insulin production at different levels.

Our findings also highlight a potential function of these transcription factors as negative transcriptional modulators. The evidence shown here that TFEB binds to enhancer cluster regions flanking the *INS* locus,

 © 2023 The Authors

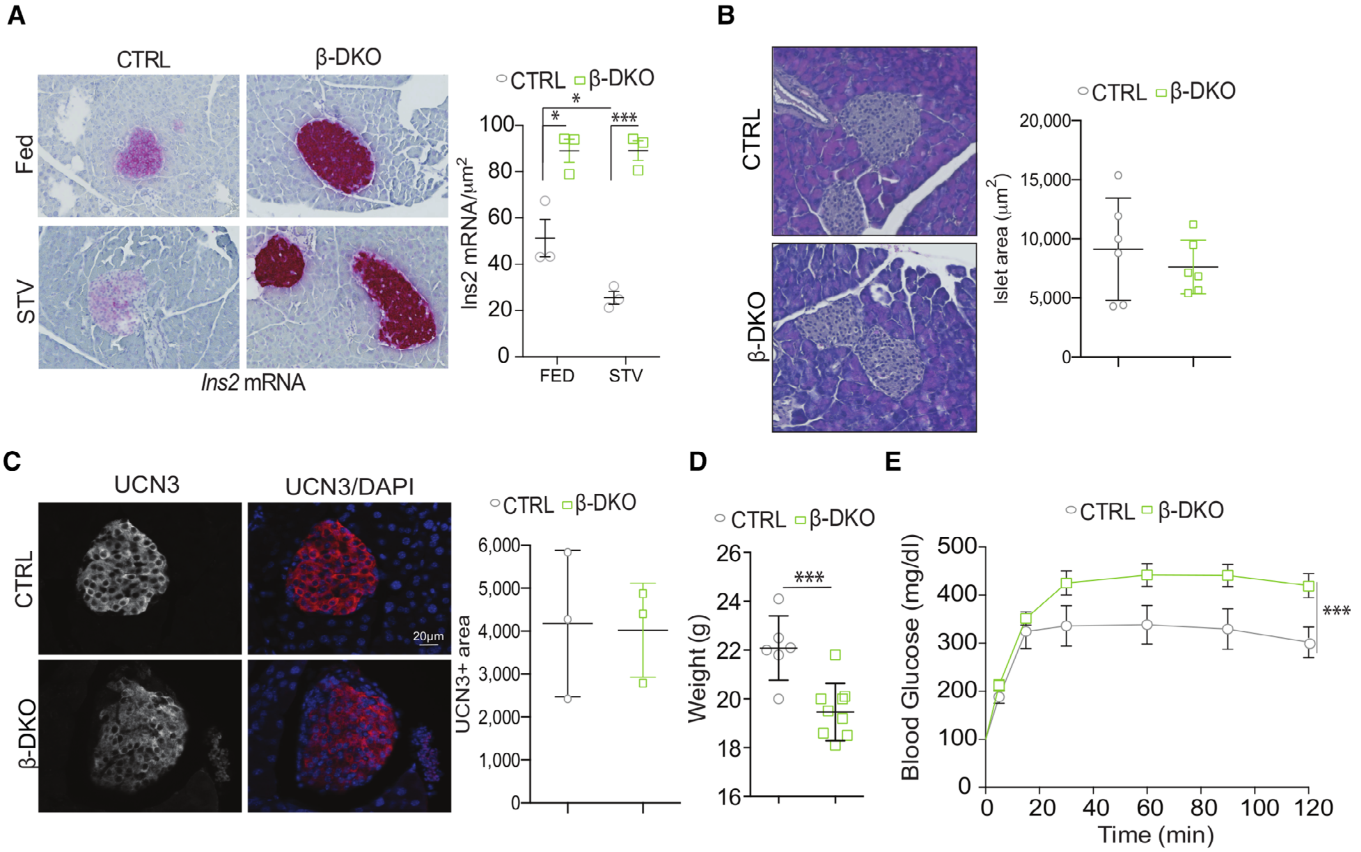

Figure 5. Beta-cell-specific deletion of TFEB and TFE3 results in enhanced insulin transcription and impaired glucose homeostasis.

A Representative images of RNA scope analysis for *Ins2* mRNA in pancreas from control and β-DKO mice in fed and starved conditions with relative quantification (n = 3/group). Each dot represents one mouse. Data are represented as mean ± standard error. Student's two-tailed t-test: **P*-value < 0.05; ****P*-value < 0.001.

B Representative images of pancreas slides from control and β-DKO mice stained with hematoxylin/eosin and quantification of islet area (n = 6/group; n = 11–25 islets per mouse). Each dot represents one mouse.

C Representative immunofluorescence images for UCN3 and quantification of the UCN3$^+$ area per islet in control and β β-DKO pancreas (n = 3/group; n = 8–25 islets per mouse). Each dot represents one mouse. Data are represented as mean ± standard error. Student's two-tailed t-test: ****P*-value < 0.001.

D Body weight of control and β-DKO mice at 8 weeks of age.

E Glucose tolerance test (GTT) of control and β-DKO mice. Each dot represents one mouse (n = 8/group). Two-way ANOVA: ****P*-value < 0.001.

Source data are available online for this figure.

previously proposed to act as a super-enhancer (Miguel-Escalada *et al*, 2019), suggests that TFEB and TFE3 may directly suppress *INS* transcription via super-enhancer binding. Interestingly, a previously reported motif-based binding prediction analysis identified TFEB as part of a core regulatory circuit (CRC) controlling β-cell function via super-enhancer binding (Alvarez-Dominguez *et al*, 2020). However, at this stage, we cannot exclude that TFEB and TFE3 instead repress the transcription of insulin and other β-specific genes in an indirect manner. For instance, consistent with a previous study (Carey *et al*, 2020), we found that TFEB promotes the transcription of *BHLHE40* and *BHLHE41* (Fig EV2E), two important transcriptional repressors which have been recently shown to suppress insulin gene expression via transcriptional inhibition of *INS* master regulators PDX1 and MafA (Tsuyama *et al*, 2023). Interestingly, TFEB, BHLHE40, and BHLHE41 have been shown to play a role in circadian rhythm regulation (Honma *et al*, 2002; Kato *et al*, 2014; Brooks & Dang, 2019; Pastore *et al*, 2019) which is central for β-cell function (Marcheva *et al*, 2010; Perelis *et al*, 2015; Alvarez-Dominguez *et al*, 2020). Whether TFEB, BHLHE40, and BHLHE41 are part of a transcriptional cascade that results in insulin gene regulation remains to be established in future studies.

Dysregulation of glucose homeostasis and β-cell dysfunction is a hallmark of human diseases, such as diabetes. Our study supports the key role of TFEB and TFE3 in pancreatic β-cell regulation and uncovers a new mechanism by which these transcription factors modulate organismal metabolism. These data may have important clinical implications in diabetes and other metabolic diseases.

Materials and Methods

Materials

Reagents used in this study were obtained from the following sources: antibodies to phospho-p70 S6 kinase (Thr389) (1A5) (Cat# 9206),

p70 S6 kinase (Cat# 9202), Rheb1 (Cat# 13789), 4E-BP1 (Cat# 9644), phospho-4E-BP1 (Ser65) (Cat# 9456), human TFEB (Cat# 4240), and insulin (Cat# 4590) were from Cell Signaling Technology; antibodies to GAPDH (6C5) (Cat# sc-32233) and LAMP1 (Cat# Sc20011) were from Santa Cruz; antibodies to TFE3 (Cat# HPA023881), glucagon (Cat#G2654), and V5 (Cat # V8137) were from Sigma Aldrich; antibody to TFEB (Cat# A303-673A) was from Bethyl; antibody to HA.11 Epitope Tag (Cat# 901513) was from Biolegend; and the antibody for UCN3 was from Phoenic Pharmaceutical (Cat#H-019029).

Plasmids: pRK5-HA GST RagA-Q66L (#19300) and pRK5-HA GST RagC-S75L (#19305) were a kind gift from David Sabatini (Addgene plasmids).

Chemicals: Torin 1 (Cat# 4247) was from Tocris; Protease Inhibitor Cocktail (Cat# P8340) was from Sigma Aldrich; and PhosSTOP phosphatase inhibitor cocktail tablets (Cat# 04906837001) were from Roche.

Cell culture

Human EndoC-βH1 cells were obtained from Univercell® company and maintained following the company instructions in a DMEM-derived medium. INS-1E cells were purchased from AddexBio (C0018009) and grown in RPMI 1640 supplemented with 10% FCS, 10 mM Hepes, 2 mM glutamine, 0.05 mM β-mercapto-ethanol, 1 mM PyrNa, and 1× penicillin–streptomycin solution. For experiments involving amino acid starvation, cells were rinsed twice with PBS and incubated in amino acid-free RPMI (Cat# R9010-01, USBiological) supplemented with 10% dialyzed FBS. Where indicated, cells were re-stimulated with 1× water-solubilized mix of essential (Cat#11130036, Thermo Fisher Scientific) and non-essential (Cat# 11140035, Thermo Fisher Scientific) amino acids re-suspended in amino acid-free RPMI. For glucose starvation, cells were rinsed twice with PBS and incubated with cell culture medium prepared in a DMEM w/o glucose (Cat# 11966025, Thermo Fisher Scientific). Glucose re-feeding was obtained by replacing glucose starvation medium with full-nutrient cell culture medium. For silencing experiments, cells were transfected using the RNAi Max® reagent from Invitrogen following the kit instructions and incubated for 72 h. The following siRNAs were used in this study: ON-Target plus® SMART pool Human TFE3 #L-009363-00-0020, ON-Target plus® SMART pool Human TFEB #L-009798-00-0020, and ON-Target plus® SMART pool Non-targeting control #D-001810-10-20.

CRISPR Cas9 gene editing of the INS-1E cells

For KO clone generation in INS-1E cells, wild-type cells were transfected with the Sigma CRISPr plasmids carrying simultaneously the gRNA and Cas9 (TFEB gRNA: GCTGCCATGGCGTCGCGCATCGG, TFE3 gRNA: CCGGCGAGCTTGCTGCAAG).

Single clones were screened for desired editing by sequencing (TFEB sequencing primers: FW 5′-ACTGAAGGACAGAGTCTTCACC, RV 5′-GACAGAAGAGGCAGAGGCCTTA; TFE3 sequencing primers: FW 5′-TGTGCCCCAGGTGTTTATGG, RV 5′-GCTACGGCCTCTTACC TCCT) and immunoblot. At least two clones per condition were tested in parallel for control and edited cell lines.

Viral infection

Retroviral plasmids encoding TFEB-3X-FLAG (Settembre *et al*, 2012) were co-transfected with pCMVgag/pol and CMV VSV-G packaging plasmids into actively growing HEK-293T cells using FuGENE 6 transfection reagent. Virus-containing supernatants were collected 48 h after transfection, diluted 1:2, and used to infect one six-well plate of INS1-E cells in presence of 8 μg/ml polybrene. Cells were analyzed 3 days after infection.

EndoC-BH1 were infected with lentivirus for TFEB-V5 and control luciferase expression using the Lenti-X™ Tet-One™ Inducible Expression System (Puro) from Takara.

High-content analysis of TFEB subcellular localization

EndoC-βH1 cells were seeded in 96-well plates and incubated for 24 h. Cells were then treated as indicated, rinsed with PBS once, fixed for 10 min with 4% paraformaldehyde, and stained with TFEB antibodies (Cat# 4240, Cell signaling) and DAPI. For the acquisition of the images, at least 10 fields were acquired per well of the 96-well plate by using confocal automated microscopy (Opera High Content System; Perkin-Elmer). A dedicated script (Medina *et al*, 2015) was used for analysis of TFEB localization on the different images (Harmony and Acapella software; Perkin-Elmer). The script calculates the ratio value resulting from the average intensity of nuclear TFEB fluorescence divided by the average of the cytosolic intensity of TFEB fluorescence. *P*-values were calculated on the basis of mean values from independent wells.

RNA extraction and RT–PCR

RNA extraction was performed using the Qiagen RNeasy® kit, following the kit instructions.

cDNA was generated from extracted RNA using the Qiagen QuantiTect Reverse Transcription Kit following the kit instructions. RT–PCR was done using the LightCycler® 480 SYBR Green I Master following the kit instructions using the LightCycler 480 II detection system (Roche). For expression studies, the qRT–PCR results were normalized against an internal control (HPRT). Primers used are listed in Table EV1.

3′mRNA sequencing library preparation, data processing, and analysis

Total RNA was quantified using the Qubit 2.0 fluorimetric Assay (Thermo Fisher Scientific). For RNA-seq analysis, library preparation was performed with a total of 100 ng of RNA from each sample using QuantSeq 3′mRNA-Seq Library prep kit (Lexogen) according to manufacturer's instructions. Amplified fragmented cDNA of 300 bp in size was sequenced in single-end mode by NovaSeq 6000 (Illumina) with a read length of 100 bp. Illumina NovaSeq 6000 base call (BCL) files were converted into fastq file through bcl2fastq. Sequence reads were trimmed BBDuk (sourceforge.net/projects/bbmap/) to remove adapter sequences and low-quality end bases ($Q < 20$). Alignment was performed with STAR 2.6.0a (Dobin *et al*, 2013) on the Hg38 reference provided by UCSC Genome Browser (Lee *et al*, 2020). Alignment to mm10, and rn6 reference genome assembly (Dobin *et al*, 2013), and counting by gene (Anders

© 2023 The Authors

et al, 2015) has been performed by using Ensembl assembly (release 93). Gene expression levels were determined with HTseq-count 0.9.1 (Anders *et al*, 2015). Differential expression analyses were performed using edgeR (Robinson *et al*, 2010), a statistical package based on generalized linear models, suitable for multifactorial experiments. The threshold for statistical significance chosen was false discovery rate (FDR) < 0.05. Gene ontology (GOEA) and functional annotation clustering analyses were performed on the induced and inhibited differentially expressed genes (DEGs) obtained in each RNA-sequencing dataset by using DAVID Bioinformatic Resources (Huang da *et al*, 2009a,b) restricting the output to Biological Process terms (BP_FAT). The "Kyoto Encyclopedia of Genes and Genomes" (KEGG Pathway) analyses were also performed (Kanehisa & Goto, 2000; Kanehisa *et al*, 2012). The threshold for statistical significance of GOEA was FDR < 0.1 and enrichment score ≥ 1.5, while for the KEGG pathway analyses was FDR < 0.1.

Heatmap and Venn diagrams were generated using custom annotation scripts.

List of abbreviations: FDR, false discovery rate; DEGs, differentially expressed genes.

Immunoblot assay

Protein lysates were extracted in RIPA buffer (Thermo Fisher) supplemented with the phosphatase inhibitor PhosSTOP *EASYpack* (Roche #04906837001) and the protease inhibitor cocktail (Sigma #P8340). Lysates were centrifuged for 10 min at 14,000 *g* at 4°C and supernatant conserved. Protein concentration was measured by Bradford assay and 50 µg of protein lysates were loaded for each sample, supplemented with Laemmle buffer. The lysates were run on precast NuPAGE™ 4–12% Bis-Tris Gel (Invitrogen #NP0335BOX [10 wells] or #NP0336BOX [15 wells]) inside the Xcell SureLock Mini cells and MOPES running buffer. Proteins were wet transferred for 1 h 30 min at 100 V on Immobilon® membrane (#IPVH00010) using the Bio-Rad® Mini Trans- blot® Cell. Membranes were subsequently incubated in 5% skim-milk TBS-T blocking solution for 1 h at room temperature. Incubation in the TBS-T 5% BSA primary antibody-containing solution was performed overnight at 4°C. After three TBS-T washes, incubation in the TBS-T 5% BSA secondary antibody-containing solution was performed for 1 h at room temperature, followed by three TBS-T washes. Membranes were developed using the ECL solution from Cyanagen ηC Ultra 2.0 ECL #XLS075L. Pictures were acquired with the GE® Amersham Imager 600, the UVITEC Q9 Mini Alliance, or UVITEC Mini HD9.

Immunofluorescence assay

PhenoPlate 96 wells (Perkin Elmer) were coated and then seeded with EndoC-BH1 cells at a density of 25,000 cells/well. Following treatment, cells were fixed in 4% PFA for 10 min then washed with PBS and permeabilized with PBS 0.02% Triton-X solution for 7 min. A blocking step was then performed using the blocking buffer (PBS, 3% BSA, 0.05% Saponin, 1% horse serum, and 50 mM NH_4Cl). Following the blocking steps, primary and secondary antibody incubations were performed in blocking buffer solution. Nuclei were stained with DAPI.

Histological analysis

H&E staining was performed following the IHC World protocol. Bright-field sections were scanned with ZEISS Axio Scan.Z1.

Immunofluorescence analyses were performed in 6 µm paraffin sections with VENTANA BenchMark Ultra automated staining instrument (Ventana Medical Systems, Roche), using VENTANA reagents except as noted, according to manufacturer's instructions. Slides were deparaffinized using EZ Prep solution (cat # 950-102) for 16 min at 72°C. Epitope retrieval was accomplished with CC1 solution (cat # 950-224) at a high temperature (95°C) for a period that is suitable for a specific tissue type. Antibodies were titered with a blocking solution into user-fillable dispensers for use on the automated stainer. Slides were developed using DISCOVERY FAM Kit (cat # 760-243) and DISCOVERY Red 610 Kit (cat #760-245, Roche) for 8 min. Slides were then counterstained with DISCOVERY QD DAPI (cat # 760-4196) for 8 min. Fluorescent sections were acquired using ZEISS microscope.

Automated RNA detection (RNA scope)

Insulin mRNA expression was determined using ISH with the fully automated RNA-scope assay on the VENTANA BenchMark Ultra (Ventana Medical Systems, Roche) platform. Sections were deparaffinized on the instrument, followed by target retrieval (16 min at 97°C) and protease treatment (16 min at 37°C). Probes (RNAscope® 2.5 VS Probe- Mm-Ins2-O1 #497819, ACD) were then hybridized at 43°C followed by RNAscope amplification (RNAscope® VS Universal AP Reagent Kit #323250, ACD) and red chromogenic detection using VS detection reagents (DISCOVERY mRNA Red Detection Kit #07099037001, Roche). Slides were then counterstained with hematoxylin II (cat # 790-2208) for 8 min, followed by Bluing reagent (cat # 760-2037) for 4 min. Bright-field sections were scanned with ZEISS Axio Scan.Z1. The whole digital slides were viewed by zen blue software. Antibodies used: TFEB (Bethyl A303-673A). Quantitative analysis of Insulin mRNA transcripts was performed using QuPath software.

Chromatin immunoprecipitation, library preparation, and sequencing

EndoC-βH1 cells were washed twice using PBS Ca^{2+}/Mg^{2+} free, and HBSS (14025092, Gibco) supplemented with HEPES (H0887, Euroclone) was added for 6 h to perform the starvation treatment. Then, 15×10^6 cells were fixed with 1% formaldehyde for 15 min at room temperature and subsequently quenched using glycine 0.1 M. Cell lysis, nuclear extraction, and sample preparation were performed as previously described (Cesana *et al*, 2018). Chromatin sonication was performed using the Branson Ultrasonics™ Sonifier™ SFX150. Immunoprecipitation was performed using 20 µl per sample of TFEB antibody on two independent replicates. Libraries were prepared from 10 ng of DNA using the NEBNext UltraTM II DNA Library Prep Kit for Illumina (New England Biolabs) of each replicate and the corresponding input whole cellular extract. The quality of libraries was assessed using Bioanalyzer DNA Analysis (Agilent Technologies) and quantified using Qubit 4 Fluorometer (Thermo Fisher Scientific). Libraries were sequenced on a NovaSeq 6000 sequencing system using a paired-end (PE) 100 cycles flow cell

© 2023 The Authors

(Illumina Inc.). Paired sequencing reads were aligned on human hg38 reference genome using BWA (Li & Durbin, 2009) and filtered with samtools (Danecek *et al*, 2021) to remove unmapped read pairs, not primary alignments, reads failing platform quality, with mapping quality score below 30, and duplicate reads were then removed using picard MarkDuplicates [http://broadinstitute.github.io/picard].

Each sample (IP or input) was equally split by randomly assigning half of the read pairs to each of two pseudo-replicates, the two biological replicates were pooled, and pools were split into two pseudo-replicates as well. Irreproducible discovery rate analysis [IDR] (Li *et al*, 2011) was applied as described in the ENCODE guidelines (Landt *et al*, 2012). Peaks were called for each IP/input pair using MACS2 (Zhang *et al*, 2008) with a threshold $P < 0.1$, then IDR was performed using the P-value as sorting column and an IDR score threshold of 0.01 on each of the following trio: (1) biological replicate 1 versus its own pseudo-replicates; (2) biological replicate 2 versus its own pseudo-replicates; (3) pool versus its own pseudo-replicates; and (4) pool versus biological replicate 1 and biological replicate 2. As final peak selection, we merged the coordinates of peaks called (1), (2), and (4) and only kept those supported by at least two of the three analyses.

Mouse maintenance

All procedures on mice were approved by the Italian Ministry of Health. Mice were housed at the TIGEM animal house under SPF certification. To generate mice, we bred the INS1Cre mice from the Thorens laboratory (B6(Cg)-Ins1tm1.1(cre)Thor/J), with TFEB-overexpressing mice (Settembre *et al*, 2011). For the generation of double-KO mice, INS1Cre mice were crossed with TFEB conditional KO mice (Settembre *et al*, 2012) and TFE3 KO mice (Steingrimsson *et al*, 2002).

Mouse metabolic phenotyping

All metabolic tests were performed in age-matched, sex-matched cohorts. All mice were bred on a pure C57Bl6/J genetic background. Glucose tolerance test (GTT) was performed by intraperitoneally injecting 2 g of glucose/kg body mass of an aqueous 20% glucose solution to overnight fasted mice. Prior to injection, fasting glycemia was measured using the Wellion® LUNA glucometer with the corresponding Wellion® LUNA test stripes GLU by collecting a blood drop from the tail. Glycemia was measured the same way at 15, 30, 60, 90, and 120 min after glucose injection. When applying, blood sampling from the tail for glucose-stimulated insulin secretion (GSIS) was performed using Sarstedt Microvette CB300. Insulin tolerance test was performed in a similar way as the GTT, injecting 0.75 IU/kg body mass in 6 h fasted mice.

Statistical analysis

Data are expressed as mean ± standard error. Statistical significance was calculated using the Student's two-tailed t-test or two-way ANOVA as indicated in figure legends. A P-value < 0.05 was considered statistically significant.

Data availability

All the RNA-seq datasets discussed in this work were deposited in GEO repository (Barrett *et al*, 2011).

The titles of the dataset are as follows: "Transcriptome profile of INS-1E Ctrl and hTFEB OE cells", (GSE155221); "Transcriptome profile of ENDOC-BH1 cells silencing TFEB and TFE3 (siTFEB/TFE3) or controls", (GSE155220); "Transcriptome profile of INS-1E Ctrl and DKO cells", (GSE153709); "Transcriptome profile of ENDOC-BH1 cells overexpressing TFEB-V5 or controls", (GSE154059); and "Transcriptome profile of isolated murine islets overexpressing TFEB or controls" (GSE154062). The SuperSeries GSE154063 whose title is "Transcriptome profile of ENDOC-BH1 cells and isolated murine islets modulating TFEB expression" includes the three datasets GSE153709, GSE154059, and GSE154062.

Expanded View for this article is available online.

Acknowledgements

We thank Roberto Zoncu, Nicola Brunetti-Pierri, and Carmine Settembre for critical reading of the manuscript. We also thank Chiara Soldati and Diego Medina of the high content screening facility (HCS) for the analysis of TFEB subcellular localization in β-cells, and the Advanced Histopathology Facility of TIGEM for tissue processing, RNAscope, and histological analyses. We thank Valerio Benedetti and Alessandro Venuta for helping in the characterization of the EndoC-BH1 cells. We thank Diego Carrella and Lucio Di Filippo of the TIGEM Bioinformatics core for the visualization of the heatmap. We also thank Jlenia Monfregola for drawing the graphical abstract, created with BioRender.com. We acknowledge the support of the following funding agencies: the Italian Telethon Foundation (to N.P., G.N., and A.B.); Fondazione AIRC per la ricercar sul cancro (MFAG-23538 to G.N.; MFAG-24872 to N.P.; IG-22103 and 5x1000-21051 to A.B.); Fondation pour la Recherche Médicale (Post-Doctorat à l'étranger to A.P.); the European Foundation for the Study of Diabetes (EFSD/Lilly Young Investigator Research Award Program and EFSD Albert Renold Travel Fellowship program to A.P.); MIUR (PRIN 2017YF9FBS and 2022CRFNCP to G.N.; PRIN 2017E5L5P3 to A.B.; PRIN P2022T4PKT to G.N. and A.B.); Wereld Kanker Onderzoek Fonds (WKOF), as part of the World Cancer Research Fund International grant program (IIG_FULL_2022_009 to G.N.); and the European Research Council H2020 AdG (LYSOSOMICS 694282 to A.B.).

Author contributions

Adrien Pasquier: Conceptualization; formal analysis; investigation; visualization; methodology; writing – original draft. **Nunzia Pastore:** Conceptualization; formal analysis; funding acquisition; validation; investigation; visualization; methodology; writing – original draft; writing – review and editing. **Luca D'Orsi:** Investigation; methodology. **Rita Colonna:** Validation; investigation. **Alessandra Esposito:** Validation; investigation; visualization. **Veronica Maffia:** Validation; investigation. **Rossella De Cegli:** Data curation; formal analysis; visualization. **Margherita Mutarelli:** Data curation; formal analysis. **Susanna Ambrosio:** Investigation. **Gennaro Tufano:** Investigation. **Antonio Grimaldi:** Data curation; formal analysis. **Marcella Cesana:** Investigation; methodology. **Davide Cacchiarelli:** Data curation; formal analysis. **Nathalie Delalleau:** Resources. **Gennaro Napolitano:** Conceptualization; resources; supervision; funding acquisition; writing – original draft; writing – review and editing. **Andrea Ballabio:** Conceptualization; resources; supervision; funding acquisition; writing – original draft; writing – review and editing.

 © 2023 The Authors

Disclosure and competing interests statement

A.B. is cofounder of CASMA Therapeutics, Inc., and Advisory board member of NexGeneration Diagnostics and Avilar Therapeutics.

References

Alvarez-Dominguez JR, Donaghey J, Rasouli N, Kenty JHR, Helman A, Charlton J, Straubhaar JR, Meissner A, Melton DA (2020) Circadian entrainment triggers maturation of human in vitro islets. *Cell Stem Cell* 26: 108 – 122.e10

Anders S, Pyl PT, Huber W (2015) HTSeq—a Python framework to work with high-throughput sequencing data. *Bioinformatics* 31: 166 – 169

Barrett T, Troup DB, Wilhite SE, Ledoux P, Evangelista C, Kim IF, Tomashevsky M, Marshall KA, Phillippy KH, Sherman PM *et al* (2011) NCBI GEO: archive for functional genomics data sets–10 years on. *Nucleic Acids Res* 39: D1005 – D1010

Blandino-Rosano M, Barbaresso R, Jimenez-Palomares M, Bozadjieva N, Werneck-de-Castro JP, Hatanaka M, Mirmira RG, Sonenberg N, Liu M, Ruegg MA *et al* (2017) Loss of mTORC1 signalling impairs beta-cell homeostasis and insulin processing. *Nat Commun* 8: 16014

Boland BB, Brown C Jr, Alarcon C, Demozay D, Grimsby JS, Rhodes CJ (2018) β-cell control of insulin production during starvation-refeeding in male rats. *Endocrinology* 159: 895 – 906

Brady OA, Jeong E, Martina JA, Pirooznia M, Tunc I, Puertollano R (2018) The transcription factors TFE3 and TFEB amplify p53 dependent transcriptional programs in response to DNA damage. *Elife* 7: e40856

Brooks RC, Dang CV (2019) Autophagy: clocking in for the night shift. *EMBO J* 38: e102434

Carey KL, Paulus GLC, Wang L, Balce DR, Luo JW, Bergman P, Ferder IC, Kong L, Renaud N, Singh S *et al* (2020) TFEB transcriptional responses reveal negative feedback by BHLHE40 and BHLHE41. *Cell Rep* 33: 108371

Castellano BM, Thelen AM, Moldavski O, Feltes M, van der Welle RE, Mydock-McGrane L, Jiang X, van Eijkeren RJ, Davis OB, Louie SM *et al* (2017) Lysosomal cholesterol activates mTORC1 via an SLC38A9-Niemann-Pick C1 signaling complex. *Science* 355: 1306 – 1311

Cesana M, Guo MH, Cacchiarelli D, Wahlster L, Barragan J, Doulatov S, Vo LT, Salvatori B, Trapnell C, Clement K *et al* (2018) A CLK3-HMGA2 alternative splicing axis impacts human hematopoietic stem cell molecular identity throughout development. *Cell Stem Cell* 22: 575 – 588.e7

Cnop M, Welsh N, Jonas JC, Jorns A, Lenzen S, Eizirik DL (2005) Mechanisms of pancreatic beta-cell death in type 1 and type 2 diabetes: many differences, few similarities. *Diabetes* 54: S97 – S107

Cui Z, Napolitano G, de Araujo MEG, Esposito A, Monfregola J, Huber LA, Ballabio A, Hurley JH (2023) Structure of the lysosomal mTORC1-TFEB-Rag-Ragulator megacomplex. *Nature* 614: 572 – 579

Danecek P, Bonfield JK, Liddle J, Marshall J, Ohan V, Pollard MO, Whitwham A, Keane T, McCarthy SA, Davies RM *et al* (2021) Twelve years of SAMtools and BCFtools. *Gigascience* 10: giab008

Dobin A, Davis CA, Schlesinger F, Drenkow J, Zaleski C, Jha S, Batut P, Chaisson M, Gingeras TR (2013) STAR: ultrafast universal RNA-seq aligner. *Bioinformatics* 29: 15 – 21

El-Houjeiri L, Possik E, Vijayaraghavan T, Paquette M, Martina JA, Kazan JM, Ma EH, Jones R, Blanchette P, Puertollano R *et al* (2019) The transcription factors TFEB and TFE3 link the FLCN-AMPK signaling axis to innate immune response and pathogen resistance. *Cell Rep* 26: 3613 – 3628.e6

Gambardella G, Staiano L, Moretti MN, De Cegli R, Fagnocchi L, Di Tullio G, Polletti S, Braccia C, Armirotti A, Zippo A *et al* (2020) GADD34 is a modulator of autophagy during starvation. *Sci Adv* 6: eabb0205

Giddings SJ, Chirgwin J, Permutt MA (1981) The effects of fasting and feeding on preproinsulin messenger RNA in rats. *J Clin Invest* 67: 952 – 960

Gloyn AL, Ibberson M, Marchetti P, Powers AC, Rorsman P, Sander M, Solimena M (2022) Every islet matters: improving the impact of human islet research. *Nat Metab* 4: 970 – 977

Honma S, Kawamoto T, Takagi Y, Fujimoto K, Sato F, Noshiro M, Kato Y, Honma K (2002) Dec1 and Dec2 are regulators of the mammalian molecular clock. *Nature* 419: 841 – 844

Huang da W, Sherman BT, Lempicki RA (2009a) Bioinformatics enrichment tools: paths toward the comprehensive functional analysis of large gene lists. *Nucleic Acids Res* 37: 1 – 13

Huang da W, Sherman BT, Lempicki RA (2009b) Systematic and integrative analysis of large gene lists using DAVID bioinformatics resources. *Nat Protoc* 4: 44 – 57

Iwashima Y, Kondoh-Abiko A, Seino S, Takeda J, Eto M, Polonsky KS, Makino I (1994) Reduced levels of messenger ribonucleic acid for calcium channel, glucose transporter-2, and glucokinase are associated with alterations in insulin secretion in fasted rats. *Endocrinology* 135: 1010 – 1017

Kanehisa M, Goto S (2000) KEGG: kyoto encyclopedia of genes and genomes. *Nucleic Acids Res* 28: 27 – 30

Kanehisa M, Goto S, Sato Y, Furumichi M, Tanabe M (2012) KEGG for integration and interpretation of large-scale molecular data sets. *Nucleic Acids Res* 40: D109 – D114

Kato Y, Kawamoto T, Fujimoto K, Noshiro M (2014) DEC1/STRA13/SHARP2 and DEC2/SHARP1 coordinate physiological processes, including circadian rhythms in response to environmental stimuli. *Curr Top Dev Biol* 110: 339 – 372

Landt SG, Marinov GK, Kundaje A, Kheradpour P, Pauli F, Batzoglou S, Bernstein BE, Bickel P, Brown JB, Cayting P *et al* (2012) ChIP-seq guidelines and practices of the ENCODE and modENCODE consortia. *Genome Res* 22: 1813 – 1831

Lawlor N, Marquez EJ, Orchard P, Narisu N, Shamim MS, Thibodeau A, Varshney A, Kursawe R, Erdos MR, Kanke M *et al* (2019) Multiomic profiling identifies cis-regulatory networks underlying human pancreatic beta cell identity and function. *Cell Rep* 26: 788 – 801.e6

Lee CM, Barber GP, Casper J, Clawson H, Diekhans M, Gonzalez JN, Hinrichs AS, Lee BT, Nassar LR, Powell CC *et al* (2020) UCSC Genome Browser enters 20th year. *Nucleic Acids Res* 48: D756 – D761

Li H, Durbin R (2009) Fast and accurate short read alignment with Burrows-Wheeler transform. *Bioinformatics* 25: 1754 – 1760

Li Q, Brown JB, Huang H, Bickel PJ (2011) Measuring reproducibility of high-throughput experiments. *Ann Appl Stat* 5: 1752 – 1779

Li L, Friedrichsen HJ, Andrews S, Picaud S, Volpon L, Ngeow K, Berridge G, Fischer R, Borden KLB, Filippakopoulos P *et al* (2018) A TFEB nuclear export signal integrates amino acid supply and glucose availability. *Nat Commun* 9: 2685

Li K, Wada S, Gosis BS, Thorsheim C, Loose P, Arany Z (2022) Folliculin promotes substrate-selective mTORC1 activity by activating RagC to recruit TFE3. *PLoS Biol* 20: e3001594

Liu GY, Sabatini DM (2020) mTOR at the nexus of nutrition, growth, ageing and disease. *Nat Rev Mol Cell Biol* 21: 183 – 203

Mansueto G, Armani A, Viscomi C, D'Orsi L, De Cegli R, Polishchuk EV, Lamperti C, Di Meo I, Romanello V, Marchet S *et al* (2017) Transcription factor EB controls metabolic flexibility during exercise. *Cell Metab* 25: 182 – 196

Marcheva B, Ramsey KM, Buhr ED, Kobayashi Y, Su H, Ko CH, Ivanova G, Omura C, Mo S, Vitaterna MH *et al* (2010) Disruption of the clock components CLOCK and BMAL1 leads to hypoinsulinaemia and diabetes. *Nature* 466: 627 – 631

© 2023 The Authors

Martina JA, Puertollano R (2013) Rag GTPases mediate amino acid-dependent recruitment of TFEB and MITF to lysosomes. *J Cell Biol* 200: 475 – 491

Martina JA, Chen Y, Gucek M, Puertollano R (2012) MTORC1 functions as a transcriptional regulator of autophagy by preventing nuclear transport of TFEB. *Autophagy* 8: 903 – 914

Martina JA, Diab HI, Lishu L, Jeong AL, Patange S, Raben N, Puertollano R (2014) The nutrient-responsive transcription factor TFE3 promotes autophagy, lysosomal biogenesis, and clearance of cellular debris. *Sci Signal* 7: ra9

Medina DL, Di Paola S, Peluso I, Armani A, De Stefani D, Venditti R, Montefusco S, Scotto-Rosato A, Prezioso C, Forrester A *et al* (2015) Lysosomal calcium signalling regulates autophagy through calcineurin and TFEB. *Nat Cell Biol* 17: 288 – 299

van der Meulen T, Xie R, Kelly OG, Vale WW, Sander M, Huising MO (2012) Urocortin 3 marks mature human primary and embryonic stem cell-derived pancreatic alpha and beta cells. *PLoS One* 7: e52181

Miguel-Escalada I, Bonas-Guarch S, Cebola I, Ponsa-Cobas J, Mendieta-Esteban J, Atla G, Javierre BM, Rolando DMY, Farabella I, Morgan CC *et al* (2019) Human pancreatic islet three-dimensional chromatin architecture provides insights into the genetics of type 2 diabetes. *Nat Genet* 51: 1137 – 1148

Mori H, Inoki K, Opland D, Munzberg H, Villanueva EC, Faouzi M, Ikenoue T, Kwiatkowski DJ, Macdougald OA, Myers MG Jr *et al* (2009) Critical roles for the TSC-mTOR pathway in beta-cell function. *Am J Physiol Endocrinol Metab* 297: E1013 – E1022

Mularoni L, Ramos-Rodriguez M, Pasquali L (2017) The pancreatic Islet Regulome Browser. *Front Genet* 8: 13

Napolitano G, Ballabio A (2016) TFEB at a glance. *J Cell Sci* 129: 2475 – 2481

Napolitano G, Esposito A, Choi H, Matarese M, Benedetti V, Di Malta C, Monfregola J, Medina DL, Lippincott-Schwartz J, Ballabio A (2018) mTOR-dependent phosphorylation controls TFEB nuclear export. *Nat Commun* 9: 3312

Napolitano G, Di Malta C, Esposito A, de Araujo MEG, Pece S, Bertalot G, Matarese M, Benedetti V, Zampelli A, Stasyk T *et al* (2020) A substrate-specific mTORC1 pathway underlies Birt-Hogg-Dube syndrome. *Nature* 585: 597 – 602

Napolitano G, Di Malta C, Ballabio A (2022) Non-canonical mTORC1 signaling at the lysosome. *Trends Cell Biol* 32: 920 – 931

Orozco JM, Krawczyk PA, Scaria SM, Cangelosi AL, Chan SH, Kunchok T, Lewis CA, Sabatini DM (2020) Dihydroxyacetone phosphate signals glucose availability to mTORC1. *Nat Metab* 2: 893 – 901

Palmieri M, Impey S, Kang H, di Ronza A, Pelz C, Sardiello M, Ballabio A (2011) Characterization of the CLEAR network reveals an integrated control of cellular clearance pathways. *Hum Mol Genet* 20: 3852 – 3866

Park K, Lim H, Kim J, Hwang Y, Lee YS, Bae SH, Kim H, Kim H, Kang SW, Kim JY *et al* (2022) Lysosomal Ca(2+)-mediated TFEB activation modulates mitophagy and functional adaptation of pancreatic beta-cells to metabolic stress. *Nat Commun* 13: 1300

Pastore N, Vainshtein A, Klisch TJ, Armani A, Huynh T, Herz NJ, Polishchuk EV, Sandri M, Ballabio A (2017) TFE3 regulates whole-body energy metabolism in cooperation with TFEB. *EMBO Mol Med* 9: 605 – 621

Pastore N, Vainshtein A, Herz NJ, Huynh T, Brunetti L, Klisch TJ, Mutarelli M, Annunziata P, Kinouchi K, Brunetti-Pierri N *et al* (2019) Nutrient-sensitive transcription factors TFEB and TFE3 couple autophagy and metabolism to the peripheral clock. *EMBO J* 38: e101347

Perelis M, Marcheva B, Ramsey KM, Schipma MJ, Hutchison AL, Taguchi A, Peek CB, Hong H, Huang W, Omura C *et al* (2015) Pancreatic beta cell enhancers regulate rhythmic transcription of genes controlling insulin secretion. *Science* 350: aac4250

Puertollano R, Ferguson SM, Brugarolas J, Ballabio A (2018) The complex relationship between TFEB transcription factor phosphorylation and subcellular localization. *EMBO J* 37: e98804

Rabinovitch A, Grill V, Renold AE, Cerasi E (1976) Insulin release and cyclic AMP accumulation in response to glucose in pancreatic islets of fed and starved rats. *J Clin Invest* 58: 1209 – 1216

Rachdi L, Balcazar N, Osorio-Duque F, Elghazi L, Weiss A, Gould A, Chang-Chen KJ, Gambello MJ, Bernal-Mizrachi E (2008) Disruption of Tsc2 in pancreatic beta cells induces beta cell mass expansion and improved glucose tolerance in a TORC1-dependent manner. *Proc Natl Acad Sci USA* 105: 9250 – 9255

Ravassard P, Hazhouz Y, Pechberty S, Bricout-Neveu E, Armanet M, Czernichow P, Scharfmann R (2011) A genetically engineered human pancreatic beta cell line exhibiting glucose-inducible insulin secretion. *J Clin Invest* 121: 3589 – 3597

Robinson MD, McCarthy DJ, Smyth GK (2010) edgeR: a Bioconductor package for differential expression analysis of digital gene expression data. *Bioinformatics* 26: 139 – 140

Roczniak-Ferguson A, Petit CS, Froehlich F, Qian S, Ky J, Angarola B, Walther TC, Ferguson SM (2012) The transcription factor TFEB links mTORC1 signaling to transcriptional control of lysosome homeostasis. *Sci Signal* 5: ra42

Sancak Y, Peterson TR, Shaul YD, Lindquist RA, Thoreen CC, Bar-Peled L, Sabatini DM (2008) The Rag GTPases bind raptor and mediate amino acid signaling to mTORC1. *Science* 320: 1496 – 1501

Sancak Y, Bar-Peled L, Zoncu R, Markhard AL, Nada S, Sabatini DM (2010) Ragulator-Rag complex targets mTORC1 to the lysosomal surface and is necessary for its activation by amino acids. *Cell* 141: 290 – 303

Sardiello M, Ballabio A (2009) Lysosomal enhancement: a CLEAR answer to cellular degradative needs. *Cell Cycle* 8: 4021 – 4022

Sardiello M, Palmieri M, di Ronza A, Medina DL, Valenza M, Gennarino VA, Di Malta C, Donaudy F, Embrione V, Polishchuk RS *et al* (2009) A gene network regulating lysosomal biogenesis and function. *Science* 325: 473 – 477

Settembre C, Di Malta C, Polito VA, Garcia Arencibia M, Vetrini F, Erdin S, Erdin SU, Huynh T, Medina D, Colella P *et al* (2011) TFEB links autophagy to lysosomal biogenesis. *Science* 332: 1429 – 1433

Settembre C, Zoncu R, Medina DL, Vetrini F, Erdin S, Erdin S, Huynh T, Ferron M, Karsenty G, Vellard MC *et al* (2012) A lysosome-to-nucleus signalling mechanism senses and regulates the lysosome via mTOR and TFEB. *EMBO J* 31: 1095 – 1108

Settembre C, De Cegli R, Mansueto G, Saha PK, Vetrini F, Visvikis O, Huynh T, Carissimo A, Palmer D, Klisch TJ *et al* (2013) TFEB controls cellular lipid metabolism through a starvation-induced autoregulatory loop. *Nat Cell Biol* 15: 647 – 658

Steingrimsson E, Tessarollo L, Pathak B, Hou L, Arnheiter H, Copeland NG, Jenkins NA (2002) Mitf and Tfe3, two members of the Mitf-Tfe family of bHLH-Zip transcription factors, have important but functionally redundant roles in osteoclast development. *Proc Natl Acad Sci USA* 99: 4477 – 4482

Thorens B, Tarussio D, Maestro MA, Rovira M, Heikkila E, Ferrer J (2015) Ins1 (Cre) knock-in mice for beta cell-specific gene recombination. *Diabetologia* 58: 558 – 565

Tsonkova VG, Sand FW, Wolf XA, Grunnet LG, Kirstine Ringgaard A, Ingvorsen C, Winkel L, Kalisz M, Dalgaard K, Bruun C *et al* (2018) The EndoC-betaH1 cell line is a valid model of human beta cells and applicable for screenings to identify novel drug target candidates. *Mol Metab* 8: 144 – 157

Tsuyama T, Sato Y, Yoshizawa T, Matsuoka T, Yamagata K (2023) Hypoxia causes pancreatic beta-cell dysfunction and impairs insulin secretion by activating the transcriptional repressor BHLHE40. *EMBO Rep* 24: e56227

 © 2023 The Authors

Vasiljevic J, Torkko JM, Knoch KP, Solimena M (2020) The making of insulin in health and disease. *Diabetologia* 63: 1981 – 1989

Yoon I, Nam M, Kim HK, Moon HS, Kim S, Jang J, Song JA, Jeong SJ, Kim SB, Cho S *et al* (2020) Glucose-dependent control of leucine metabolism by leucyl-tRNA synthetase 1. *Science* 367: 205 – 210

Zawalich WS, Dye ES, Pagliara AS, Rognstad R, Matschinsky FM (1979) Starvation diabetes in the rat: onset, recovery, and specificity of reduced responsiveness of pancreatic beta-cells. *Endocrinology* 104: 1344 – 1351

Zhang Y, Liu T, Meyer CA, Eeckhoute J, Johnson DS, Bernstein BE, Nusbaum C, Myers RM, Brown M, Li W *et al* (2008) Model-based analysis of ChIP-Seq (MACS). *Genome Biol* 9: R137

License: This is an open access article under the terms of the Creative Commons Attribution License, which permits use, distribution and reproduction in any medium, provided the original work is properly cited.

Expanded View Figures

Figure EV1. TFEB controls INS mRNA levels in INS-1E cells.

A Representative immunoblot of lysates from INS-1E cells infected with hTFEB-Flag-expressing lentivirus (TFEB-OE) or control (CTRL) cells.
B Principal component analysis plot of transcriptomic data from TFEB-OE INS1 cells.
C Gene ontology analysis for significantly upregulated (red) and downregulated (green) genes in TFEB-overexpressing (TFEB-OE) cells compared to control (CTRL) upon amino acid starvation (−aa) for 16 h.
D INS1 and INS2 mRNA levels from control (CTRL) or TFEB-overexpressing (TFEB-OE) INS1 cells incubated with full medium (FED) or upon amino acid starvation (−aa) for 16 h. Each dot represents one mouse (n = 3-4/group). Data are represented as mean ± standard error. Student's two-tailed t-test: ***P-value < 0.001.
E Representative immunoblot for TFEB and TFE3 in DKO cells. GAPDH was used as a loading control.
F Principal component analysis plot of transcriptomic data from TFEB/TFE3 DKO INS1 cells.
G Gene enrichment analysis for significantly upregulated (green) and downregulated (red) genes from RNA seq data of DKO cells in comparison to control cells upon amino acid starvation (−aa) for 16 h.
H INS1 and INS2 mRNA levels from CTRL or TFEB/TFE3-DKO INS1 cells incubated with full medium (FED) or upon amino acid starvation (−aa) for 16 h. Each dot represents one mouse (n = 3-4/group). Data are represented as mean ± standard error Student's two-tailed t-test: *P-value < 0.05; ***P-value < 0.001.
I Venn diagram showing the comparison of the datasets of TFEB-overexpressing (TFEB-OE) versus TFEB/3 DKO INS1 cells.
J Heatmap showing the 550 DEGs significantly regulated in opposite correlation in TFEB-OE versus TFEB/3 DKO INS1 cells. The lanes corresponding to insulin genes (Ins1 and Ins2) are indicated.

© 2023 The Authors

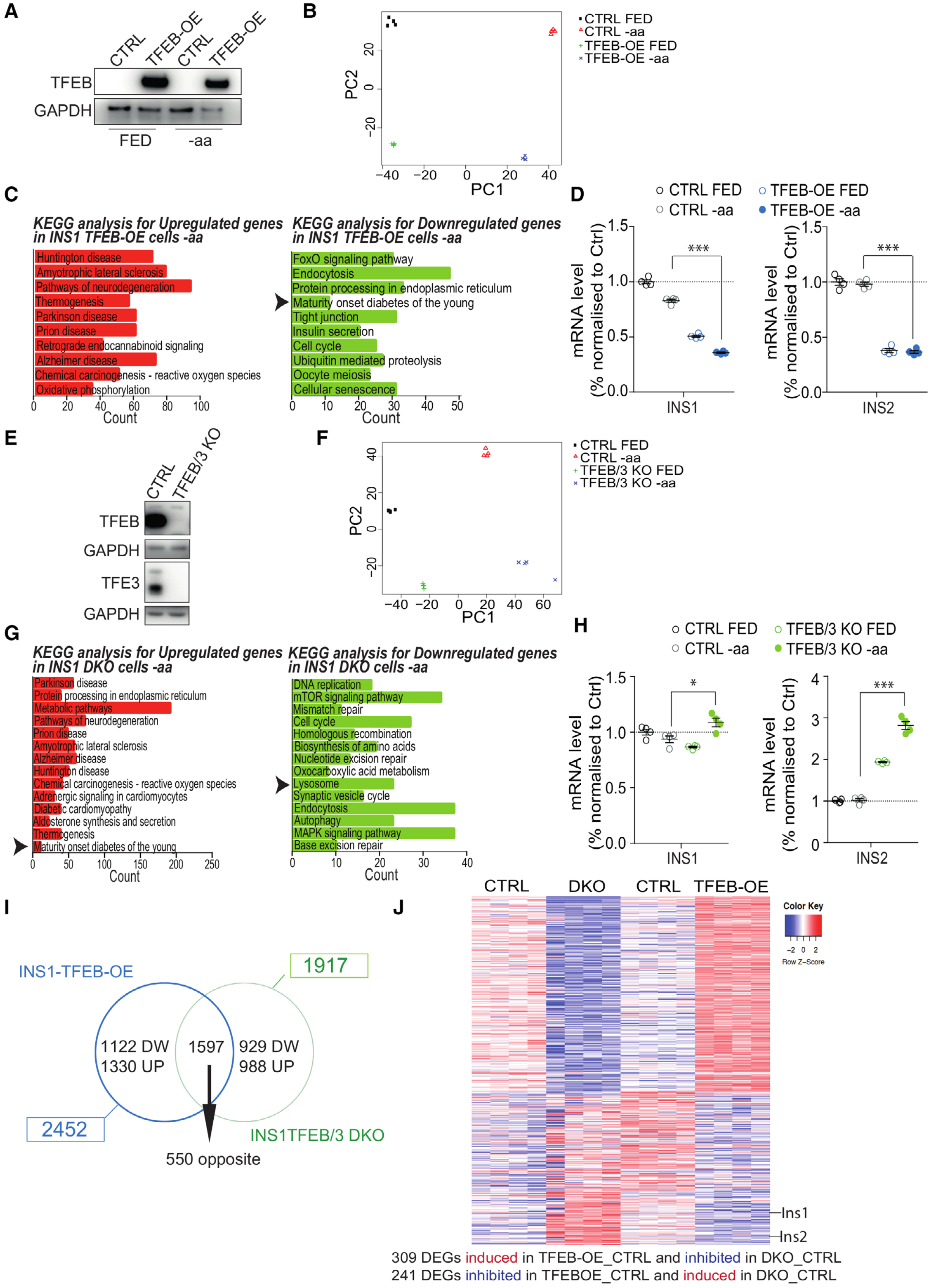
A
CTRL
TFEB-OE
CTRL
TFEB-OE
TFEB
GAPDH
FED
-aa
B
CTRL FED
CTRL -aa
TFEB-OE FED
TFEB-OE -aa
PC1
PC2
C
KEGG analysis for Upregulated genes in INS1 TFEB-OE cells -aa
Huntington disease
Amyotrophic lateral sclerosis
Pathways of neurodegeneration
Thermogenesis
Parkinson disease
Prion disease
Retrograde endocannabinoid signaling
Alzheimer disease
Chemical carcinogenesis - reactive oxygen species
Oxidative phosphorylation
Count
KEGG analysis for Downregulated genes in INS1 TFEB-OE cells -aa
FoxO signaling pathway
Endocytosis
Protein processing in endoplasmic reticulum
Maturity onset diabetes of the young
Tight junction
Insulin secretion
Cell cycle
Ubiquitin mediated proteolysis
Oocyte meiosis
Cellular senescence
Count
D
CTRL FED
CTRL -aa
TFEB-OE FED
TFEB-OE -aa
mRNA level (% normalised to Ctrl)
INS1
INS2
E
CTRL
TFEB/3 KO
TFEB
GAPDH
TFE3
GAPDH
F
CTRL FED
CTRL -aa
TFEB/3 KO FED
TFEB/3 KO -aa
PC1
PC2
G
KEGG analysis for Upregulated genes in INS1 DKO cells -aa
Parkinson disease
Protein processing in endoplasmic reticulum
Metabolic pathways
Pathways of neurodegeneration
Prion disease
Amyotrophic lateral sclerosis
Alzheimer disease
Huntington disease
Chemical carcinogenesis - reactive oxygen species
Adrenergic signaling in cardiomyocytes
Diabetic cardiomyopathy
Aldosterone synthesis and secretion
Thermogenesis
Maturity onset diabetes of the young
Count
KEGG analysis for Downregulated genes in INS1 DKO cells -aa
DNA replication
mTOR signaling pathway
Mismatch repair
Cell cycle
Homologous recombination
Biosynthesis of amino acids
Nucleotide excision repair
Oxocarboxylic acid metabolism
Lysosome
Synaptic vesicle cycle
Endocytosis
Autophagy
MAPK signaling pathway
Base excision repair
Count
H
CTRL FED
CTRL -aa
TFEB/3 KO FED
TFEB/3 KO -aa
mRNA level (% normalised to Ctrl)
INS1
INS2
I
INS1-TFEB-OE
1917
1122 DW
1330 UP
1597
929 DW
988 UP
2452
INS1TFEB/3 DKO
550 opposite
J
CTRL
DKO
CTRL
TFEB-OE
Color Key
Row Z-Score
Ins1
Ins2
309 DEGs induced in TFEB-OE_CTRL and inhibited in DKO_CTRL
241 DEGs inhibited in TFEBOE_CTRL and induced in DKO_CTRL

Figure EV1.

A

Lysosome	CD63, ASAH1, HEXB, HEXA, GBA, TCIRG1, NAGPA, GNS, SGSH, GGA2, LAPTM4B, CLN3, LAMP1, NAGLU, GM2A, CTSL, NEU1, PSAP, SLC17A5, ATP6V1H, ACP5, CTSF, CTSD, CTSB, CTSA, CD164, ATP6V0B, FUCA1, FUCA2, M6PR, CTNS, MCOLN1, IGF2R, SUMF1, GALNS, GNPTG, NPC2, PPT1, TPP1, ATP6V0D1, CD68, GLA
Autophagy	ATG3, GABARAPL2, UVRAG, ATG9B, RRAS2, IRS2, NRAS, TBK1, MRAS, LAMP1, RRAGC, RHEB, CTSL, BCL2, MAPK1, SMRC8, SQSTM1, CTSD, HRAS, SNAP29, CTSB, RAB7A
Oxidative Phosphorylation	ATP6V1G1, ATP6V0B, NDUFB8, ATP6V0E1, NDUFA1, COX7A2, TCIRG1, COX7C, COX5A, PPA1, UQCRQ, NDUFS6, ATP6V1H, CYC1, ATP6V1E1, ATP6V0D1, ATP6V1D, ATP6V1C1, ATP6V1F
Amino sugar and nucleotide sugar metabolism	CHIT1, GALE, GNPDA1, NAGK, HEXB, AMDHD2, HEXA, UAP1L1, PMM2, NPL, NANS
mTOR signalling pathway	ATP6V1G1, SLC3A2, NRAS, RRAGC, RHEB, RRAGD, DVL2, ATP6V1H, MAPK1, LAMTOR1, ATP6V1E1, LAMTOR4, ATP6V1D, HRAS, FNIP2, LAMTOR3, ATP6V1C1, ATP6V1F, LAMTOR5
Circadian rhythm	PER3, BHLHE40, BHLHE41, CRY1, PRKAG2, RORA, CSNK1D, NR1D1

Palmieri *et al*. 2011, Sardiello *et al*. 2009, Carey *et al*. 2020

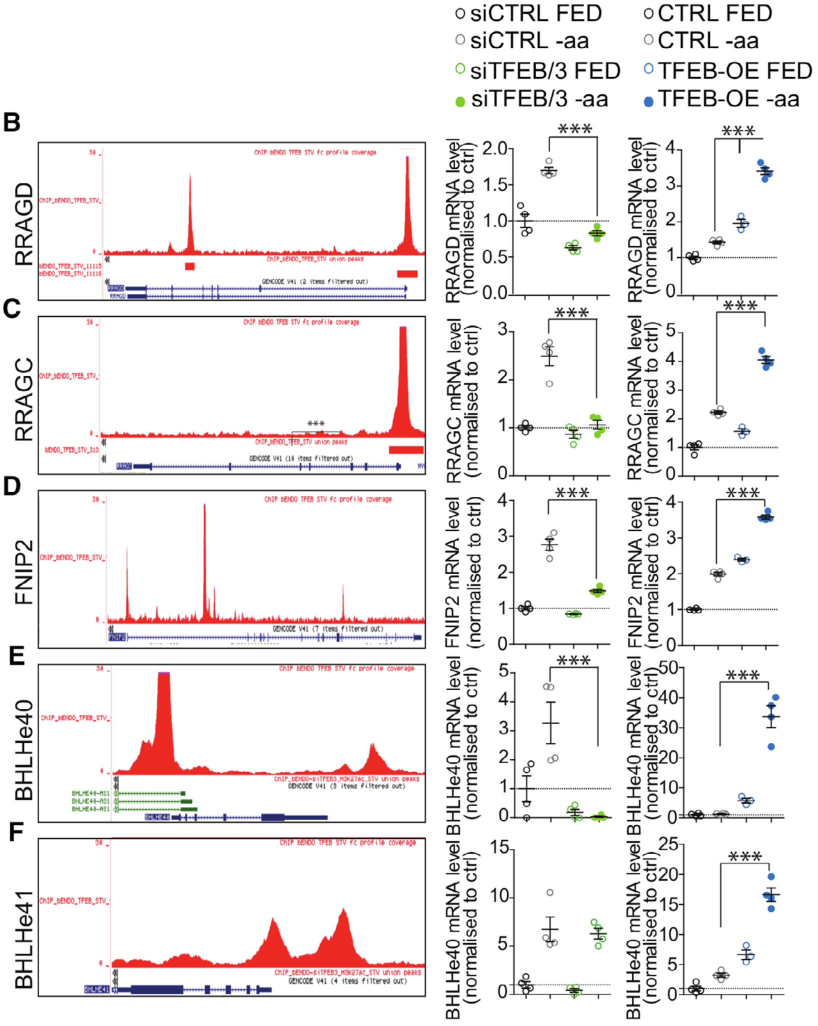

Figure EV2. ChIP-Seq analysis identifies direct TFEB targets in pancreatic beta cells.

A Top genes whose promoters are bound by TFEB upon fasting and upregulated upon TFEB upregulation in EndoC-BH1 cells sorted by gene categories. Genes in red are previously identified TFEB direct targets.

B–F On the left, alignment of the TFEB ChIP-seq track at the indicated gene loci. On the right, mRNA levels of the indicated genes from EndoC-βH1 cells treated with scramble siRNA (siCTRL) or TFEB- and TFE3-targeting siRNA (siTFEB/3) or TFEB-OE compared to control EndoC-βH1 cells incubated with full medium (FED) or upon amino acid starva tion (−aa) for 16 h. Data are represented as mean ± standard error. Each dot represents an independent experiment, ***$P < 0.001$; Student's two-tailed unpaired *t*-test.

 © 2023 The Authors

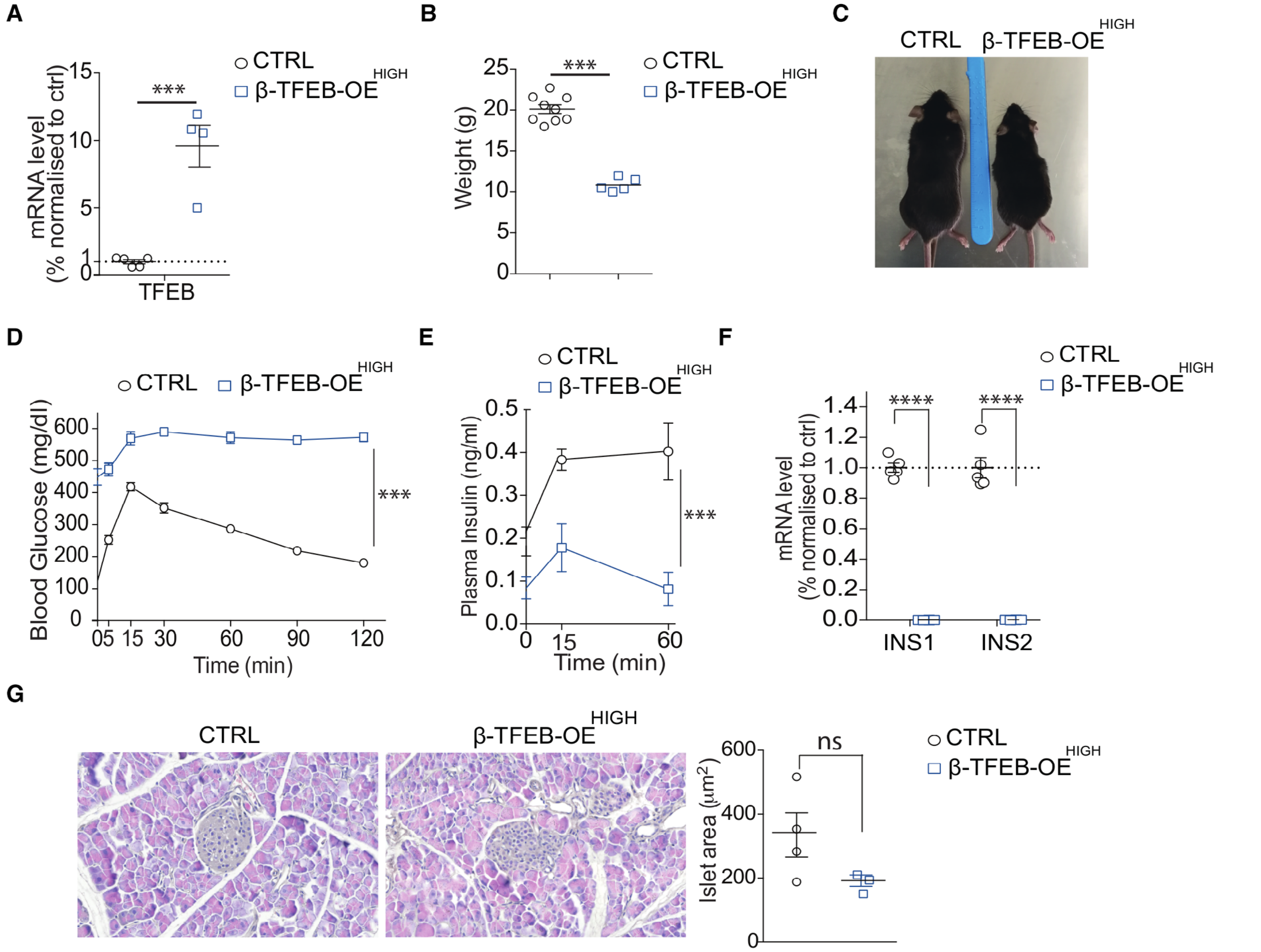

Figure EV3. TFEB upregulation in pancreatic β-cells in vivo results in suppression of insulin transcription and glucose intolerance.

A mRNA levels of TFEB in isolated islets from control and $\beta TFEBOE^{HIGH}$ mice. Each dot represents a mouse (n = 4-5/group). Data are represented as mean ± standard error. Student's two-tailed t-test: ***P-value < 0.001.

B Weight of control and $\beta TFEBOE^{HIGH}$ mice. Each dot represents a mouse (n = 9 for CTRL and n = 5 for $\beta TFEBOE^{HIGH}$ mice). Data are represented as mean ± standard error. Student's two-tailed t-test: ***P-value < 0.001.

C Representative images of control and $\beta TFEBOE^{HIGH}$ mice showing different body sizes.

D Glucose tolerance test (GTT) of control (n = 12) and $\beta TFEBOE^{HIGH}$ (n = 7) mice. Data are represented as mean ± standard error. Two-way ANOVA: ***P-value < 0.001.

E Glucose-stimulated insulin secretion (GSIS) for control (n = 11) and $\beta TFEBOE^{HIGH}$ (n = 7) mice. Data are represented as mean ± standard error. Two-way ANOVA: ***P-value < 0.001.

F mRNA levels of Ins1 and Ins2 in isolated islets from control and $\beta TFEBOE^{HIGH}$ mice. Each dot represents a mouse (n = 5/group). Data are represented as mean ± standard error. Student's two-tailed t-test: ****P-value < 0.0001.

G Representative images of pancreas slides from control and $\beta TFEBOE^{HIGH}$ mice, stained with hematoxylin/eosin and relative quantification of islet area (n = 3-4/ group). Data are represented as mean ± standard error.

© 2023 The Authors
