# Supplementary figures and images for: TFEB and TFE3 control glucose homeostasis by regulating insulin gene expression
Source: EMBO J. 2023 Sep 15;42(21):e113928. doi: 10.15252/embj.2023113928 (PMC10620765; doi:10.15252/embj.2023113928)

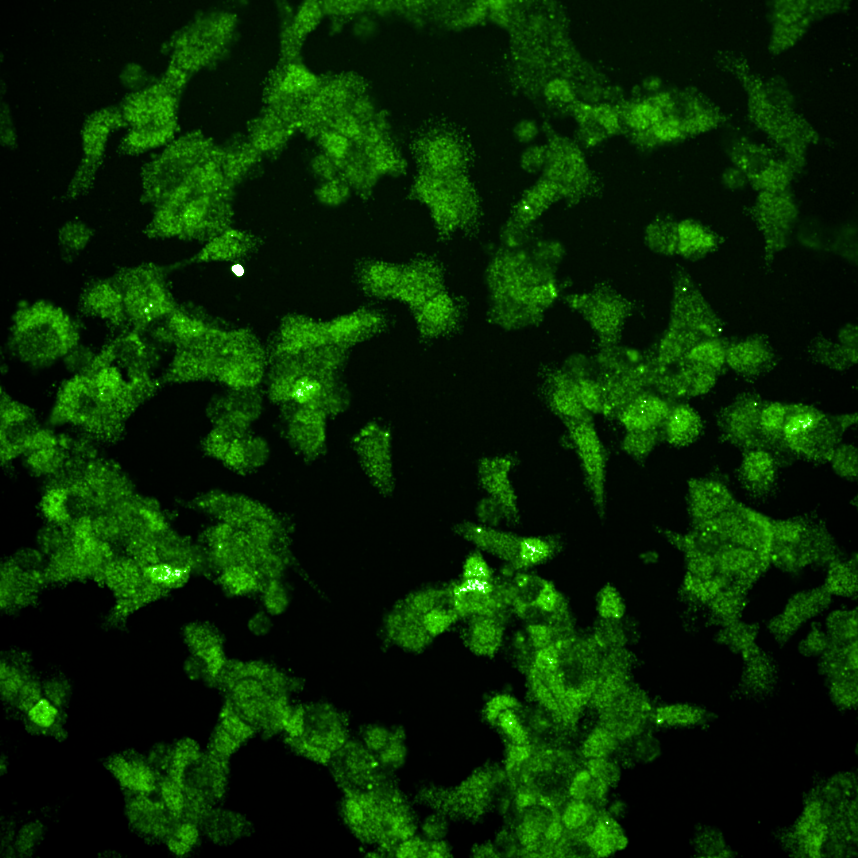

Supplement: Supplementary file 15 — Source Data for Figure 1 [file EMBJ-42-e113928-s013.zip › Figure 1/1A/no glc no aa.bmp]

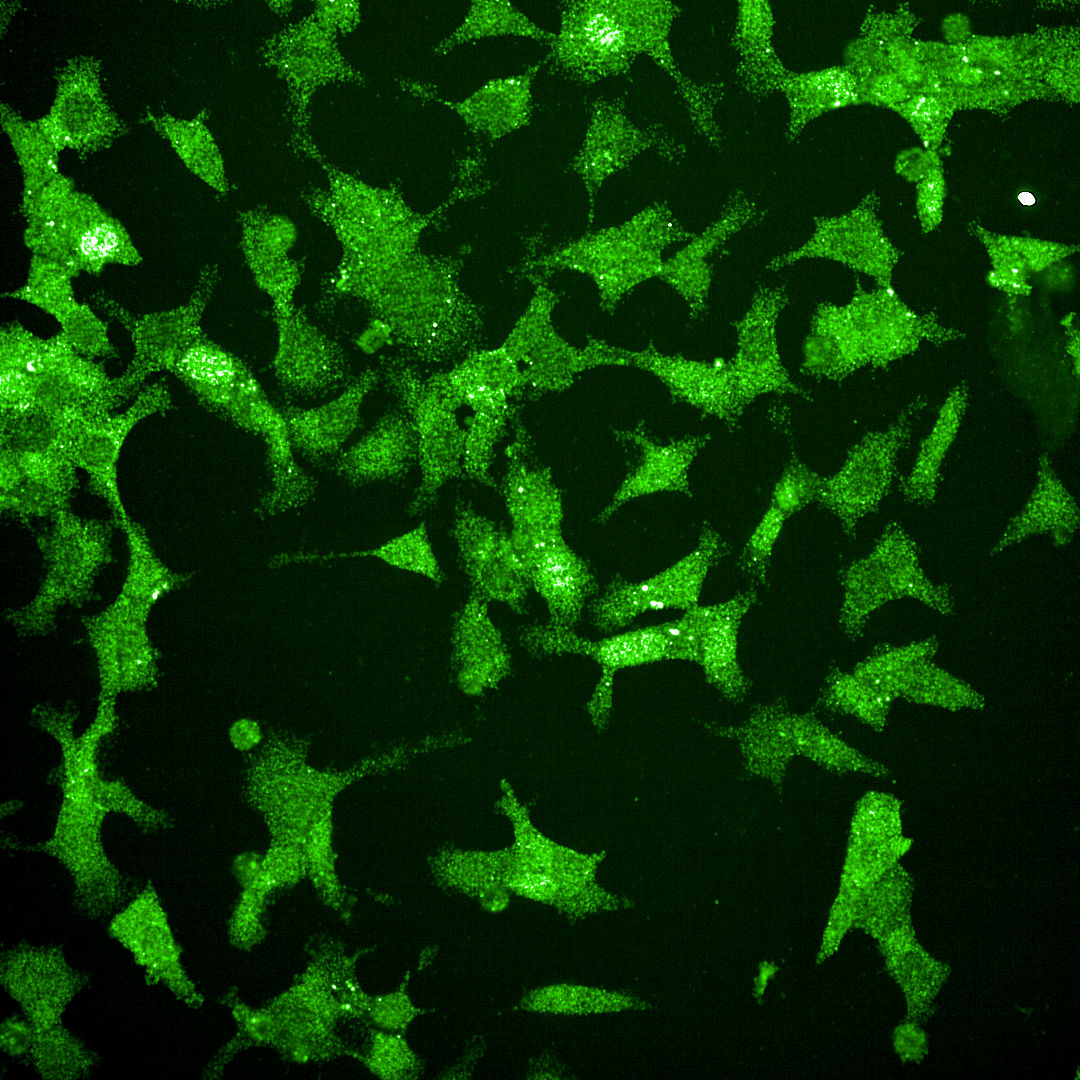

Supplement: Supplementary file 15 — Source Data for Figure 1 [file EMBJ-42-e113928-s013.zip › Figure 1/1A/glc2.png]

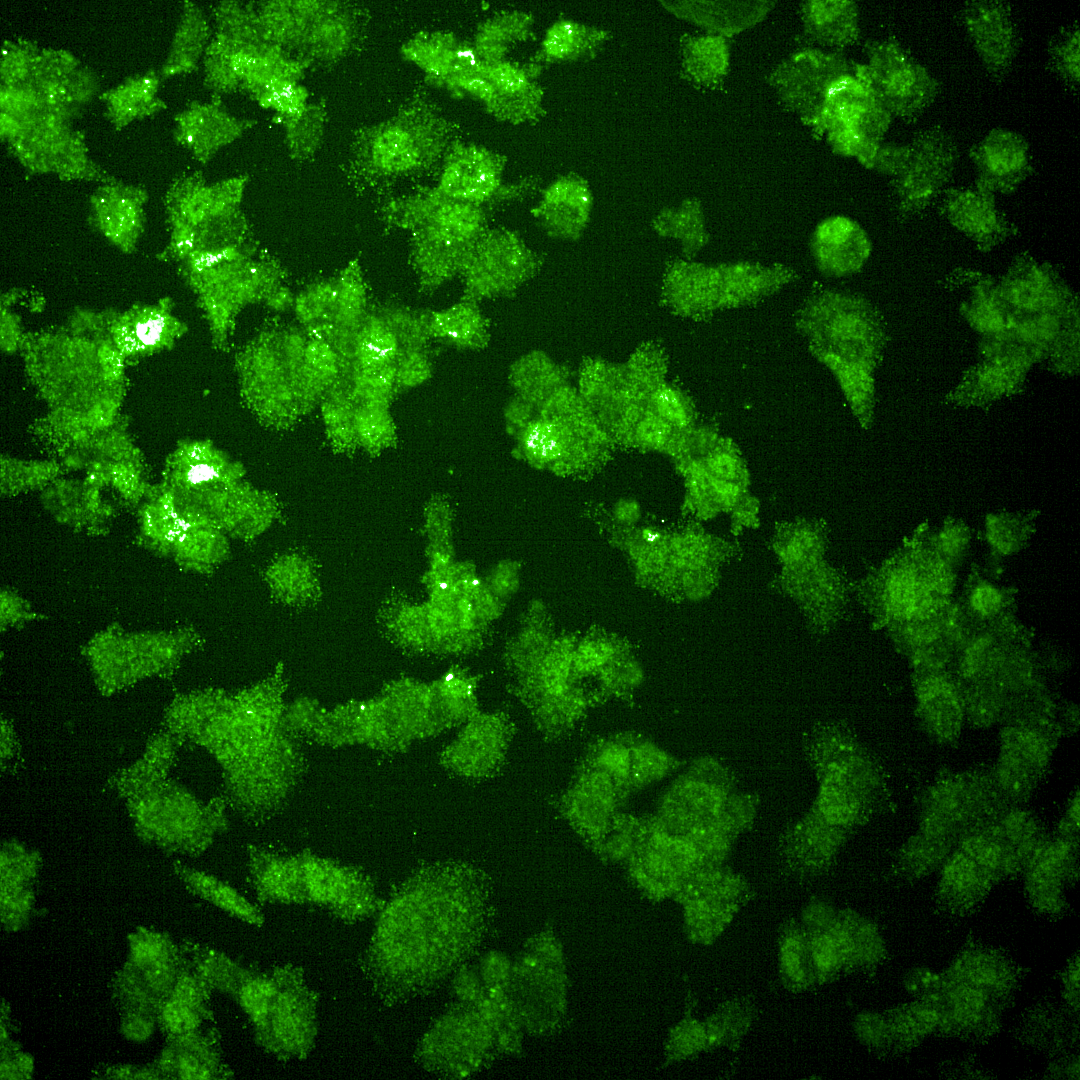

Supplement: Supplementary file 15 — Source Data for Figure 1 [file EMBJ-42-e113928-s013.zip › Figure 1/1A/fasting.png]

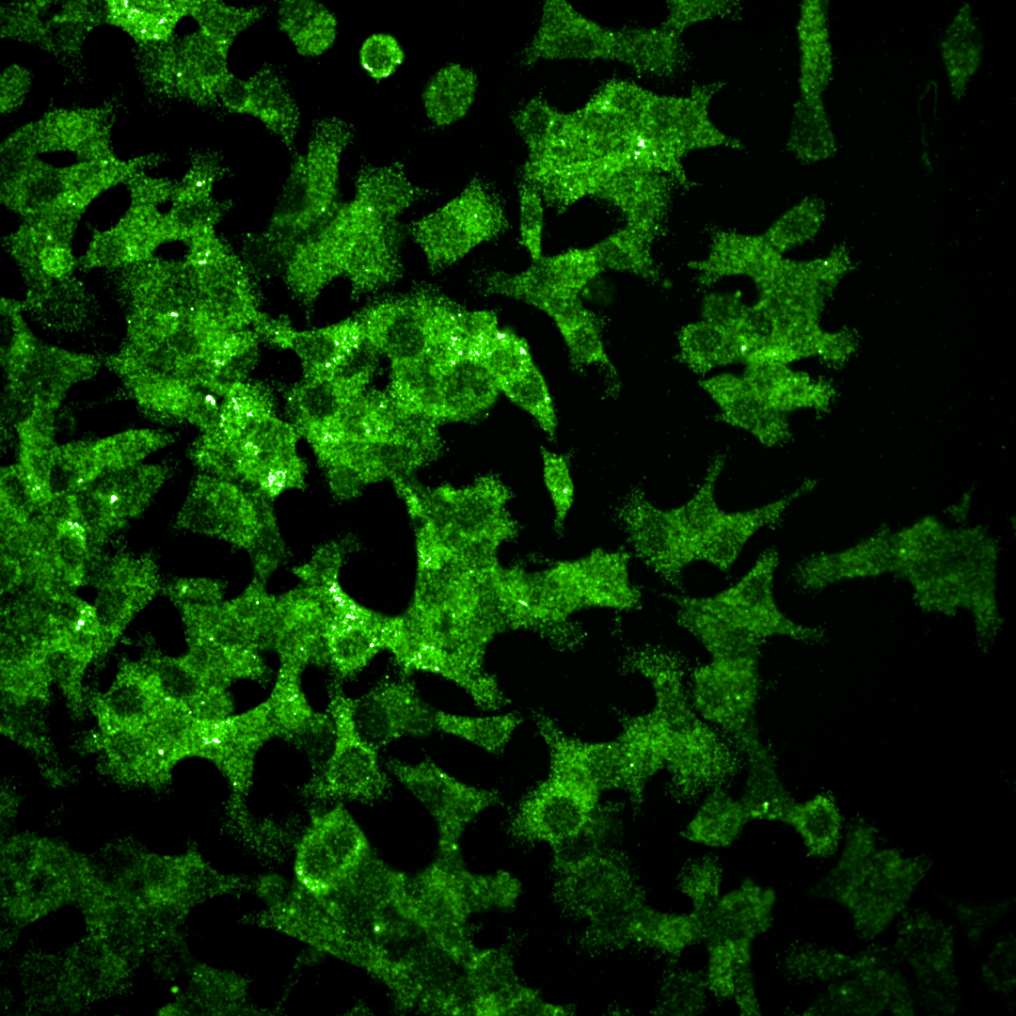

Supplement: Supplementary file 15 — Source Data for Figure 1 [file EMBJ-42-e113928-s013.zip › Figure 1/1A/acc fasting refeeding.bmp]

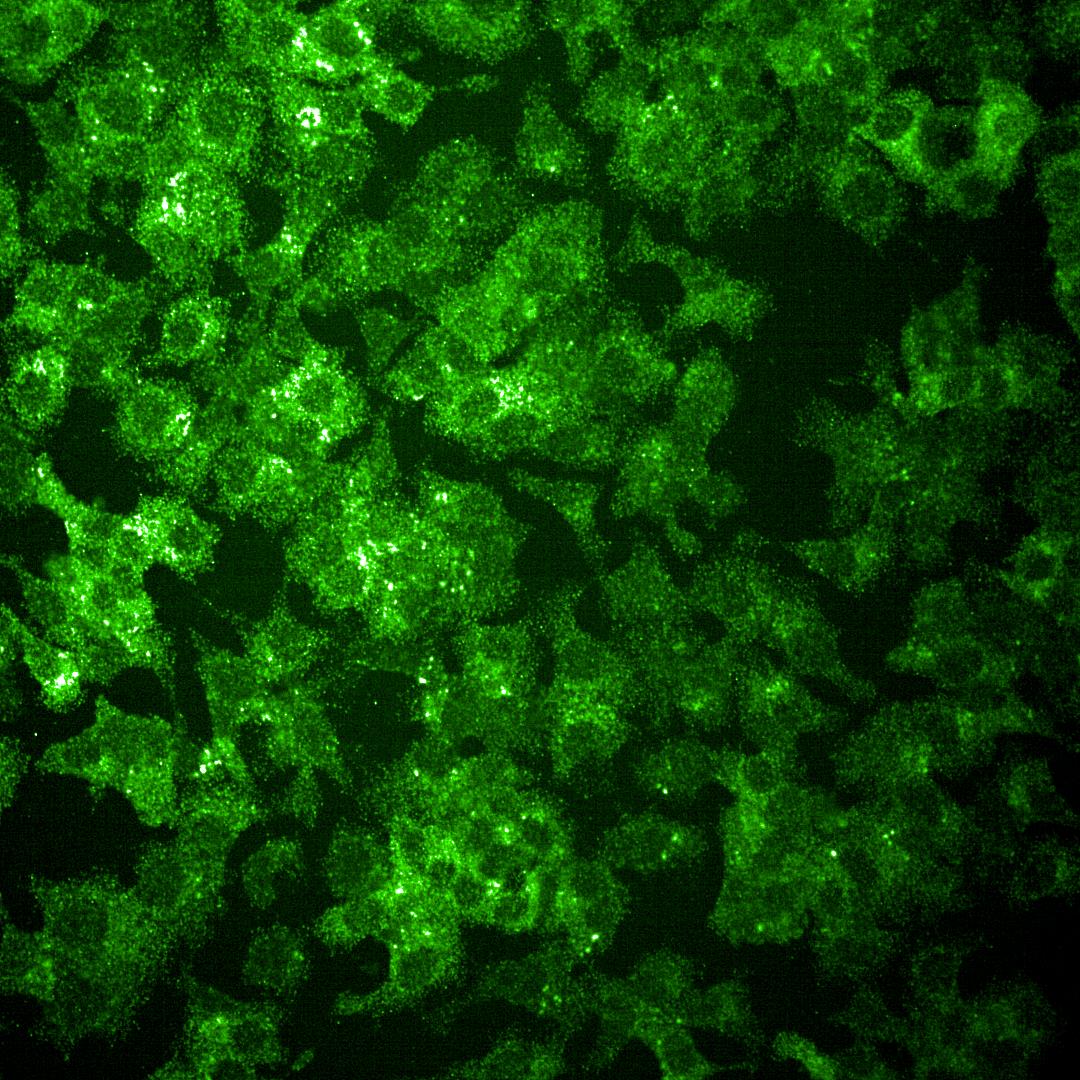

Supplement: Supplementary file 15 — Source Data for Figure 1 [file EMBJ-42-e113928-s013.zip › Figure 1/1A/NT.jpg]

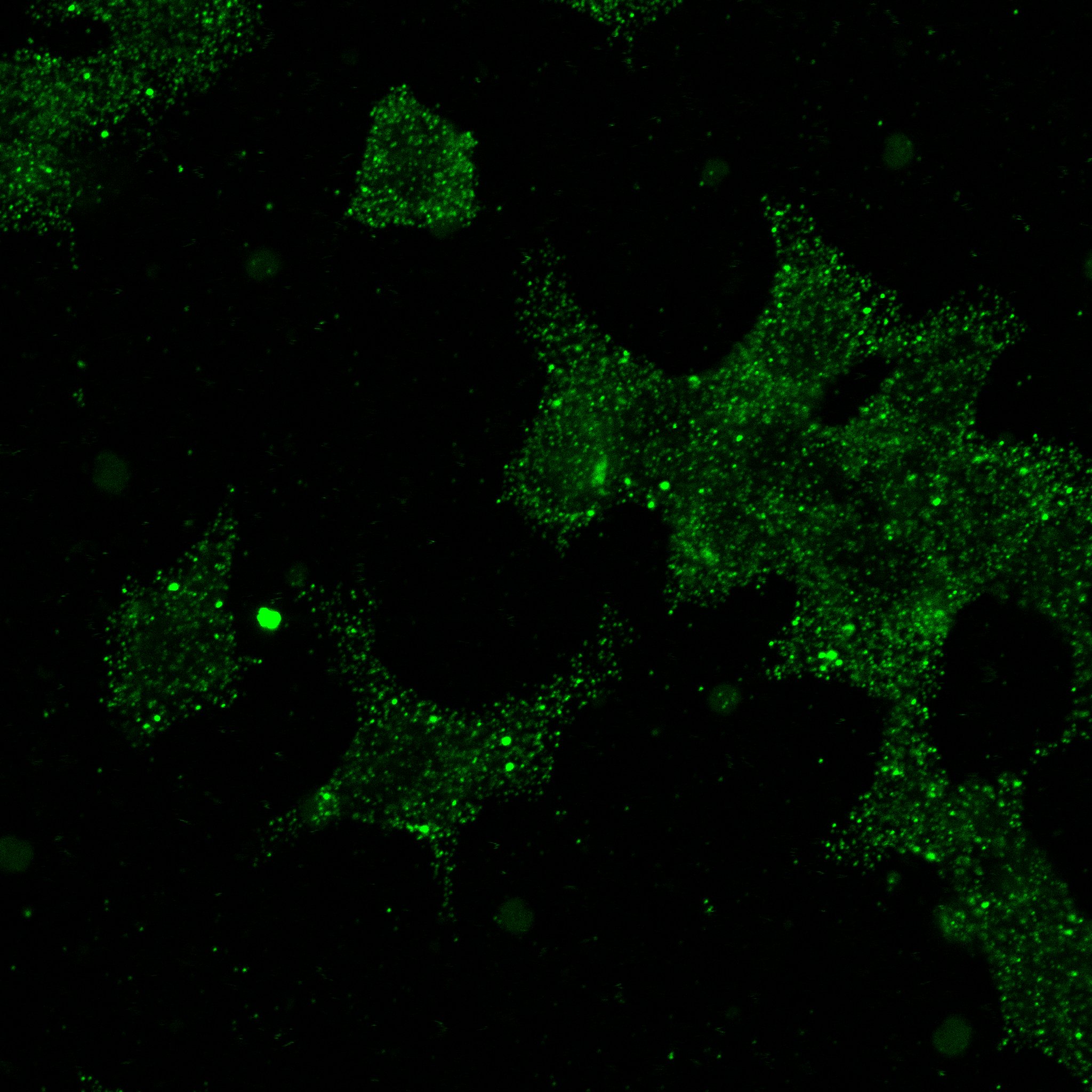

Supplement: Supplementary file 15 — Source Data for Figure 1 [file EMBJ-42-e113928-s013.zip › Figure 1/1D/FED TFEB.jpg]

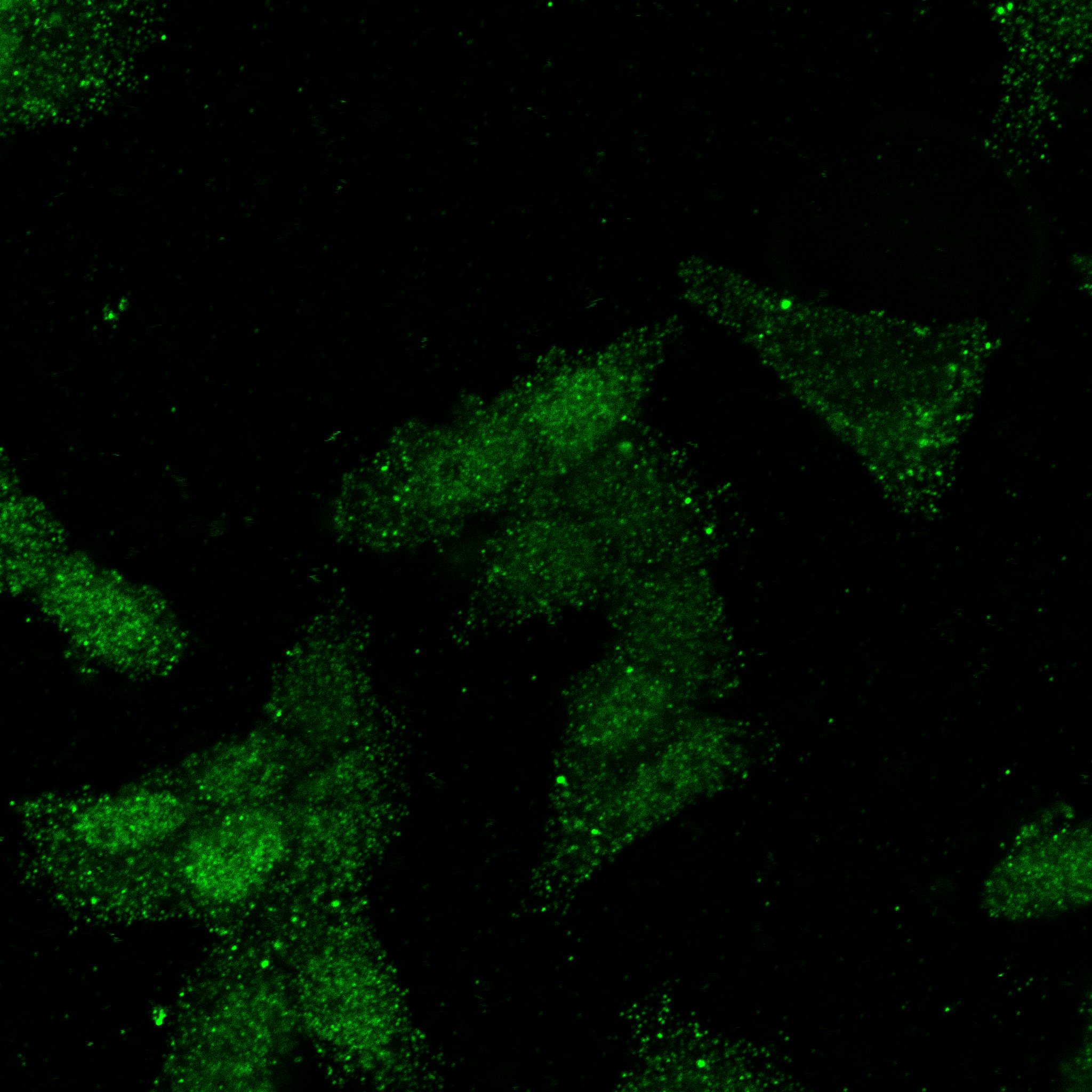

Supplement: Supplementary file 15 — Source Data for Figure 1 [file EMBJ-42-e113928-s013.zip › Figure 1/1D/GLU STARV TFEB.jpg]

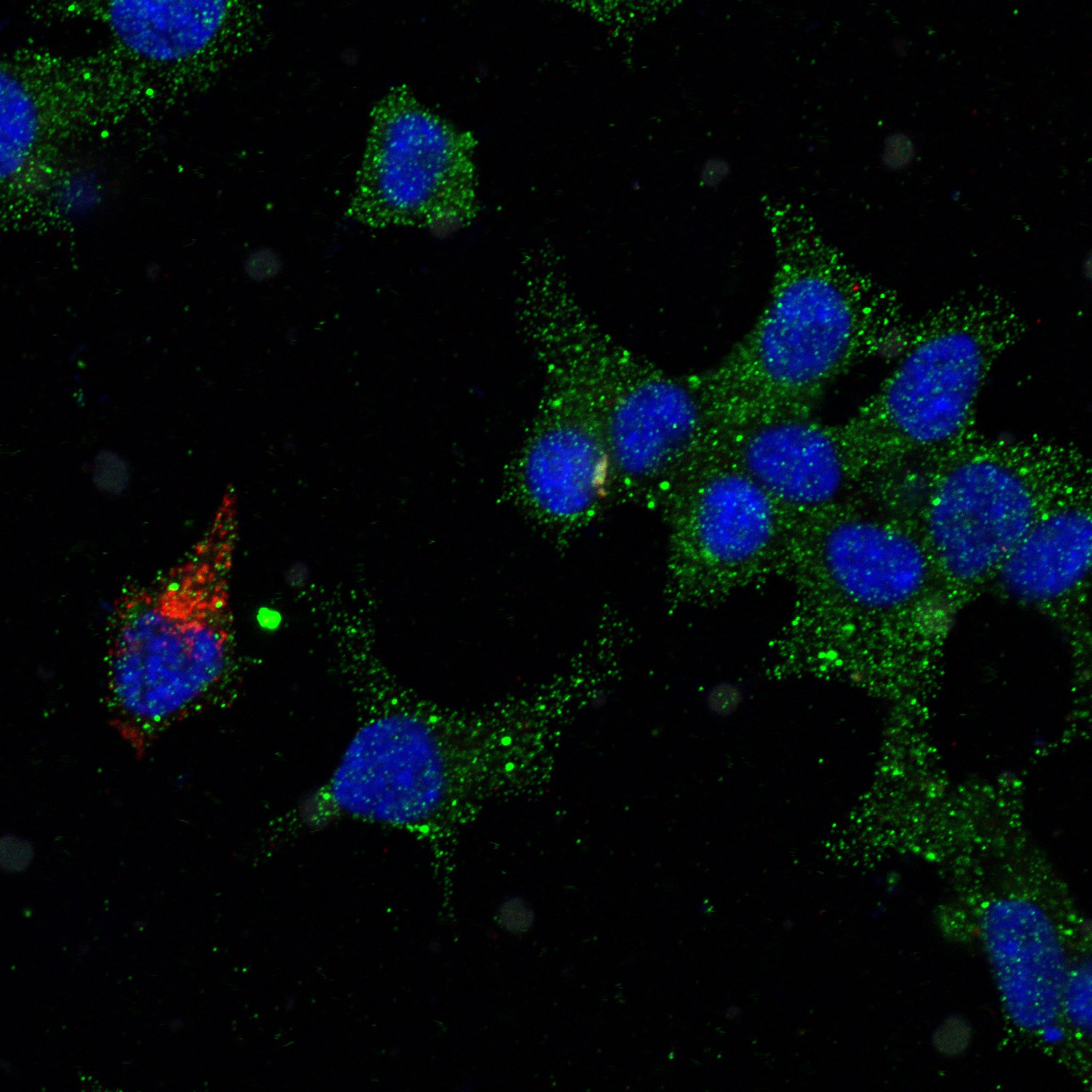

Supplement: Supplementary file 15 — Source Data for Figure 1 [file EMBJ-42-e113928-s013.zip › Figure 1/1D/FED MERGE TOT.jpg]

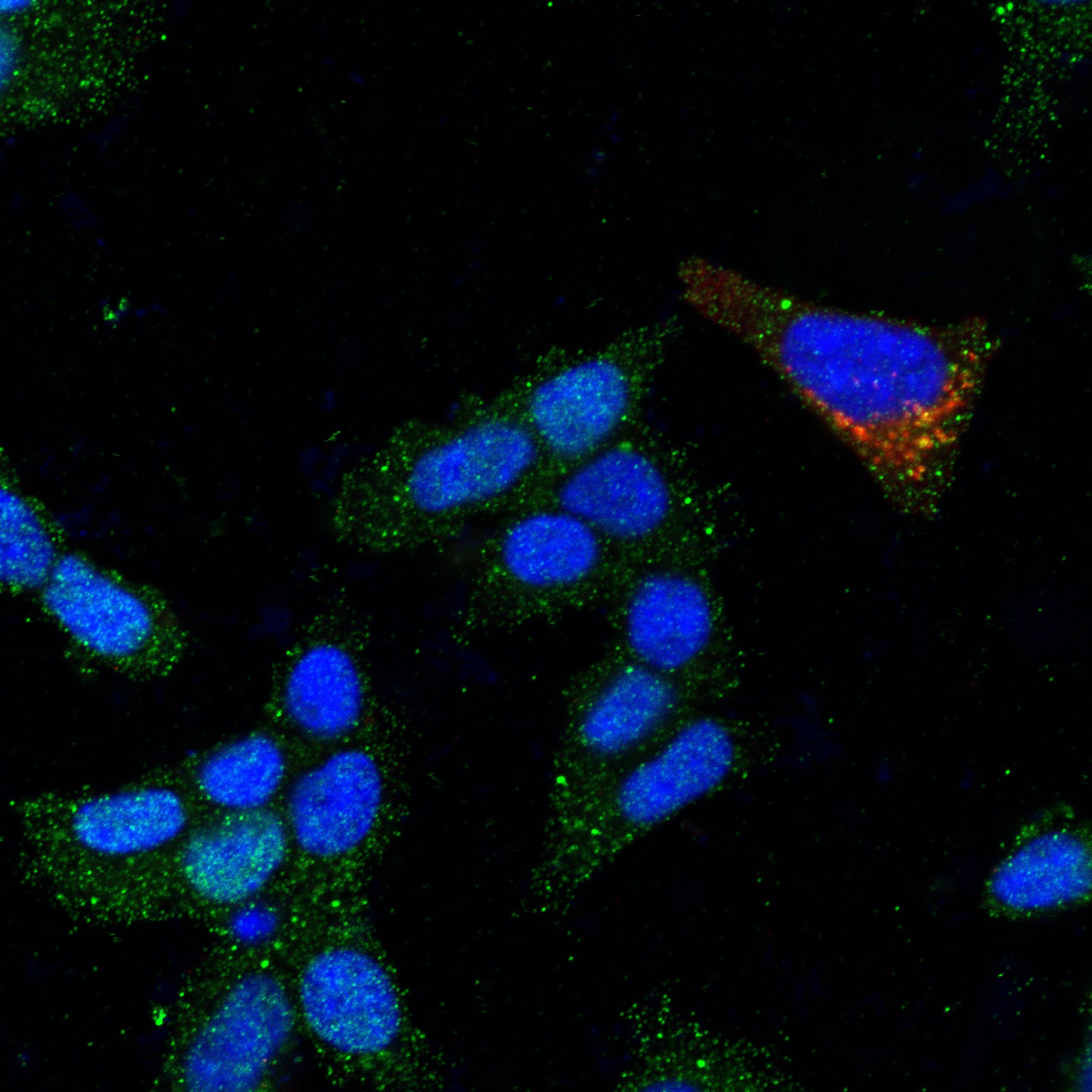

Supplement: Supplementary file 15 — Source Data for Figure 1 [file EMBJ-42-e113928-s013.zip › Figure 1/1D/GLU STARV MERGE TOT.jpg]

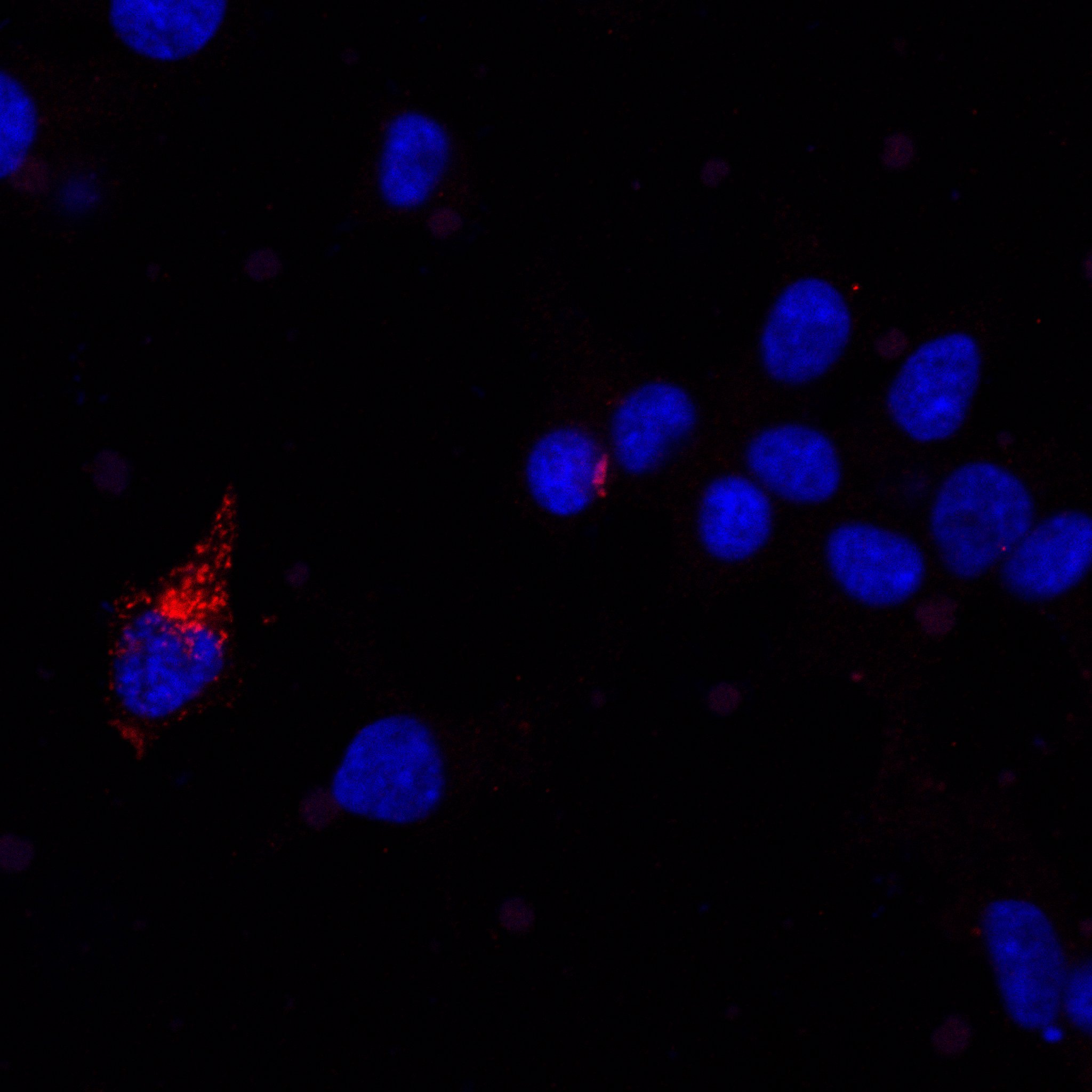

Supplement: Supplementary file 15 — Source Data for Figure 1 [file EMBJ-42-e113928-s013.zip › Figure 1/1D/FED HA DAPI.jpg]

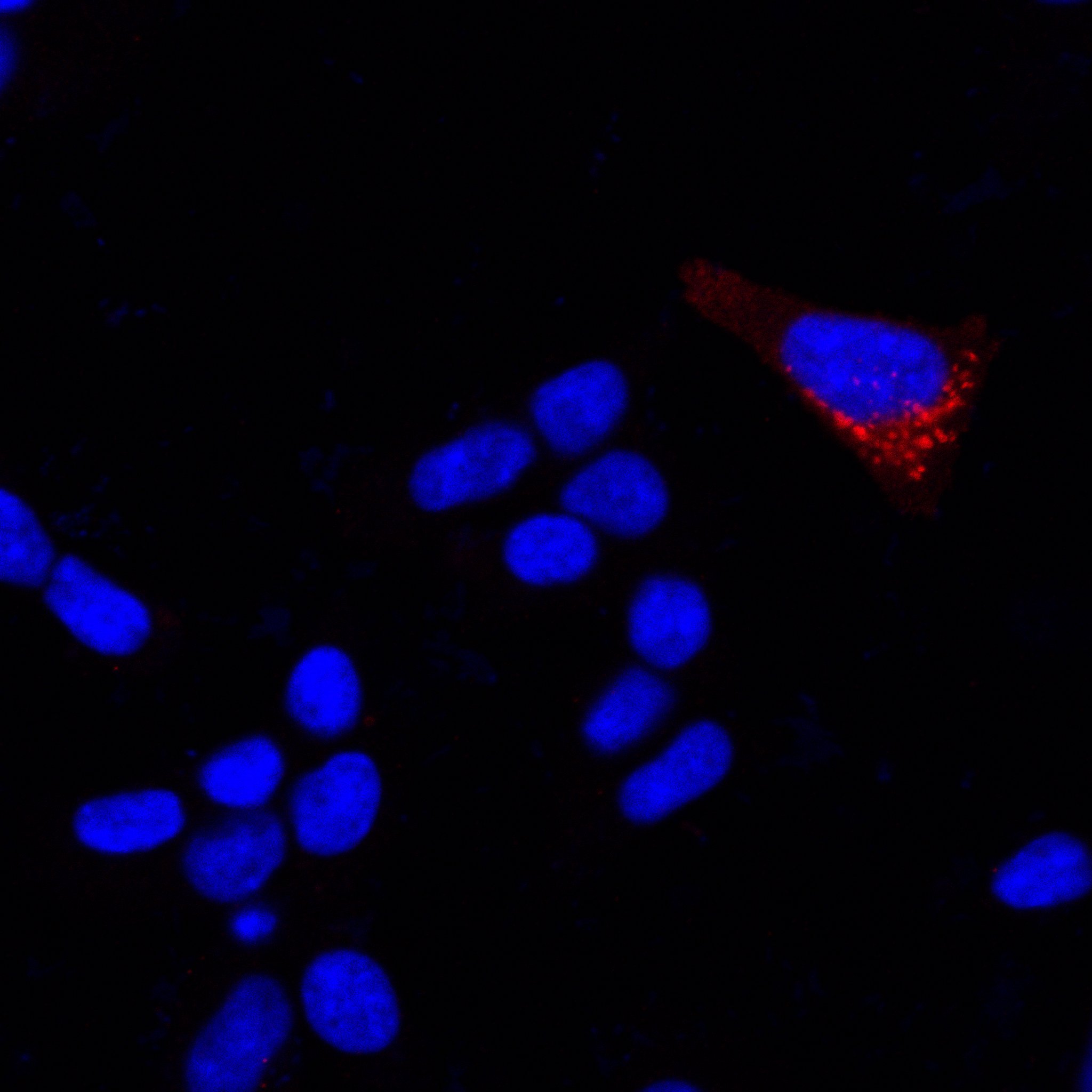

Supplement: Supplementary file 15 — Source Data for Figure 1 [file EMBJ-42-e113928-s013.zip › Figure 1/1D/GLU STARV HA DAPI.jpg]

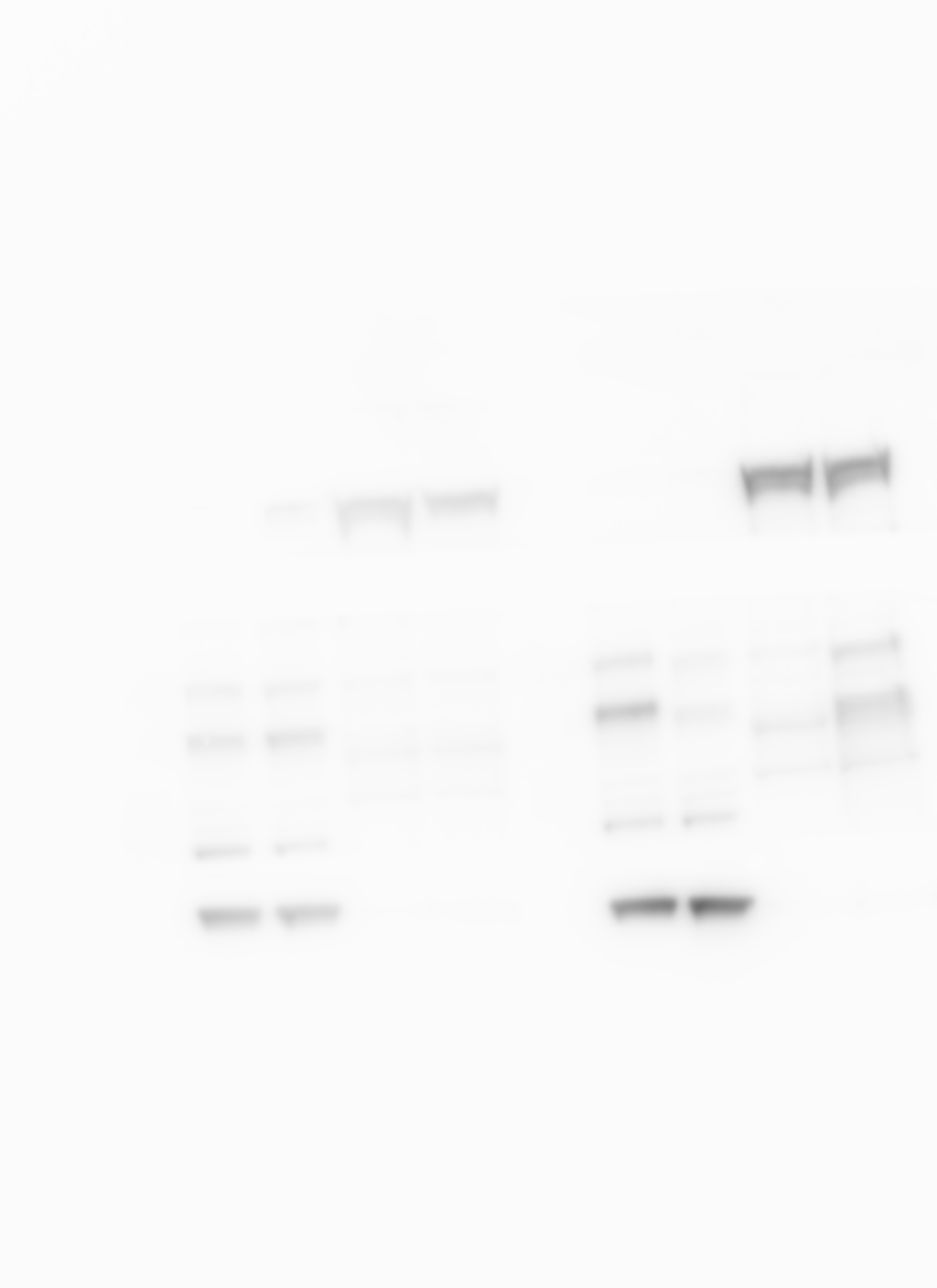

Supplement: Supplementary file 15 — Source Data for Figure 1 [file EMBJ-42-e113928-s013.zip › Figure 1/1C/PARP GAPDH.tif]

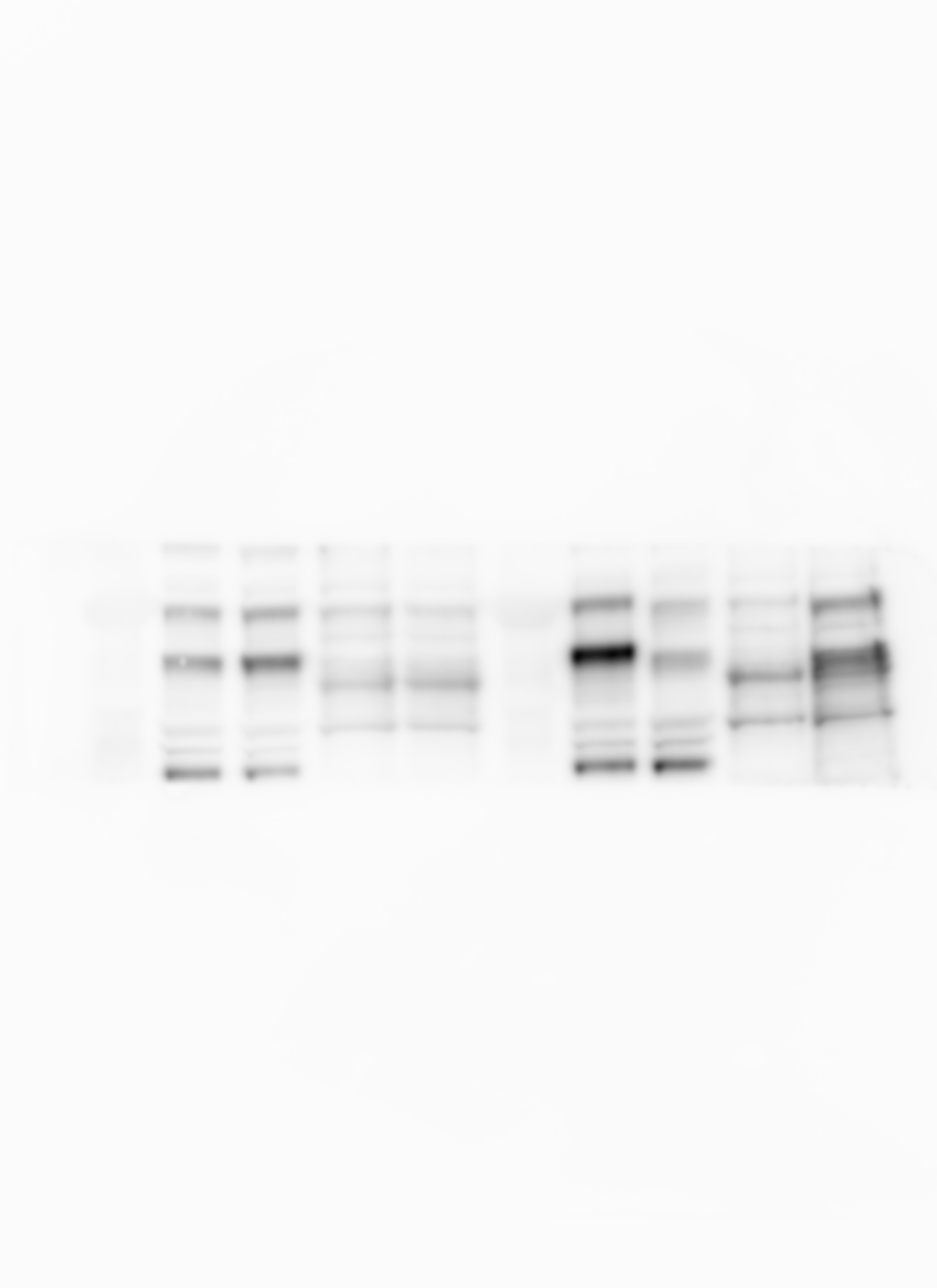

Supplement: Supplementary file 15 — Source Data for Figure 1 [file EMBJ-42-e113928-s013.zip › Figure 1/1C/TFE3.tif]

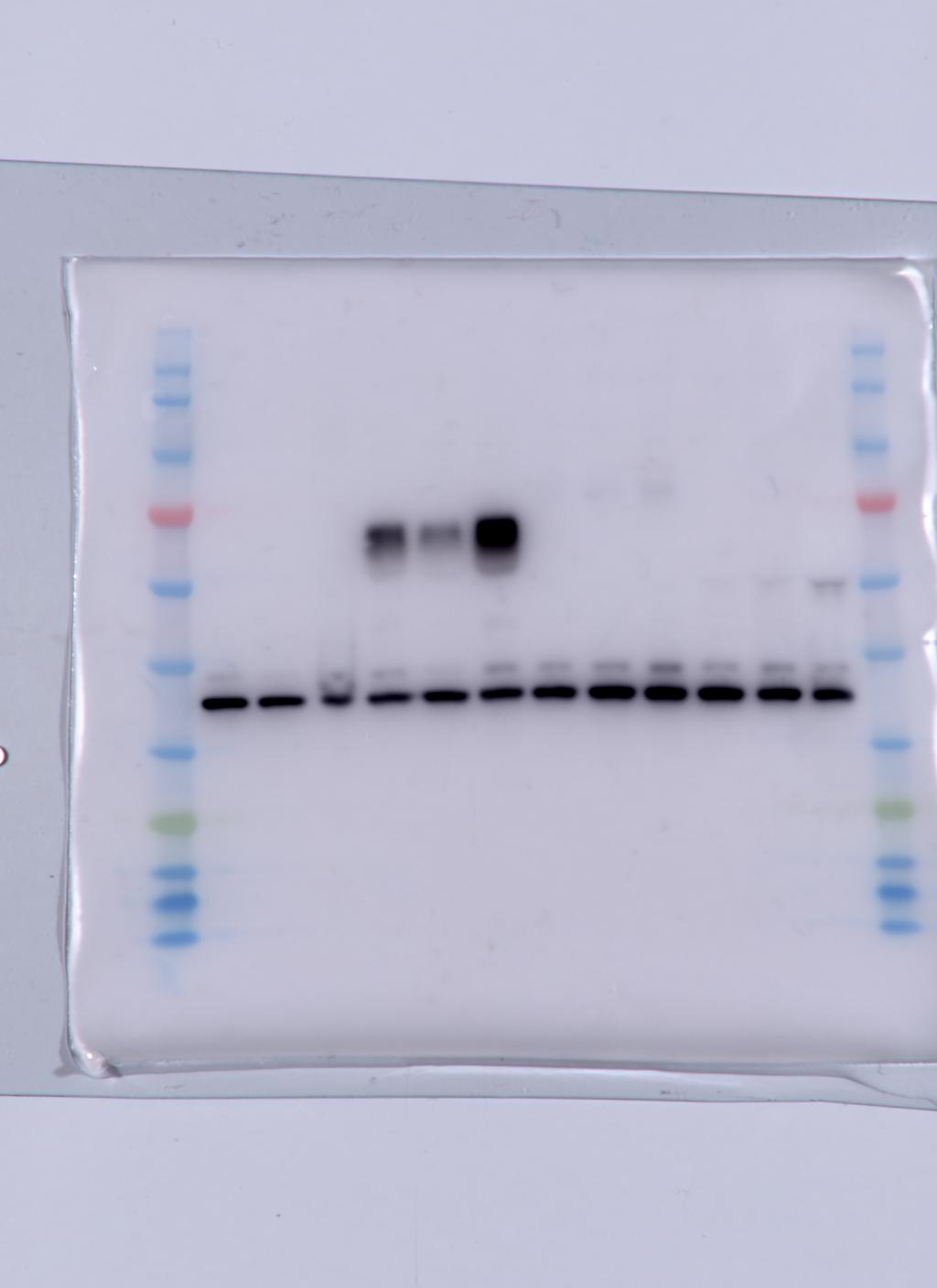

Supplement: Supplementary file 16 — Source Data for Figure 2 [file EMBJ-42-e113928-s007.zip › Figure 2/2A/WB TFEB GAPDH.jpg]

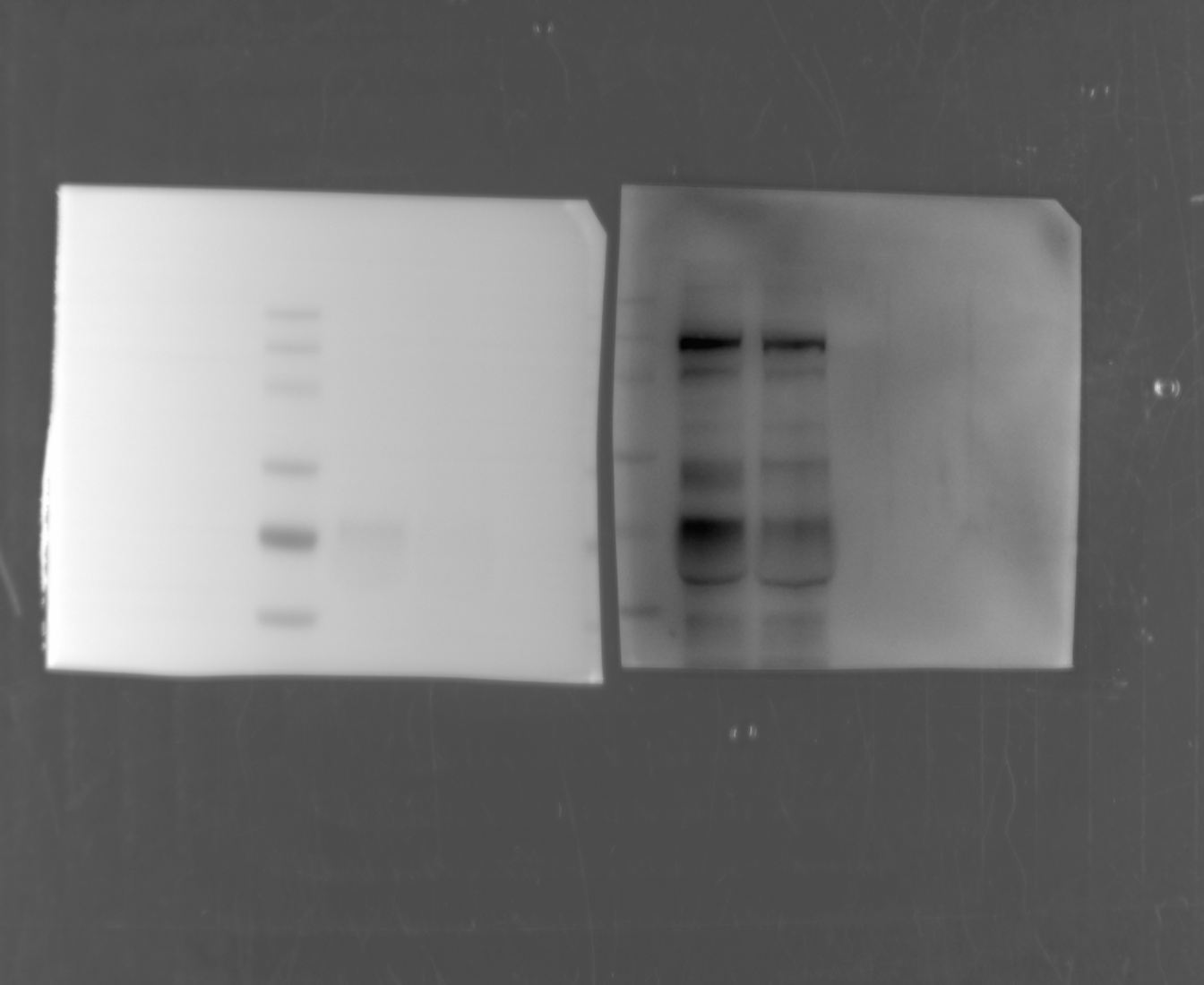

Supplement: Supplementary file 16 — Source Data for Figure 2 [file EMBJ-42-e113928-s007.zip › Figure 2/2F/SITFEB3 TFE3 invert.tif]

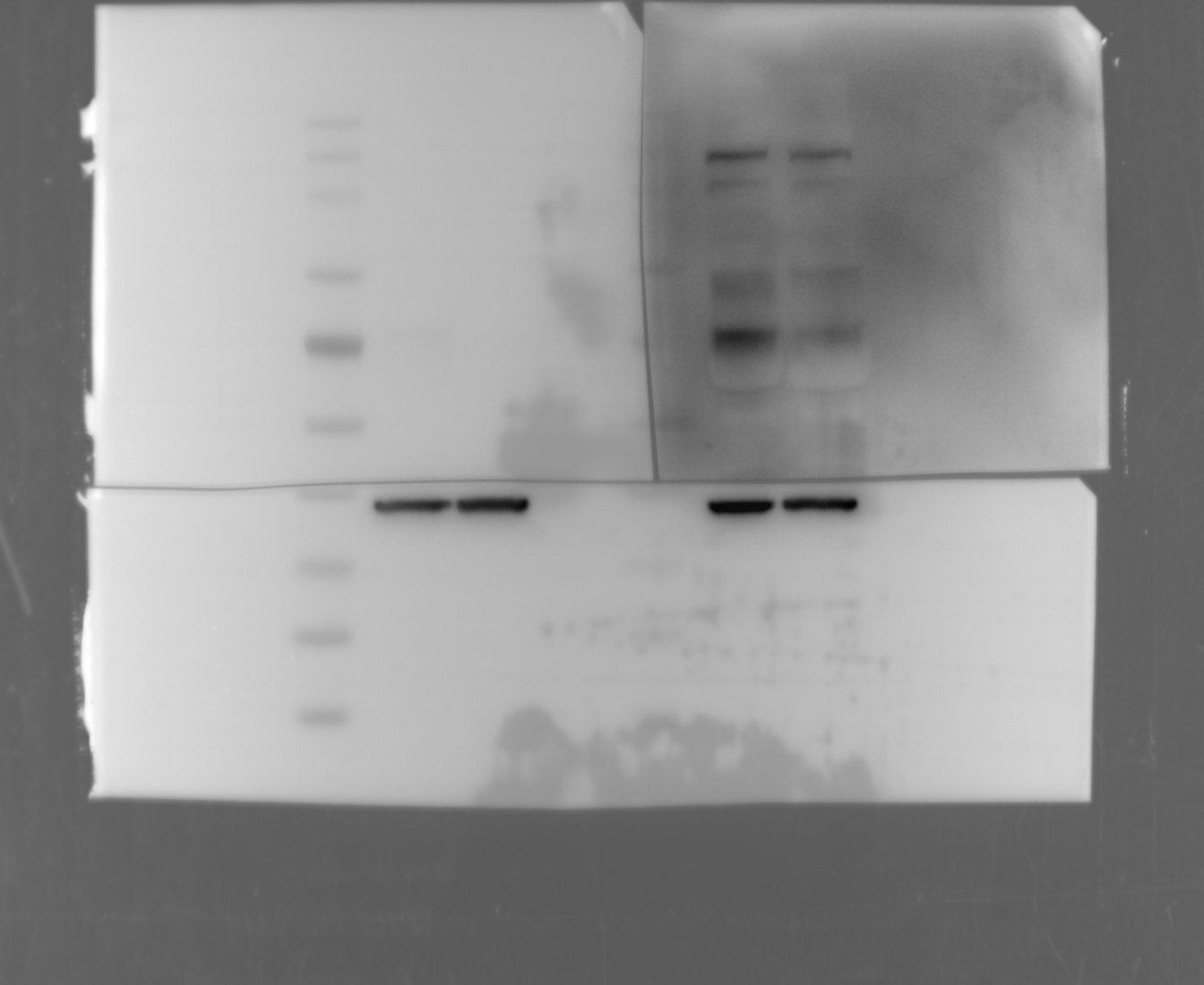

Supplement: Supplementary file 16 — Source Data for Figure 2 [file EMBJ-42-e113928-s007.zip › Figure 2/2F/SITFEB3 GAPDH inert.jpg]

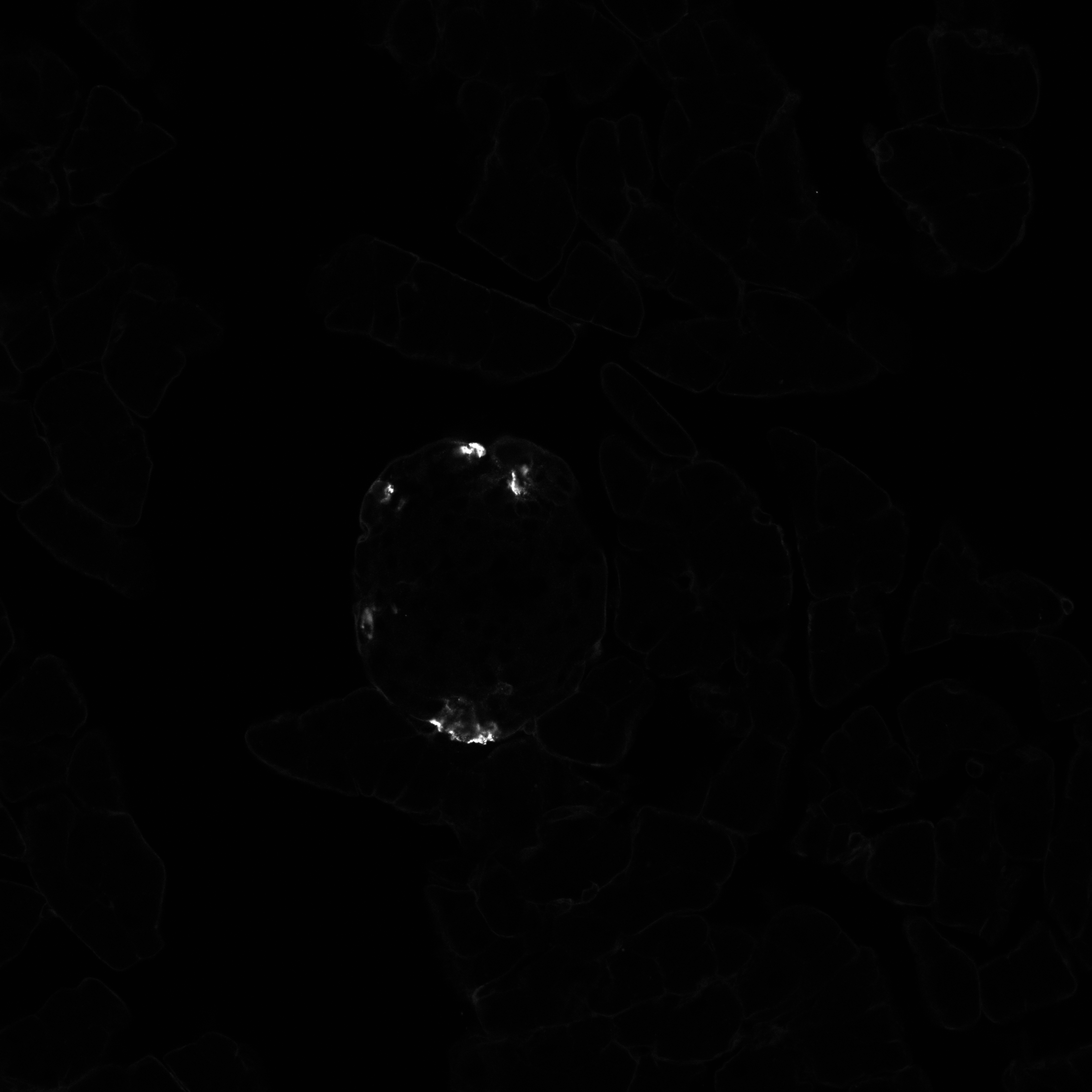

Supplement: Supplementary file 17 — Source Data for Figure 4 [file EMBJ-42-e113928-s006.zip › Figure 4/4H/CTRL GLUCAGON.tif]

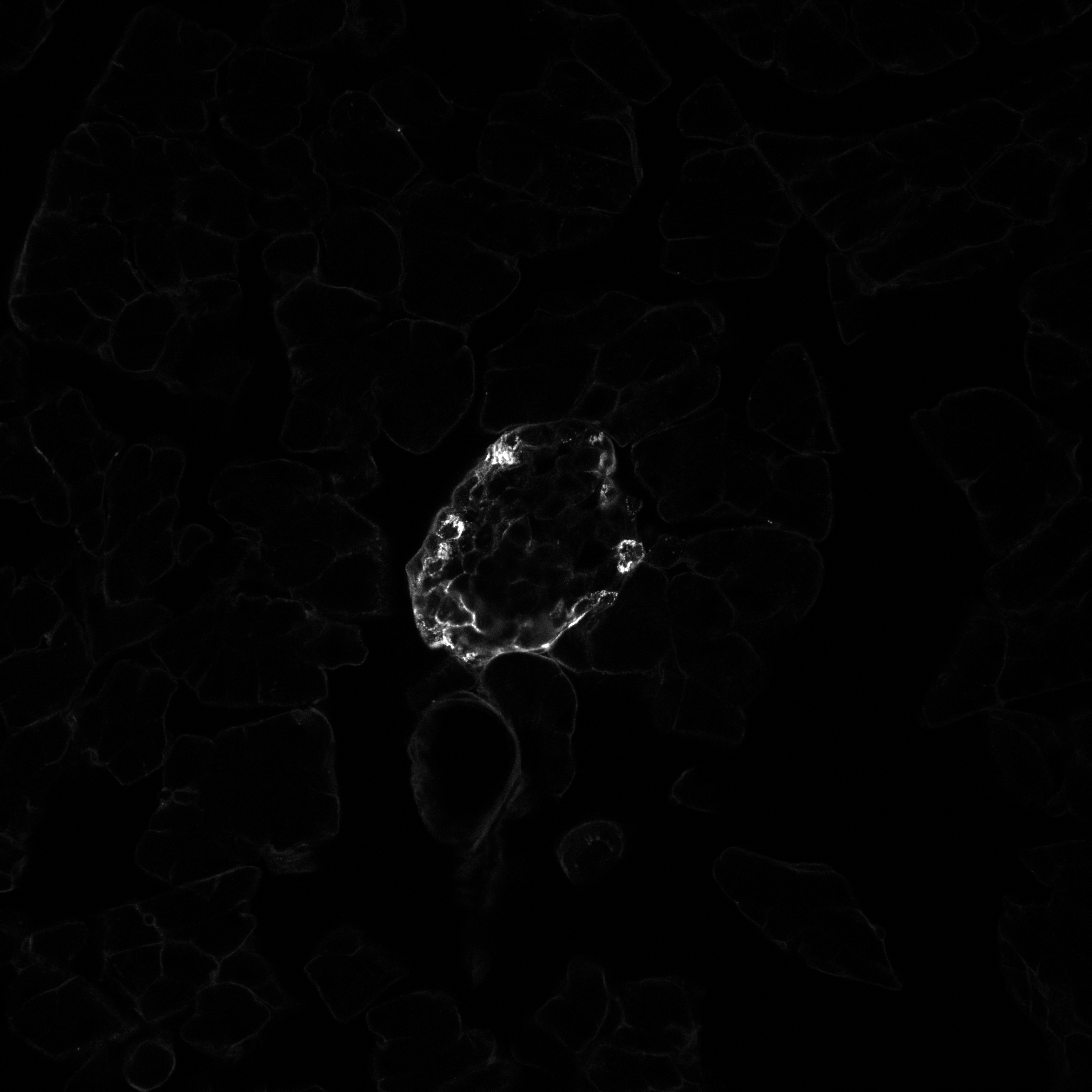

Supplement: Supplementary file 17 — Source Data for Figure 4 [file EMBJ-42-e113928-s006.zip › Figure 4/4H/TFEB OE GLUCAGON.tif]

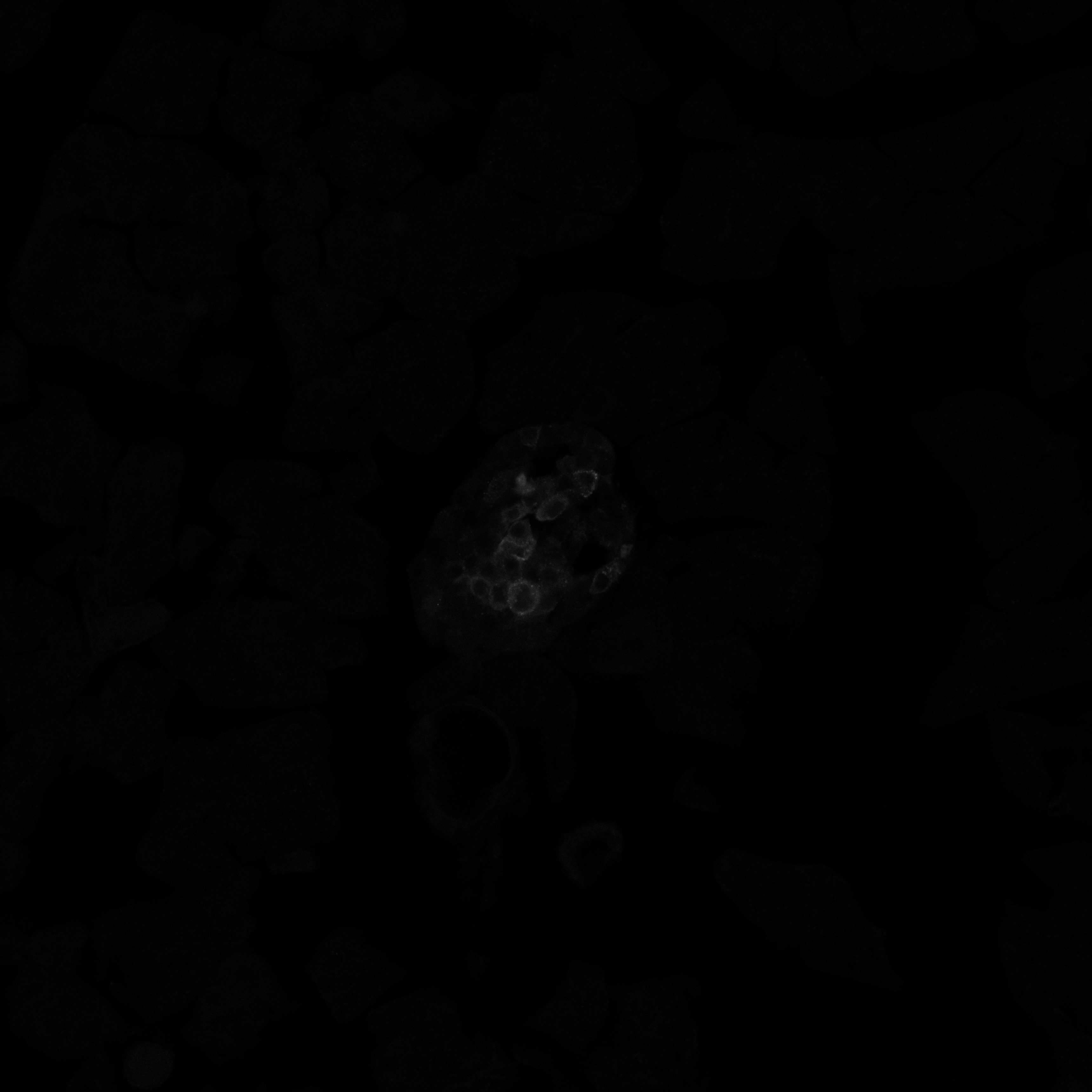

Supplement: Supplementary file 17 — Source Data for Figure 4 [file EMBJ-42-e113928-s006.zip › Figure 4/4H/TFEB OE INS.tif]

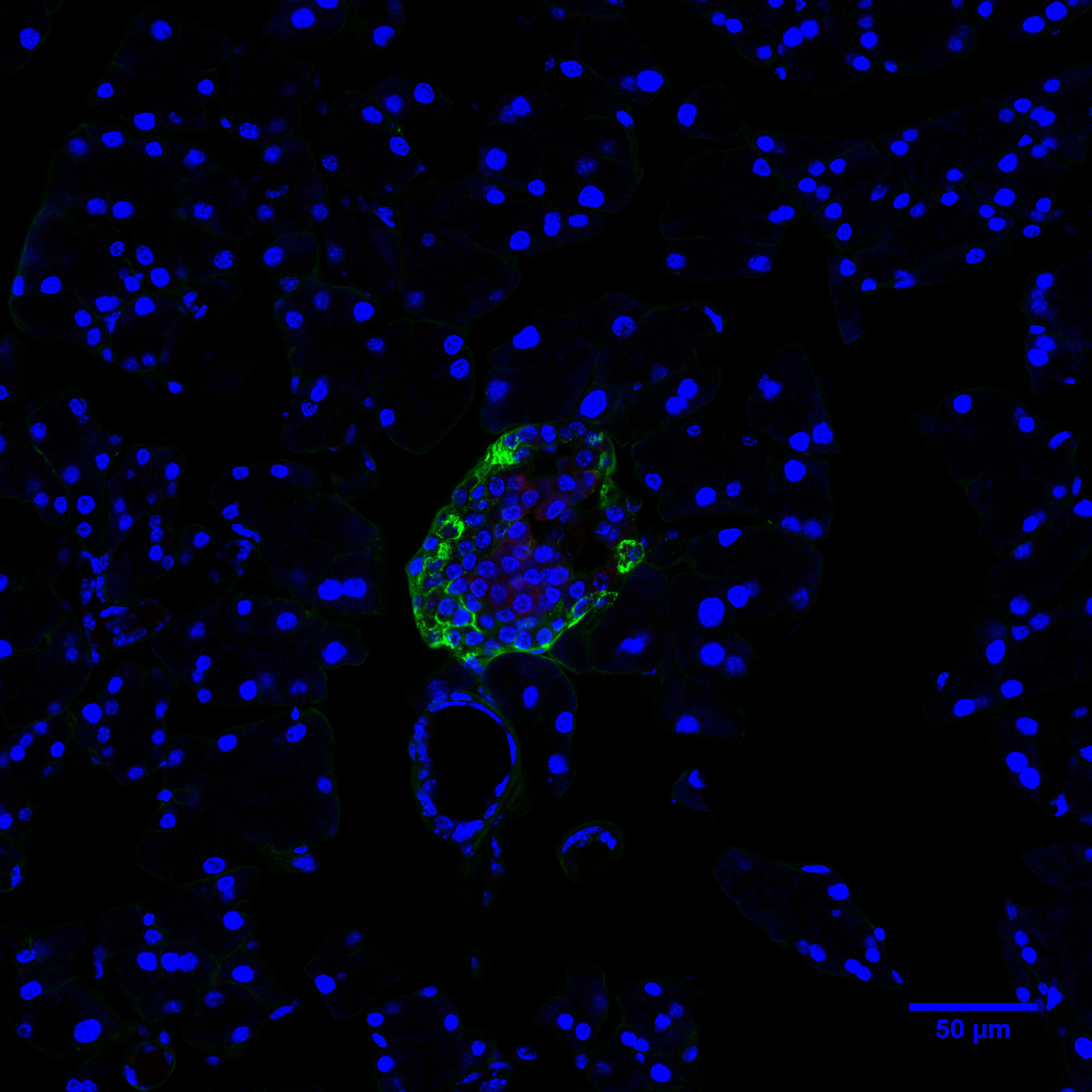

Supplement: Supplementary file 17 — Source Data for Figure 4 [file EMBJ-42-e113928-s006.zip › Figure 4/4H/TFEB OE MERGE.tif]

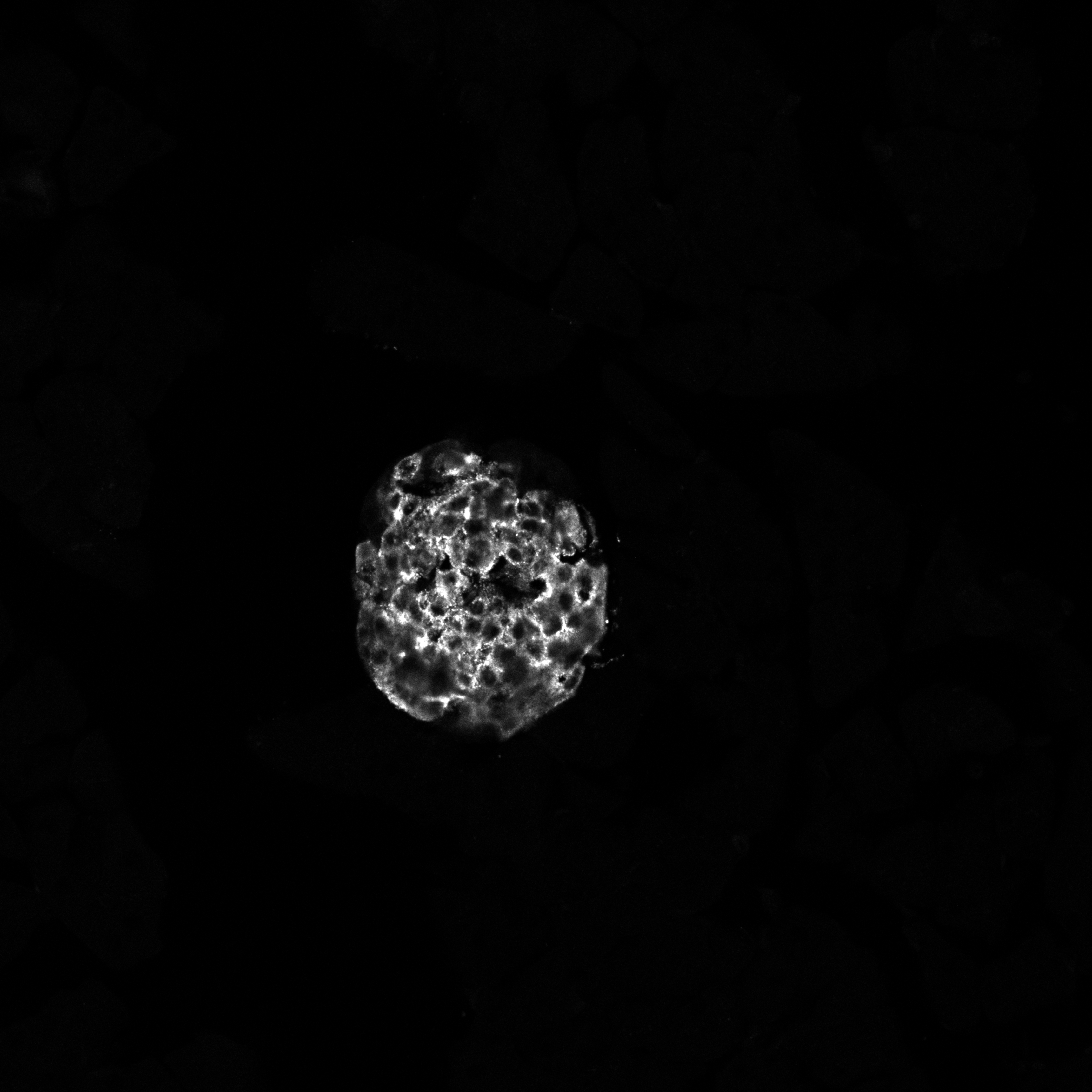

Supplement: Supplementary file 17 — Source Data for Figure 4 [file EMBJ-42-e113928-s006.zip › Figure 4/4H/CTRL INS.tif]

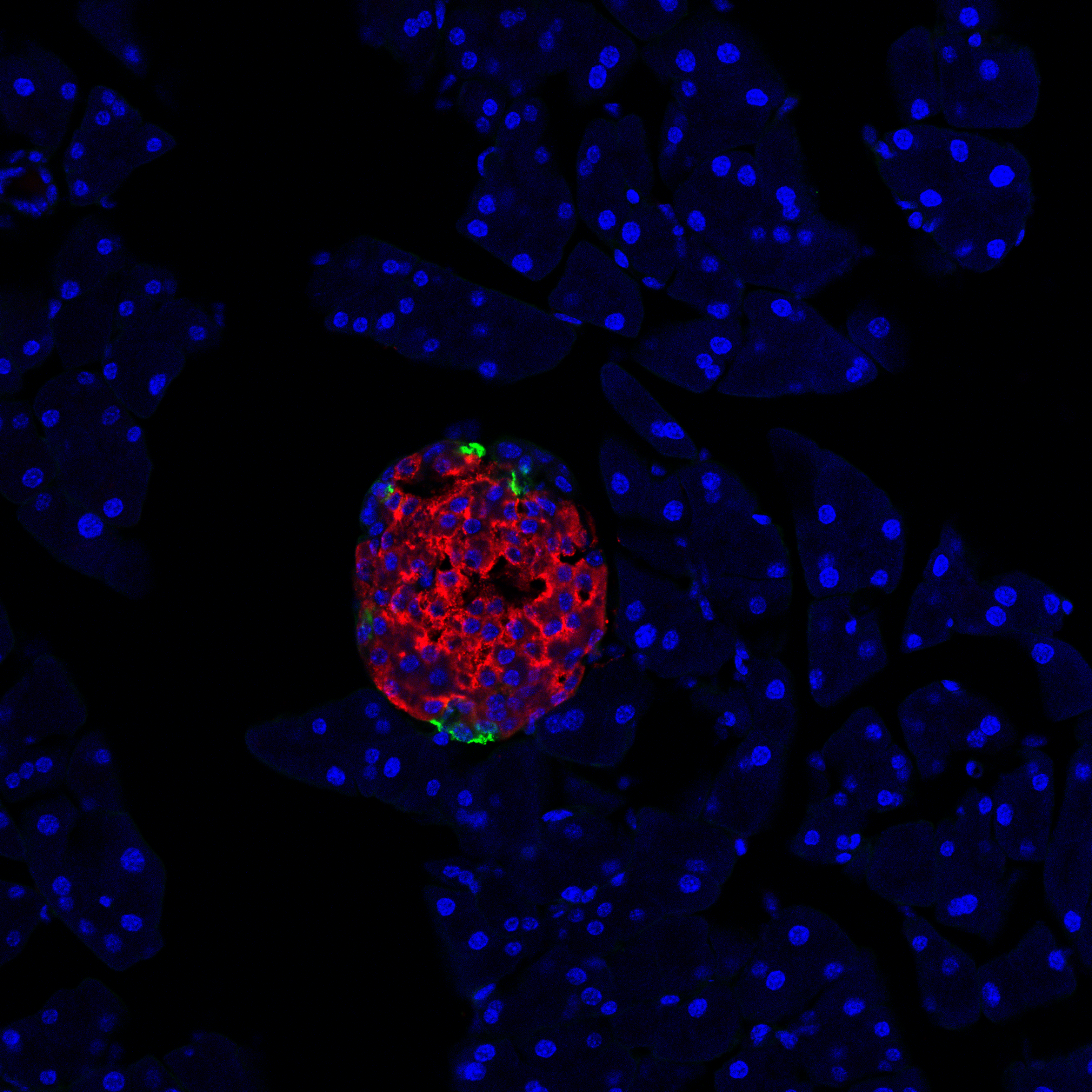

Supplement: Supplementary file 17 — Source Data for Figure 4 [file EMBJ-42-e113928-s006.zip › Figure 4/4H/CTRL MERGE.tif]

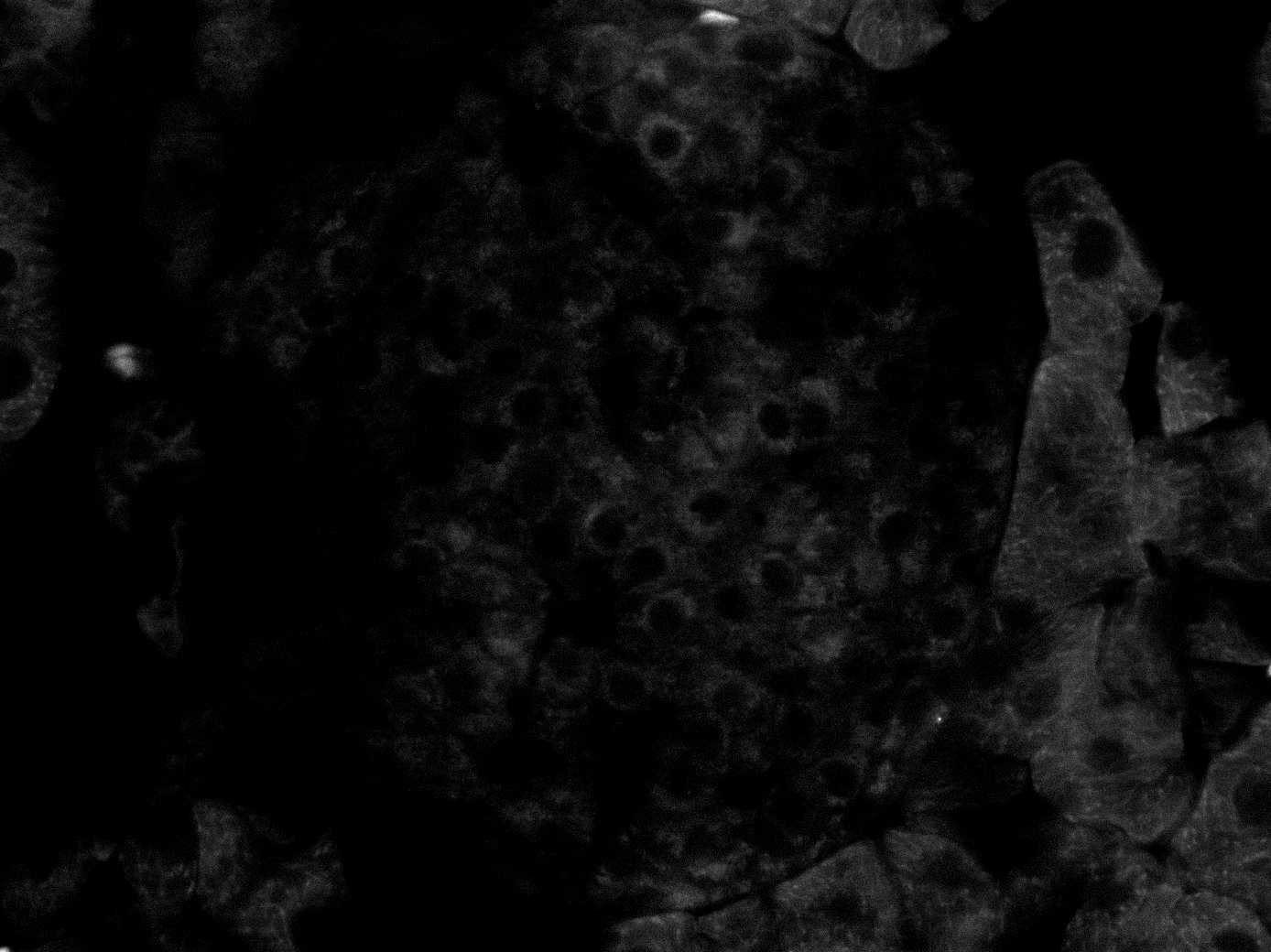

Supplement: Supplementary file 17 — Source Data for Figure 4 [file EMBJ-42-e113928-s006.zip › Figure 4/4I/CTRL TFEB.jpg]

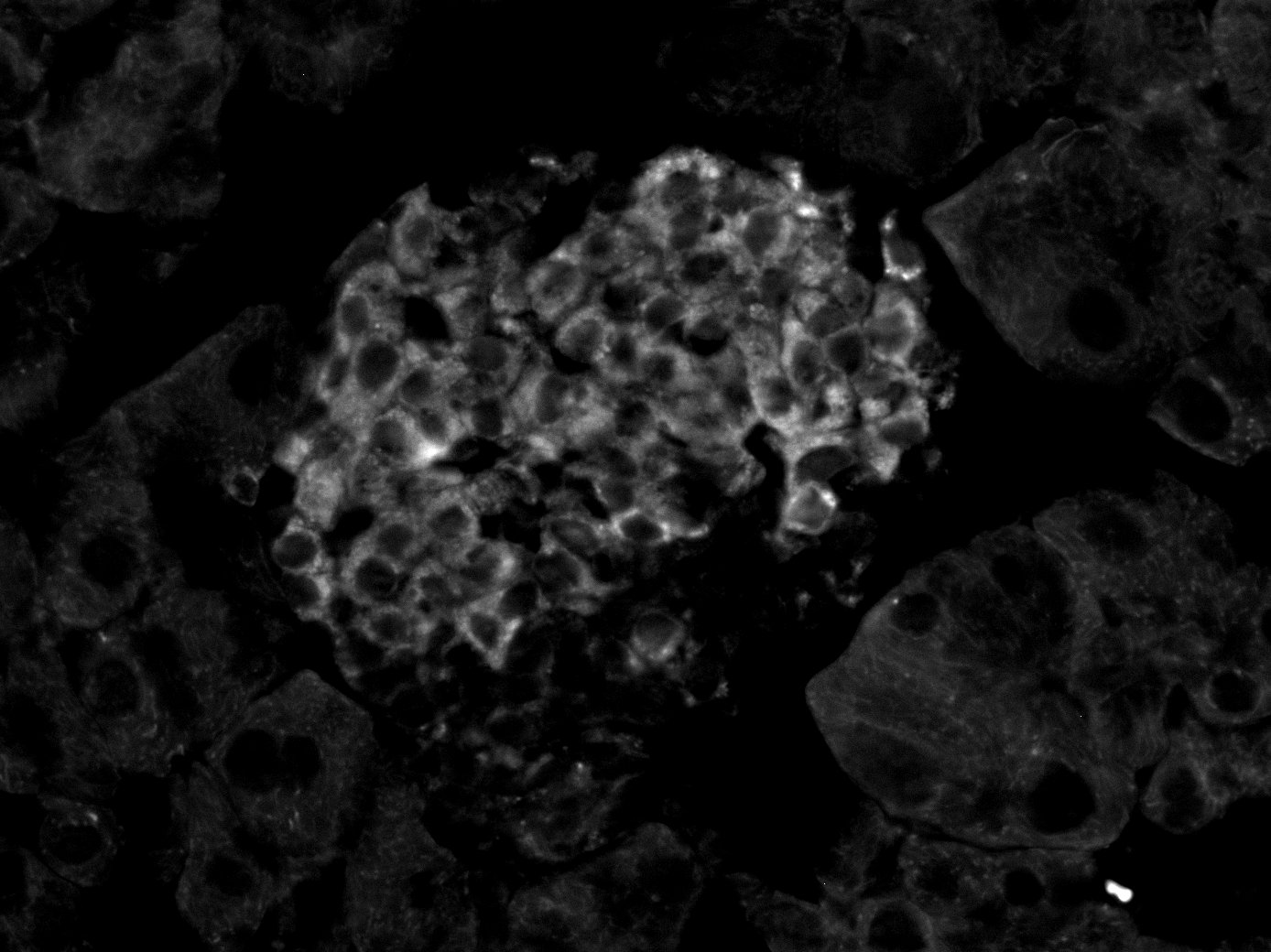

Supplement: Supplementary file 17 — Source Data for Figure 4 [file EMBJ-42-e113928-s006.zip › Figure 4/4I/TFEB OE TFEB.jpg]

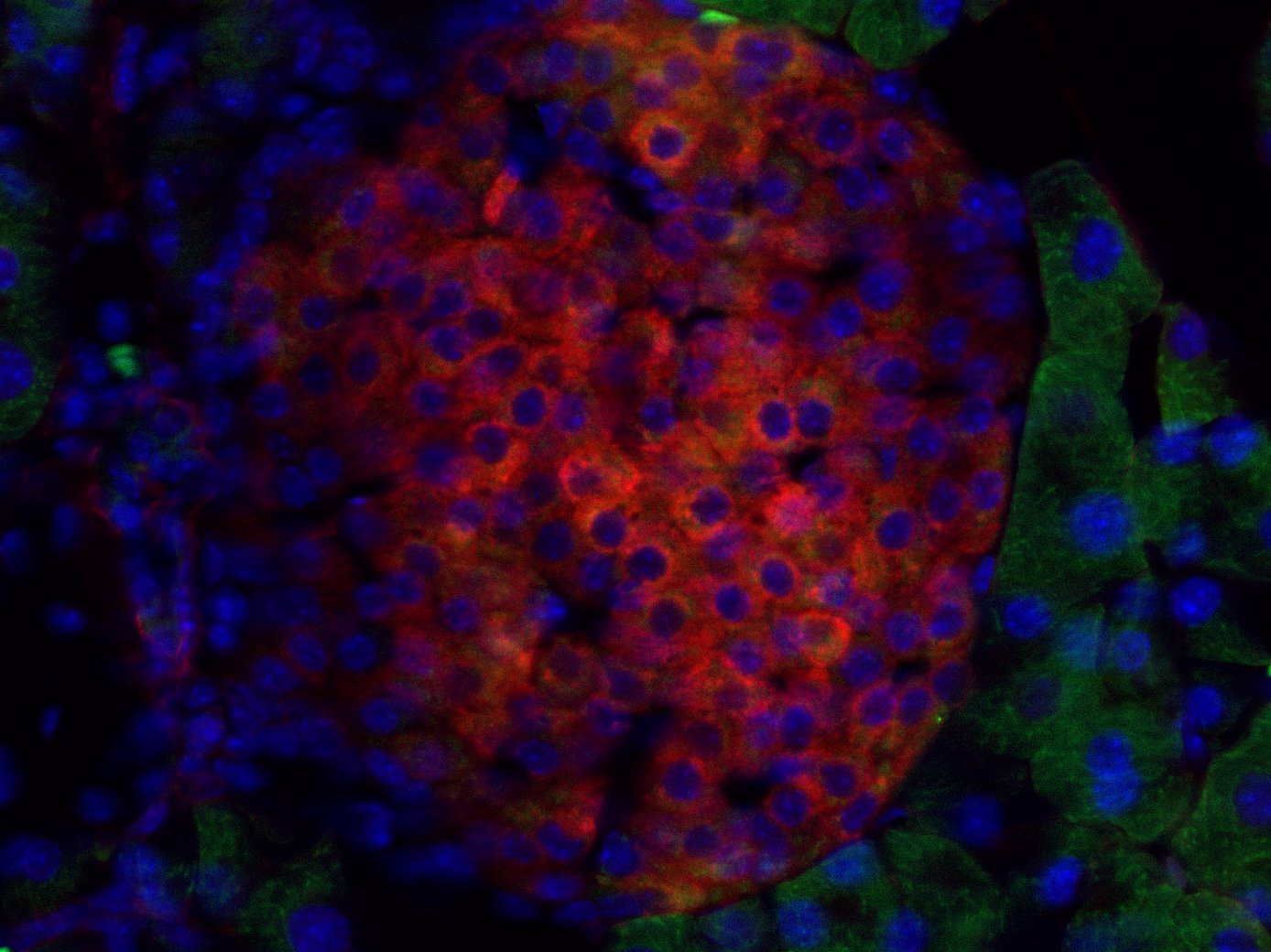

Supplement: Supplementary file 17 — Source Data for Figure 4 [file EMBJ-42-e113928-s006.zip › Figure 4/4I/CTRL MERGE.jpg]

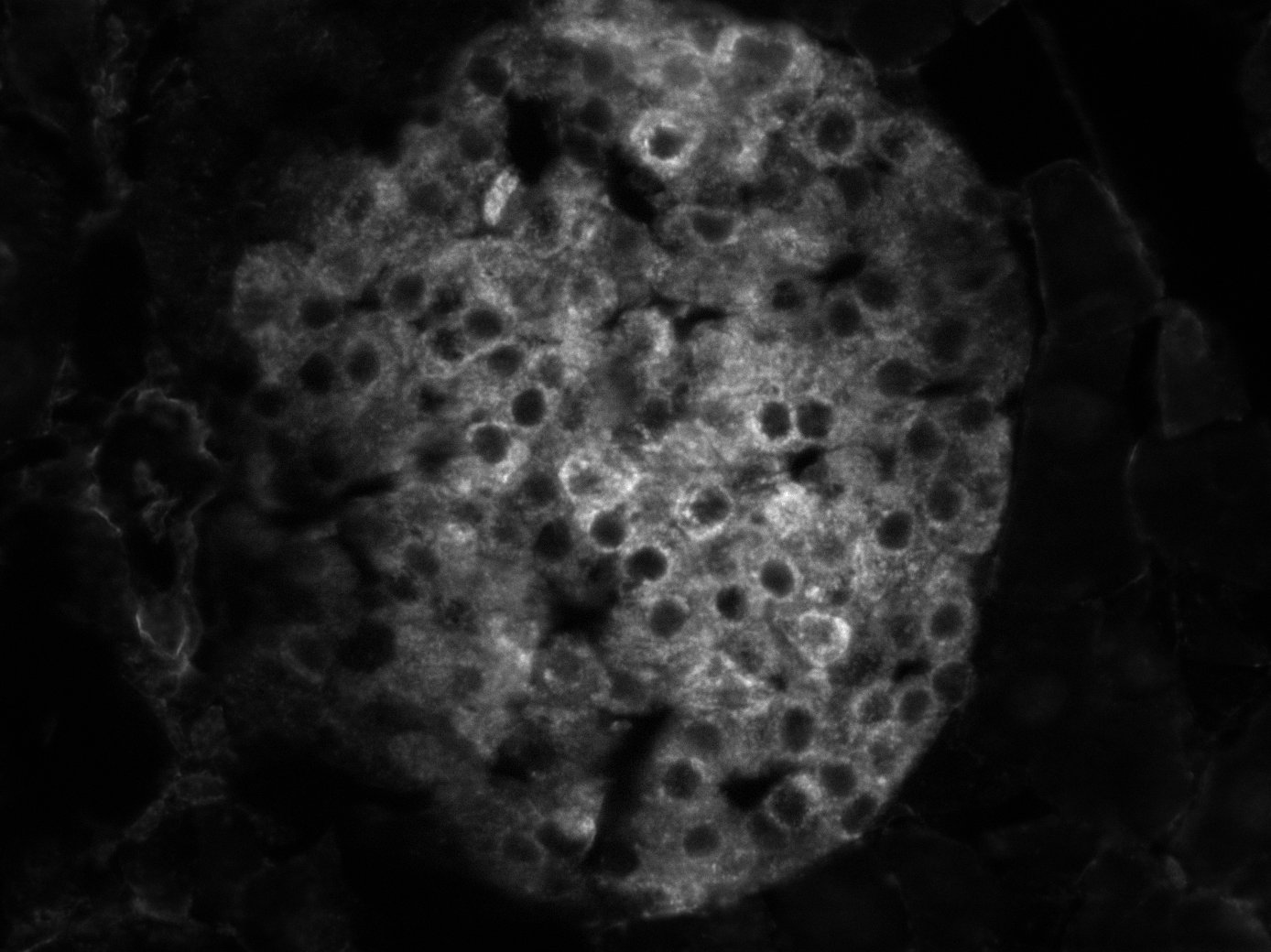

Supplement: Supplementary file 17 — Source Data for Figure 4 [file EMBJ-42-e113928-s006.zip › Figure 4/4I/CTRL UCN3.jpg]

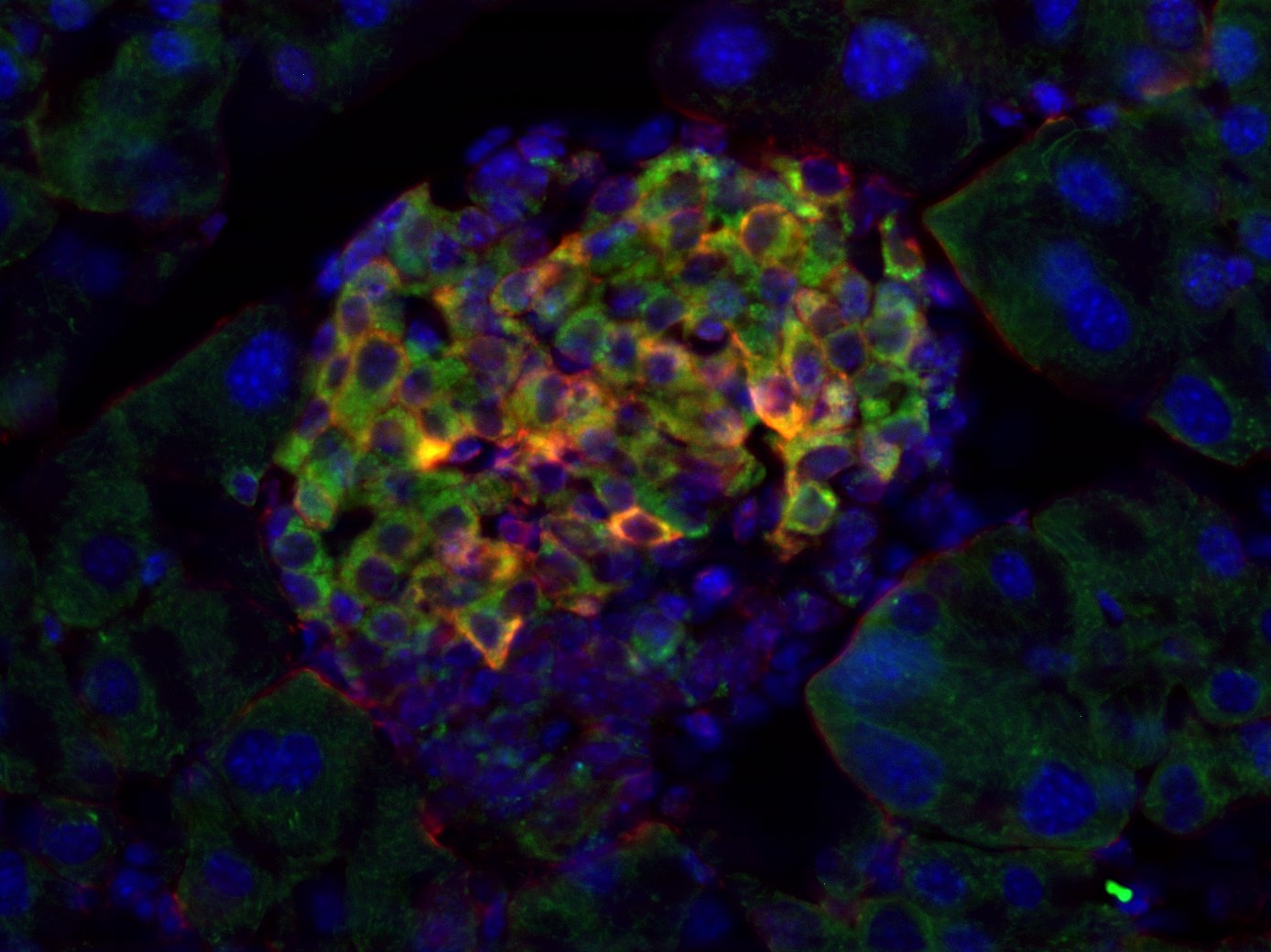

Supplement: Supplementary file 17 — Source Data for Figure 4 [file EMBJ-42-e113928-s006.zip › Figure 4/4I/TFEB OE MERGE.jpg]

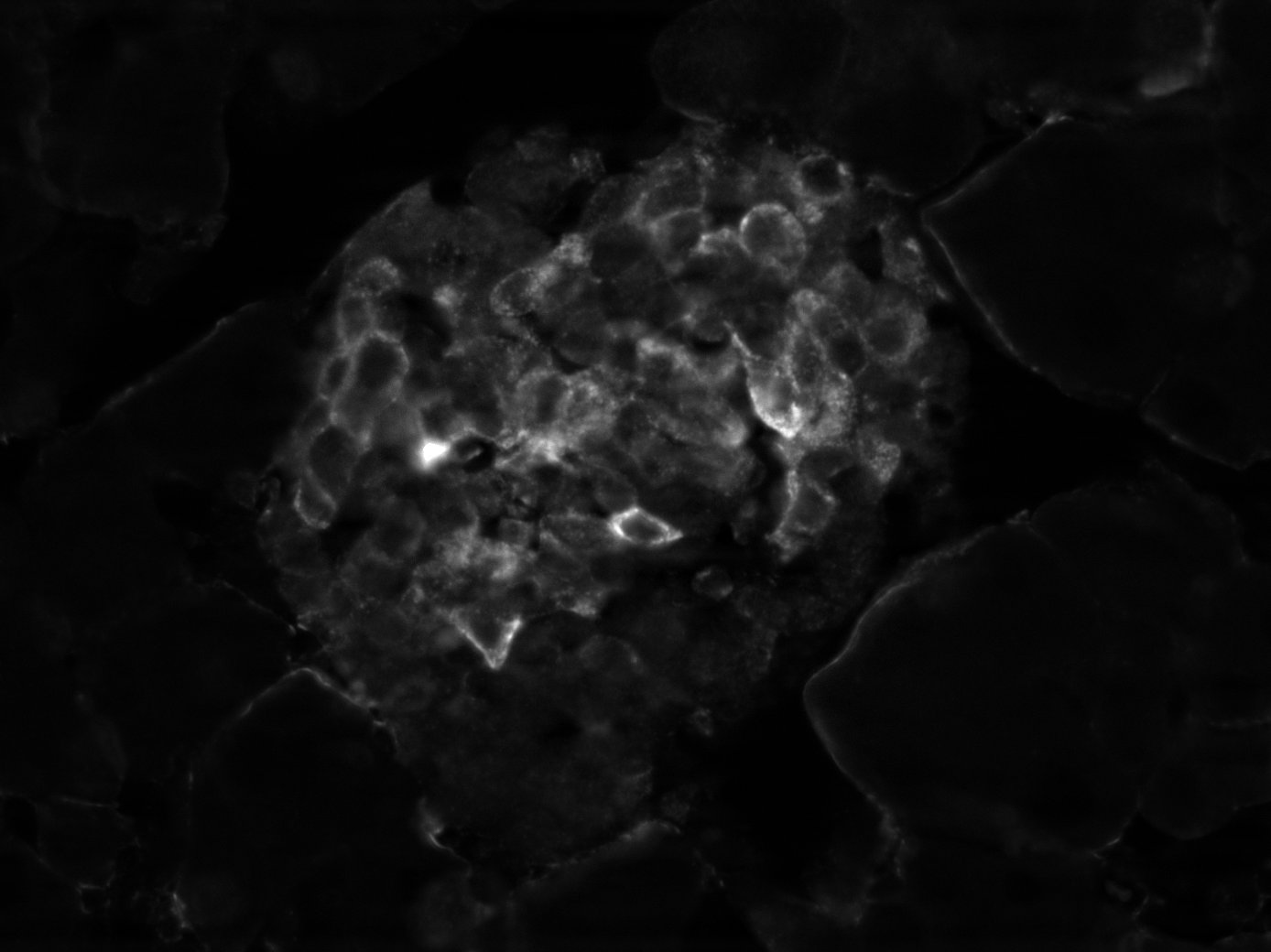

Supplement: Supplementary file 17 — Source Data for Figure 4 [file EMBJ-42-e113928-s006.zip › Figure 4/4I/TFEB OE UCN3.jpg]

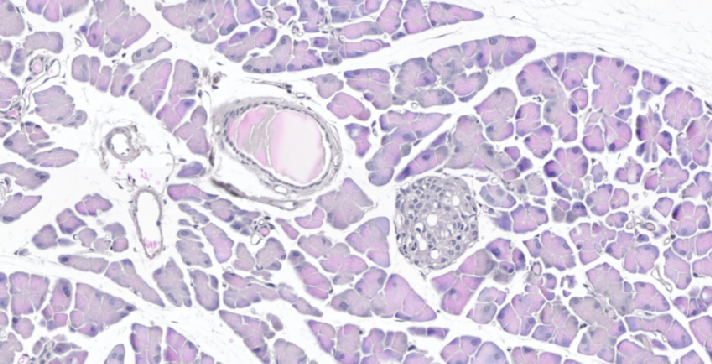

Supplement: Supplementary file 17 — Source Data for Figure 4 [file EMBJ-42-e113928-s006.zip › Figure 4/4G/TFEB OE.tif]

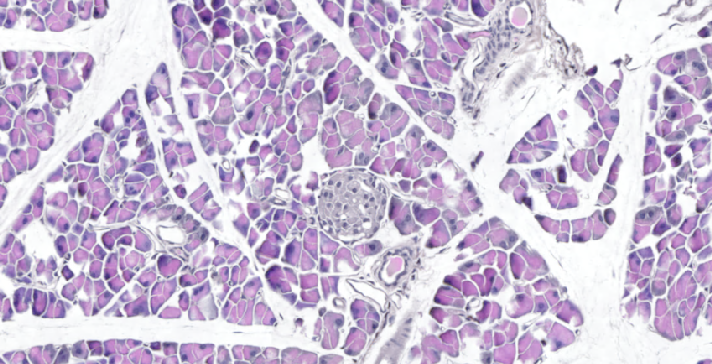

Supplement: Supplementary file 17 — Source Data for Figure 4 [file EMBJ-42-e113928-s006.zip › Figure 4/4G/CTRL.tif]

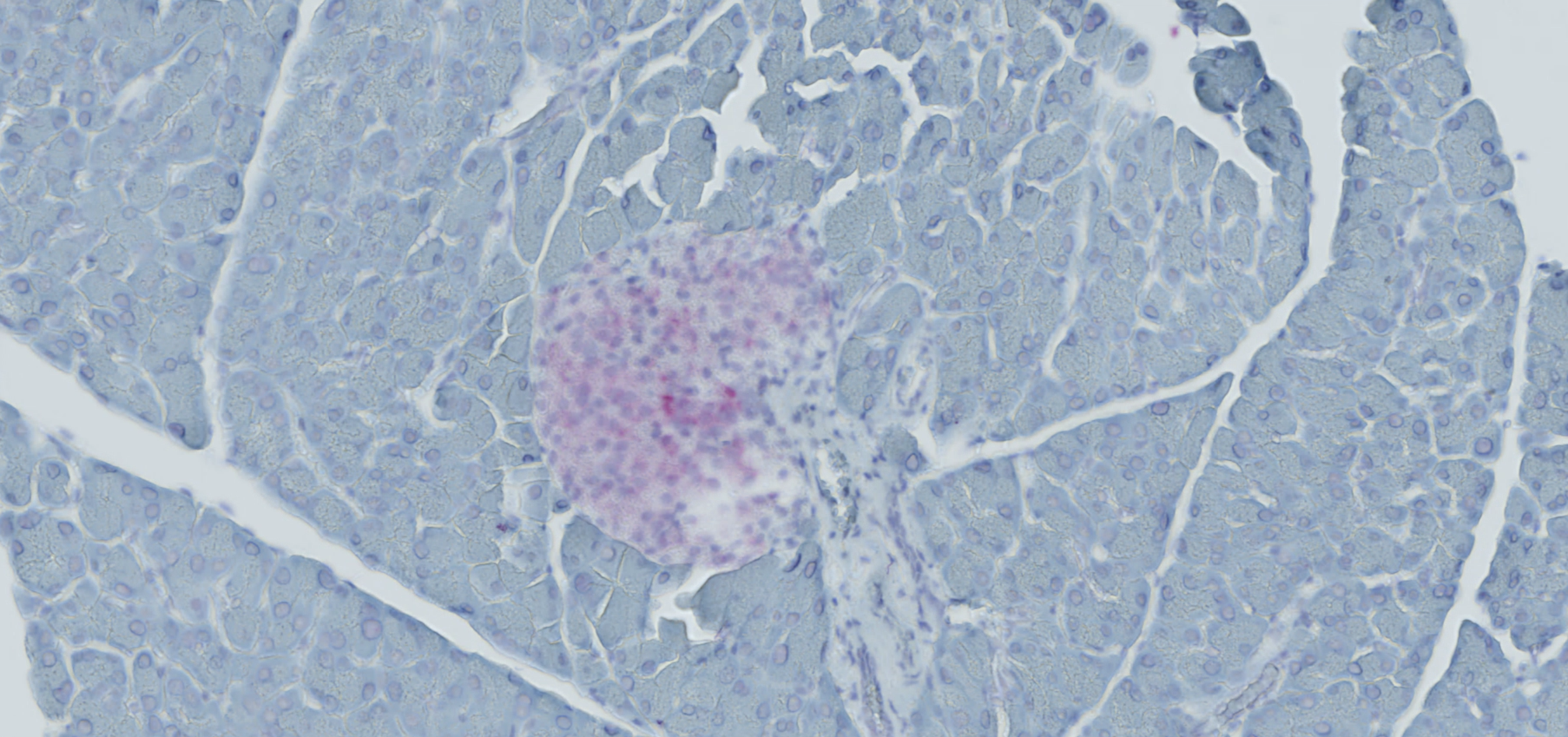

Supplement: Supplementary file 18 — Source Data for Figure 5 [file EMBJ-42-e113928-s017.zip › Figure 5/5A/CTRL STV.png]

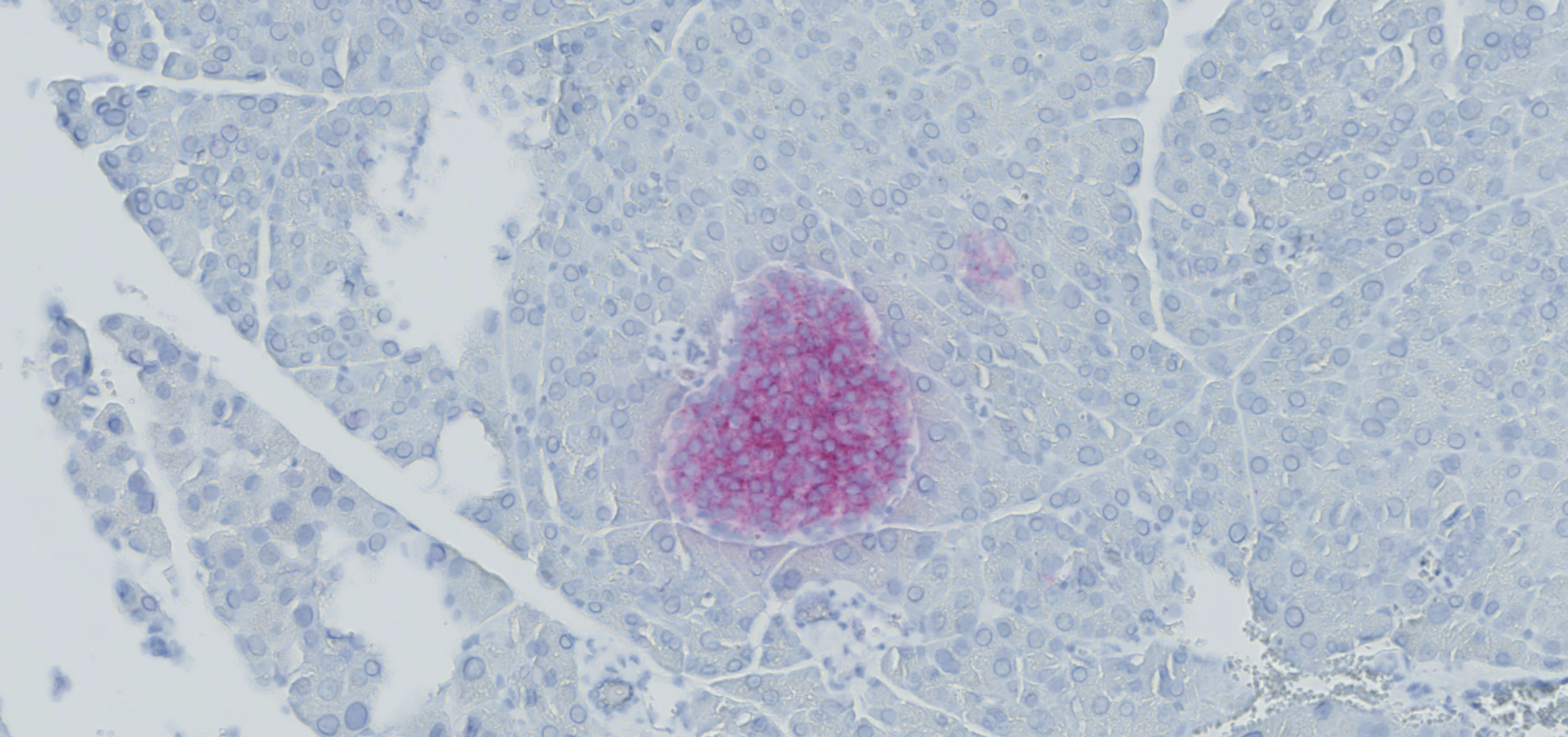

Supplement: Supplementary file 18 — Source Data for Figure 5 [file EMBJ-42-e113928-s017.zip › Figure 5/5A/CTRL Fed.png]

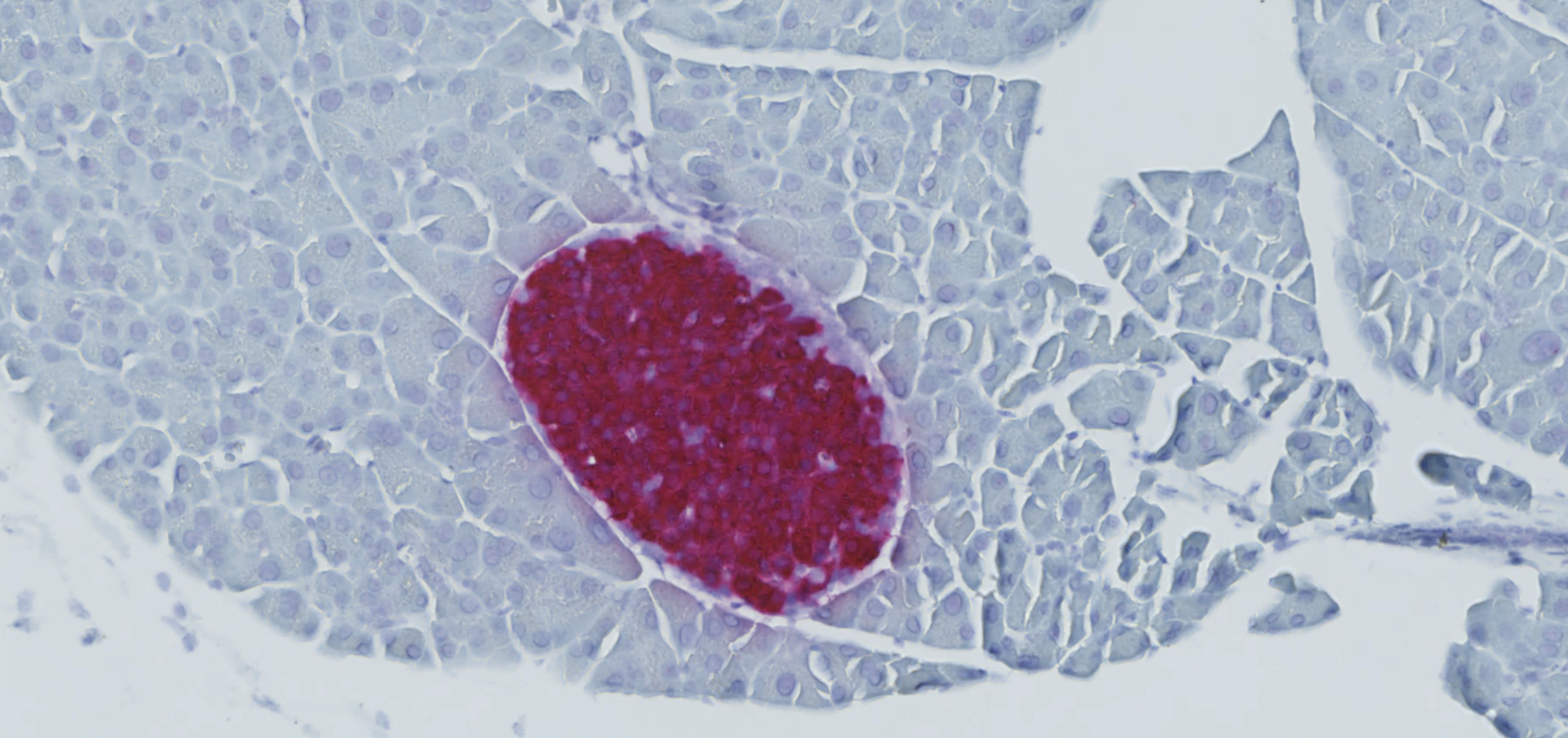

Supplement: Supplementary file 18 — Source Data for Figure 5 [file EMBJ-42-e113928-s017.zip › Figure 5/5A/DKO fed.png]

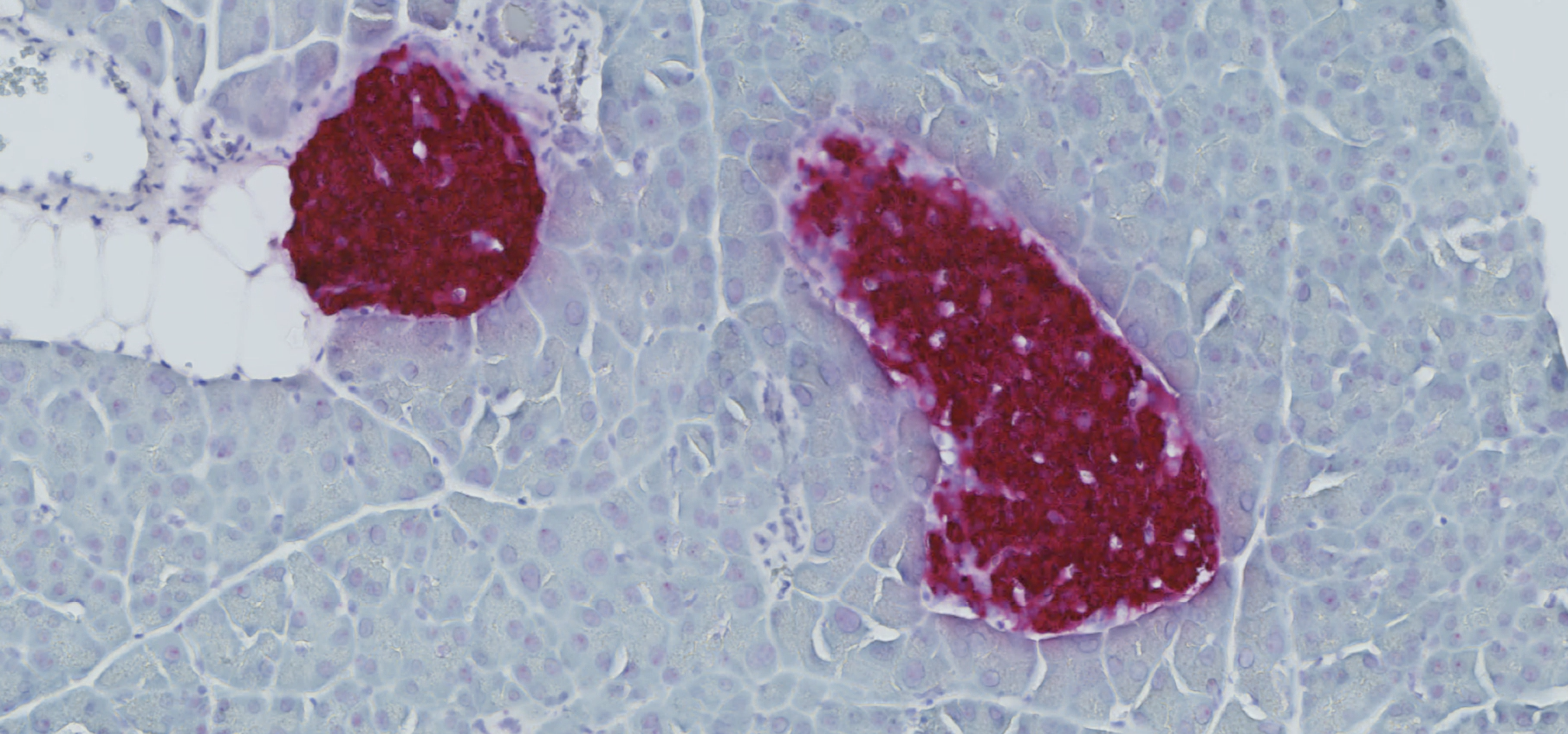

Supplement: Supplementary file 18 — Source Data for Figure 5 [file EMBJ-42-e113928-s017.zip › Figure 5/5A/DKO STV.png]

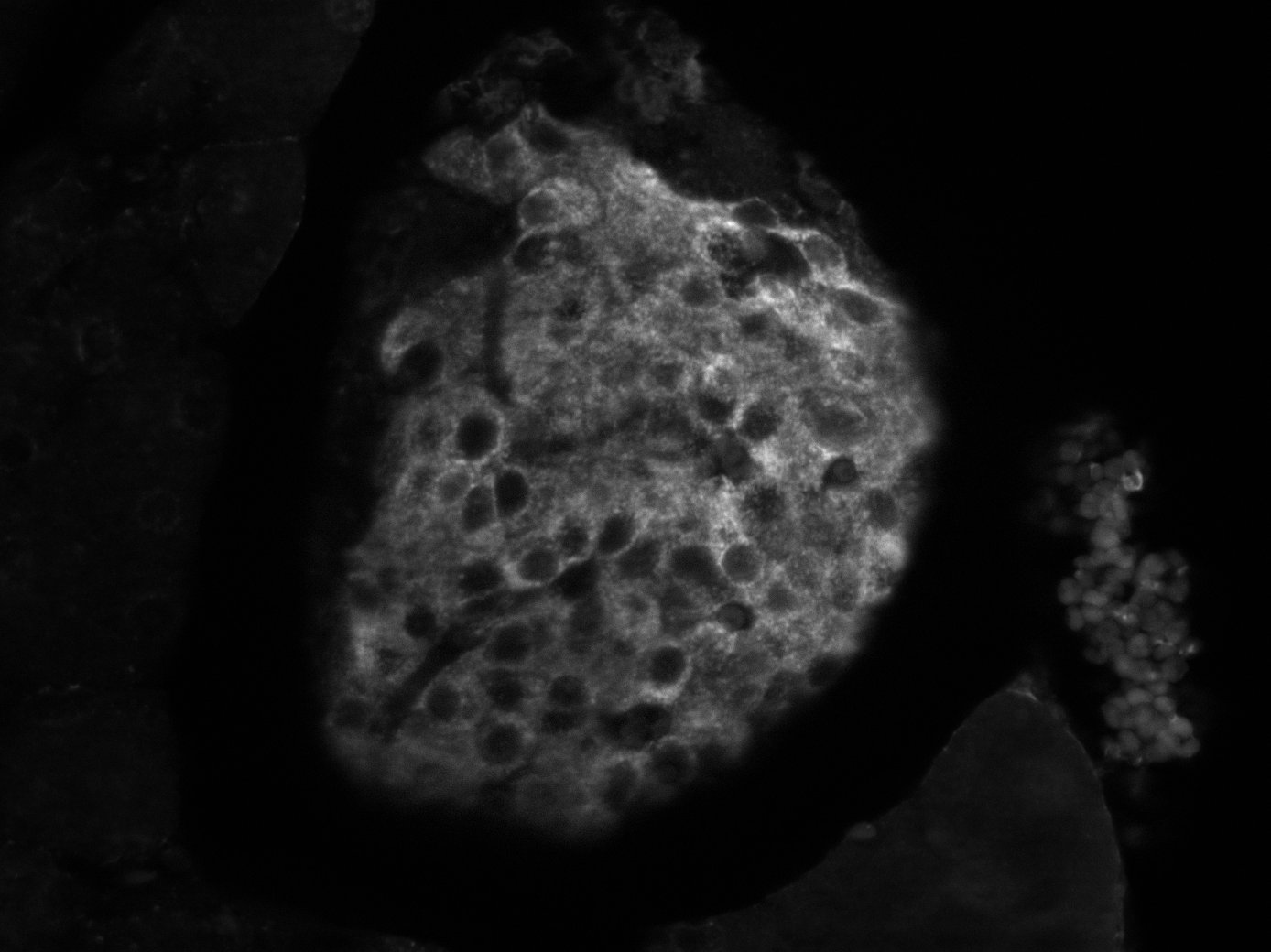

Supplement: Supplementary file 18 — Source Data for Figure 5 [file EMBJ-42-e113928-s017.zip › Figure 5/5C/DKO UCN3.jpg]

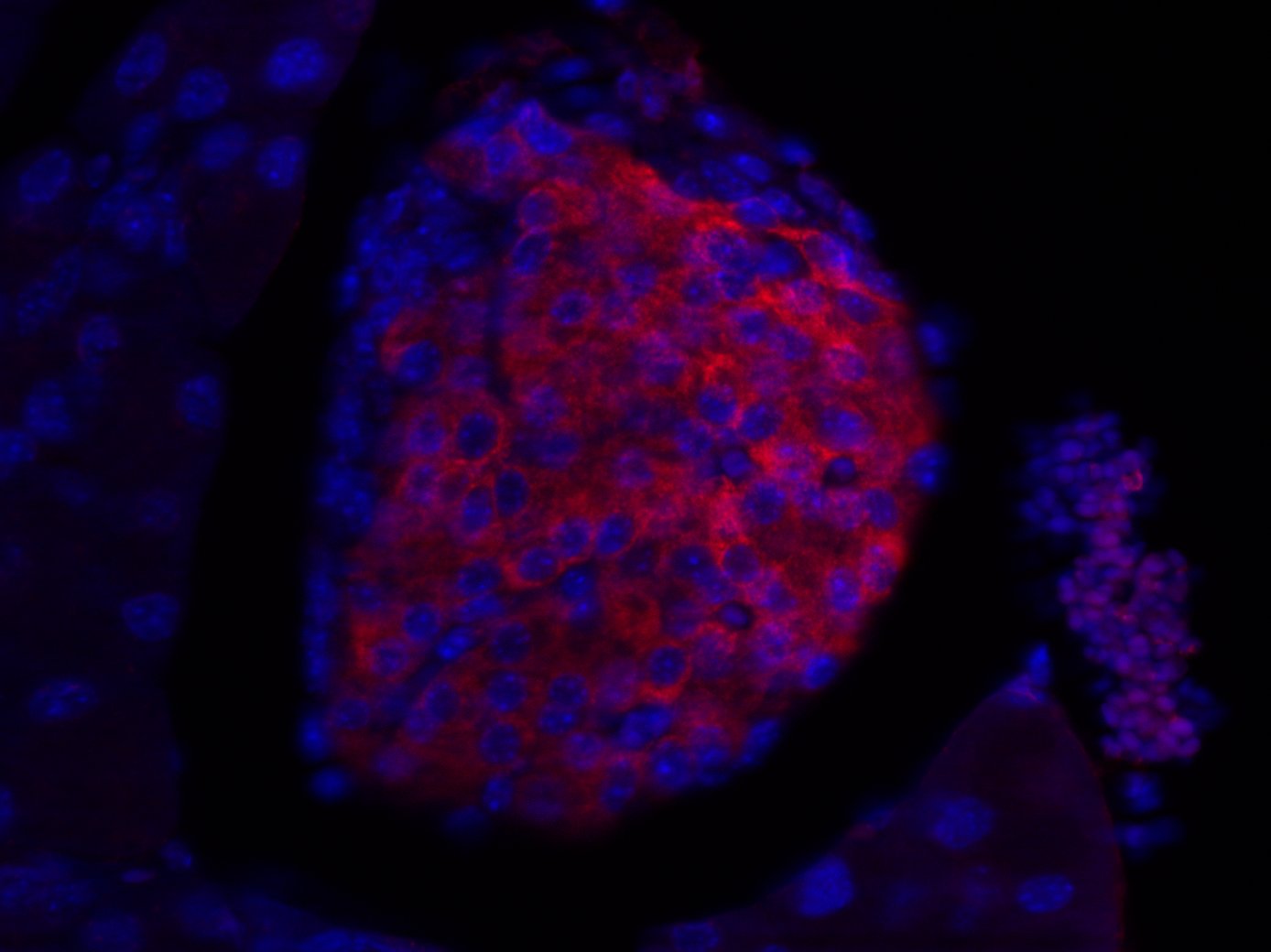

Supplement: Supplementary file 18 — Source Data for Figure 5 [file EMBJ-42-e113928-s017.zip › Figure 5/5C/DKO MERGE.jpg]

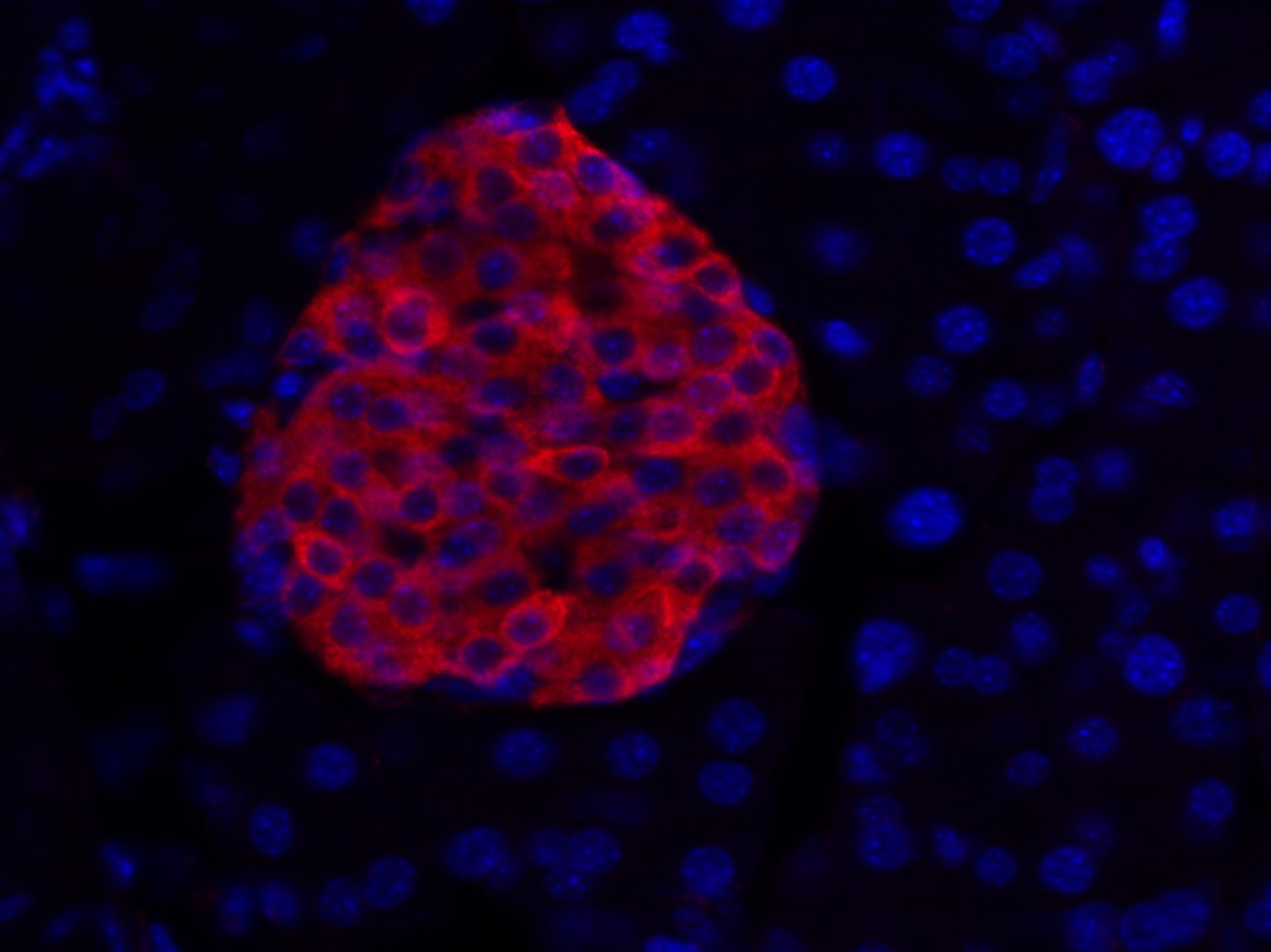

Supplement: Supplementary file 18 — Source Data for Figure 5 [file EMBJ-42-e113928-s017.zip › Figure 5/5C/CTRL MERGE.jpg]

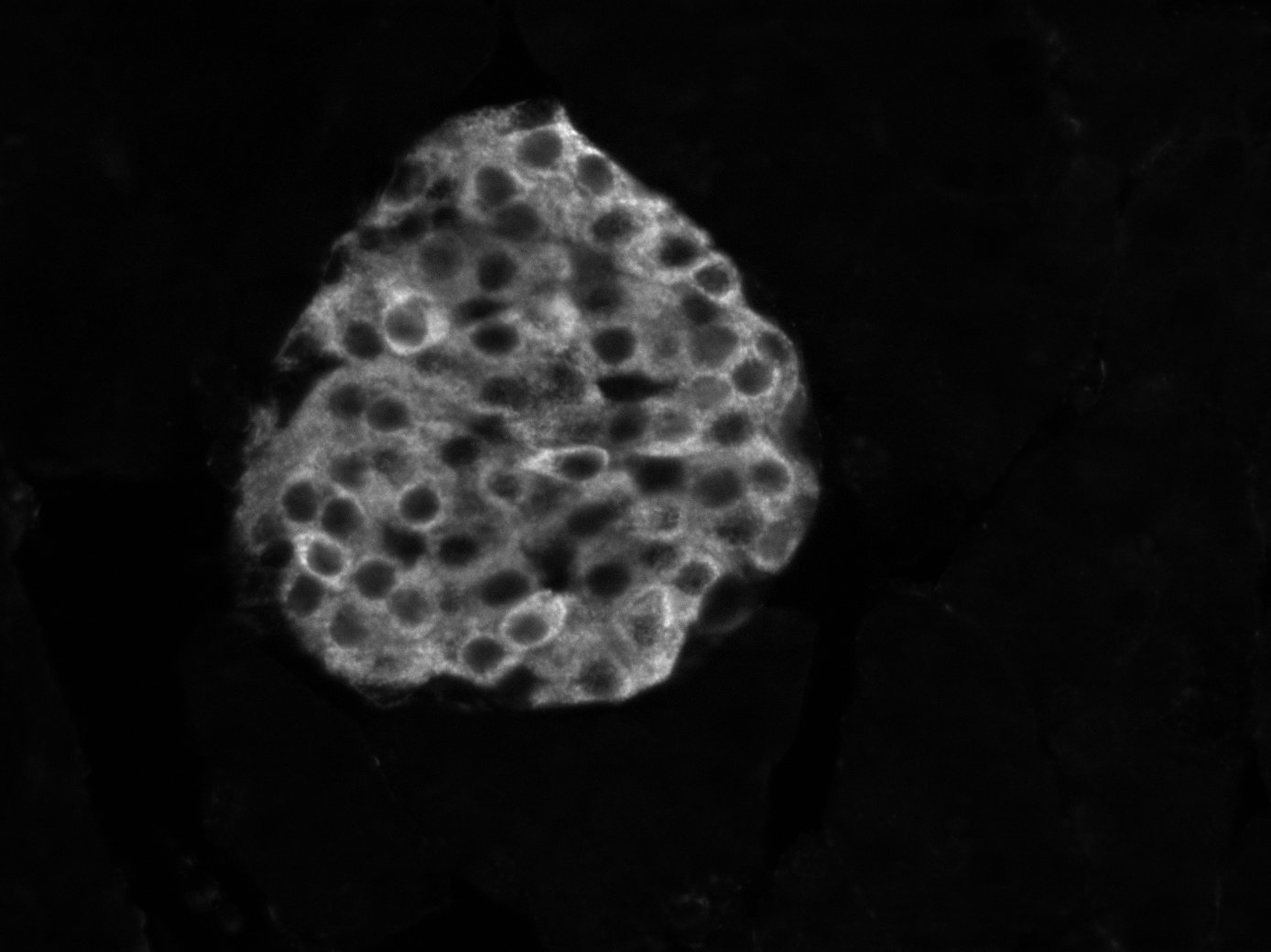

Supplement: Supplementary file 18 — Source Data for Figure 5 [file EMBJ-42-e113928-s017.zip › Figure 5/5C/CTRL UCN3.jpg]

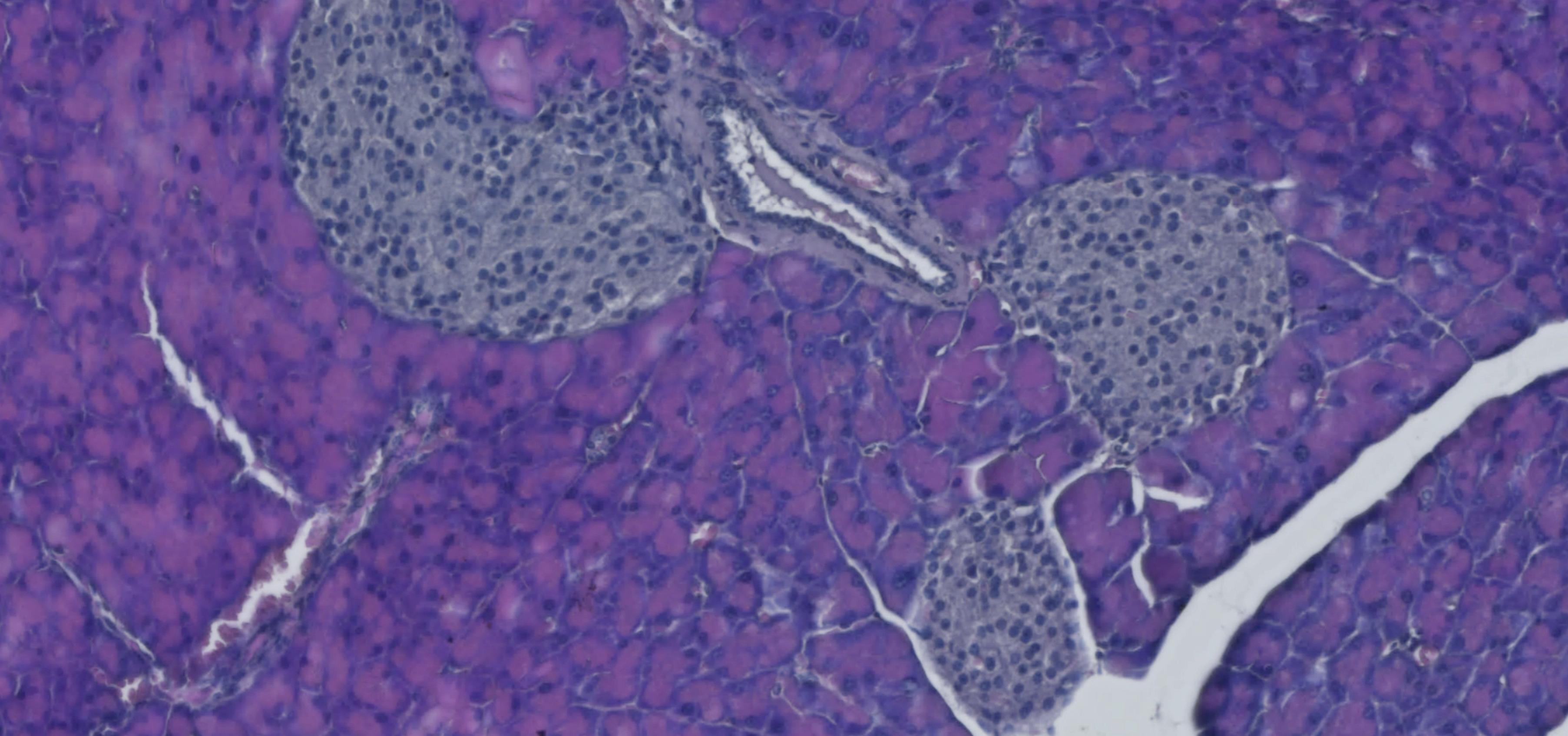

Supplement: Supplementary file 18 — Source Data for Figure 5 [file EMBJ-42-e113928-s017.zip › Figure 5/5B/CTRL.png]

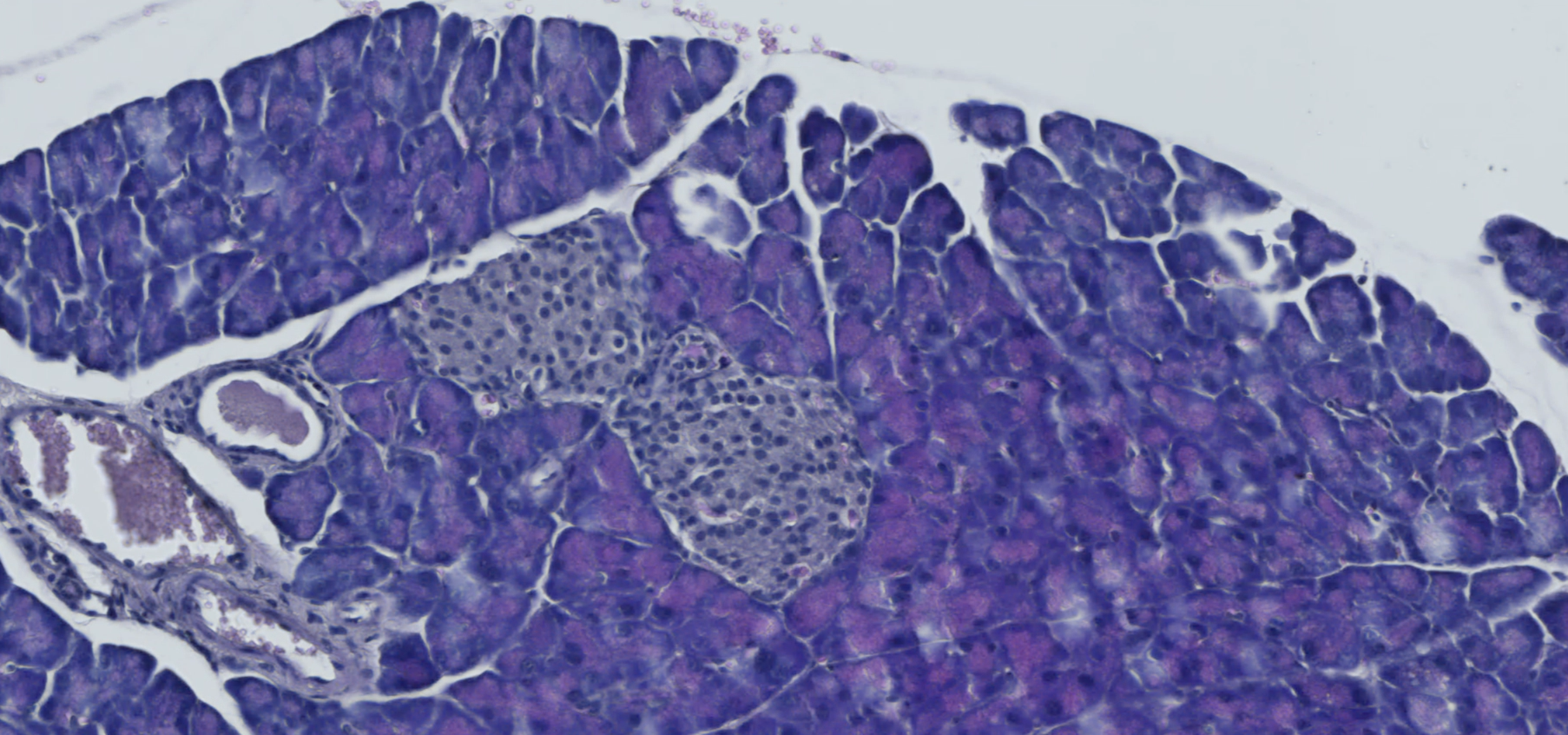

Supplement: Supplementary file 18 — Source Data for Figure 5 [file EMBJ-42-e113928-s017.zip › Figure 5/5B/DKO.png]
